# Alternative polyadenylation reprogramming of MORC2 induced by NUDT21 loss promotes KIRC carcinogenesis

**DOI:** 10.1172/jci.insight.162893

**Published:** 2023-09-22

**Authors:** Yuqin Tan, Tong Zheng, Zijun Su, Min Chen, Suxiang Chen, Rui Zhang, Ruojiao Wang, Ke Li, Ning Na

**Affiliations:** 1 Department of Kidney Transplantation, The Third Affiliated Hospital of Sun Yat-sen University, Guangzhou, Guangdong, China.; 2 The First Affiliated Hospital, Faculty of Medical Science, Jinan University, Guangzhou, Guangdong, China.; 3 Department of Stomatology, Nanfang Hospital, Southern Medical University, Guangzhou, Guangdong, China.; 4 Centre for Molecular Medicine and Innovative Therapeutics, Murdoch University, Perth, Western Australia, Australia.; 5 Department of Urology, The Third Affiliated Hospital of Sun Yat-sen University, Guangzhou, Guangdong, China.

**Keywords:** Oncology, Cancer, Oncogenes, Tumor suppressors

## Abstract

Alternative polyadenylation (APA), a posttranscriptional mechanism of gene expression via determination of 3′UTR length, has an emerging role in carcinogenesis. Although abundant APA reprogramming is found in kidney renal clear cell carcinoma (KIRC), which is one of the major malignancies, whether APA functions in KIRC remains unknown. Herein, we found that chromatin modifier MORC2 gained oncogenic potential in KIRC among the genes with APA reprogramming, and moreover, its oncogenic potential was enhanced by 3′UTR shortening through stabilization of MORC2 mRNA. MORC2 was found to function in KIRC by downregulating tumor suppressor DAPK1 via DNA methylation. Mechanistically, MORC2 recruited DNMT3A to facilitate hypermethylation of the DAPK1 promoter, which was strengthened by 3′UTR shortening of MORC2. Furthermore, loss of APA regulator NUDT21, which was induced by DNMT3B-mediated promoter methylation, was identified as responsible for 3′UTR shortening of MORC2 in KIRC. Additionally, NUDT21 was confirmed to act as a tumor suppressor mainly depending on downregulation of MORC2. Finally, we designed an antisense oligonucleotide (ASO) to enhance NUDT21 expression and validated its antitumor effect in vivo and in vitro. This study uncovers the DNMT3B/NUDT21/APA/MORC2/DAPK1 regulatory axis in KIRC, disclosing the role of APA in KIRC and the crosstalk between DNA methylation and APA.

## Introduction

Renal cell carcinoma (RCC) is one of leading malignancies worldwide, and kidney renal clear cell carcinoma (KIRC), which accounts for more than 70% of RCC, is the most prevailing subtype (1–3). Due to the difficulties in early diagnosis and treatment, KIRC is characteristic for poor prognosis. Moreover, KIRC displays high rates of chemoresistance and even low rates of immunotherapy response (4). Therefore, to develop novel and feasible approaches of early diagnosis and targeted therapy, in-depth investigation into mechanism of KIRC carcinogenesis is in great need.

Alternative polyadenylation (APA), an indispensable posttranscriptional regulation of gene expression, has increasingly gained attention in pathogenesis — especially carcinogenesis. At least 70% of mammalian genes own multiple polyA sites (PAS) that lead to production of mRNA isoforms with varying lengths of 3′UTRs (5–7). Given that the 3′UTR acts as a major binding region for microRNA and RNA binding protein (RBP), APA makes a great difference in mRNA stability, translation, and translocation by determining 3′UTR length (8). In carcinogenesis, APA reprogramming, especially 3′UTR shortening, is common (9, 10), mainly playing a role in oncogene overactivation (11, 12). APA homeostasis substantially relies on coordination of APA regulators (13), and therefore, dysregulation of key APA regulators is responsible for most APA reprogramming in cancers. For example, NUDT21, the most important APA regulator favoring the long 3′UTR isoform (8, 14), is lost in some types of cancer. In addition, other epigenetic mechanisms, especially DNA methylation, also make a difference in APA regulation. Previously, DNA methylation was shown to promote use of the proximal polyadenylation site via enabling CCCTC binding factor (CTCF) binding and recruitment of the cohesin complex (15). However, the interaction between DNA methylation and APA remains largely unknown.

Nevertheless, in KIRC, whether APA functioned remains unknown. A previous study identified abundant APA reprogramming in multiple types of cancer, including KIRC (16), implying APA’s potential role in KIRC. Therefore, this study investigated whether and how APA functioned in KIRC in order to develop a potentially novel therapeutic strategy for this type of cancer.

Herein, we found that, among the genes with shortened 3′UTR in KIRC, MORC2 gained oncogenic potential, and 3′UTR shortening enhanced its oncogenic potential via stabilization of MORC2 mRNA. Furthermore, MORC2 promoted KIRC carcinogenesis by epigenetically silencing the tumor suppressor DAPK1. Specifically, MORC2 recruited DNMT3A to induce hypermethylation of the DAPK1 promoter, which was strengthened by 3′UTR shortening of MORC2. Next, we identified that loss of the APA regulator NUDT21 was responsible for 3′UTR shortening of MORC2 and epigenetic loss of DAPK1 in KIRC. This indicated, in turn, that APA dysregulation impacted DNA methylation. We also showed that NUDT21 was lost in KIRC due to hypermethylation of DNMT3B-mediated promoter hypermethylation, uncovering a potentially novel role of DNA methylation in APA regulation. Moreover, NUDT21 was shown to function as a tumor suppressor in KIRC mainly dependent on MORC2 downregulation. Finally, we designed an antisense oligonucleotide (ASO) to enhance NUDT21 expression and validated that it was effective in repressing proliferation and tumor formation of KIRC cells. Taken together, this study uncovered the DNMT3B/NUDT21/APA/MORC2/DAPK1 prooncogenic axis in KIRC, demonstrating how APA functions in KIRC and improving the insights into the crosstalk between DNA methylation and APA.

## Results

### Among the genes with shortened 3′UTRs in KIRC, UCK2 and MORC2 gain oncogenic potential.

**Figure 1:**
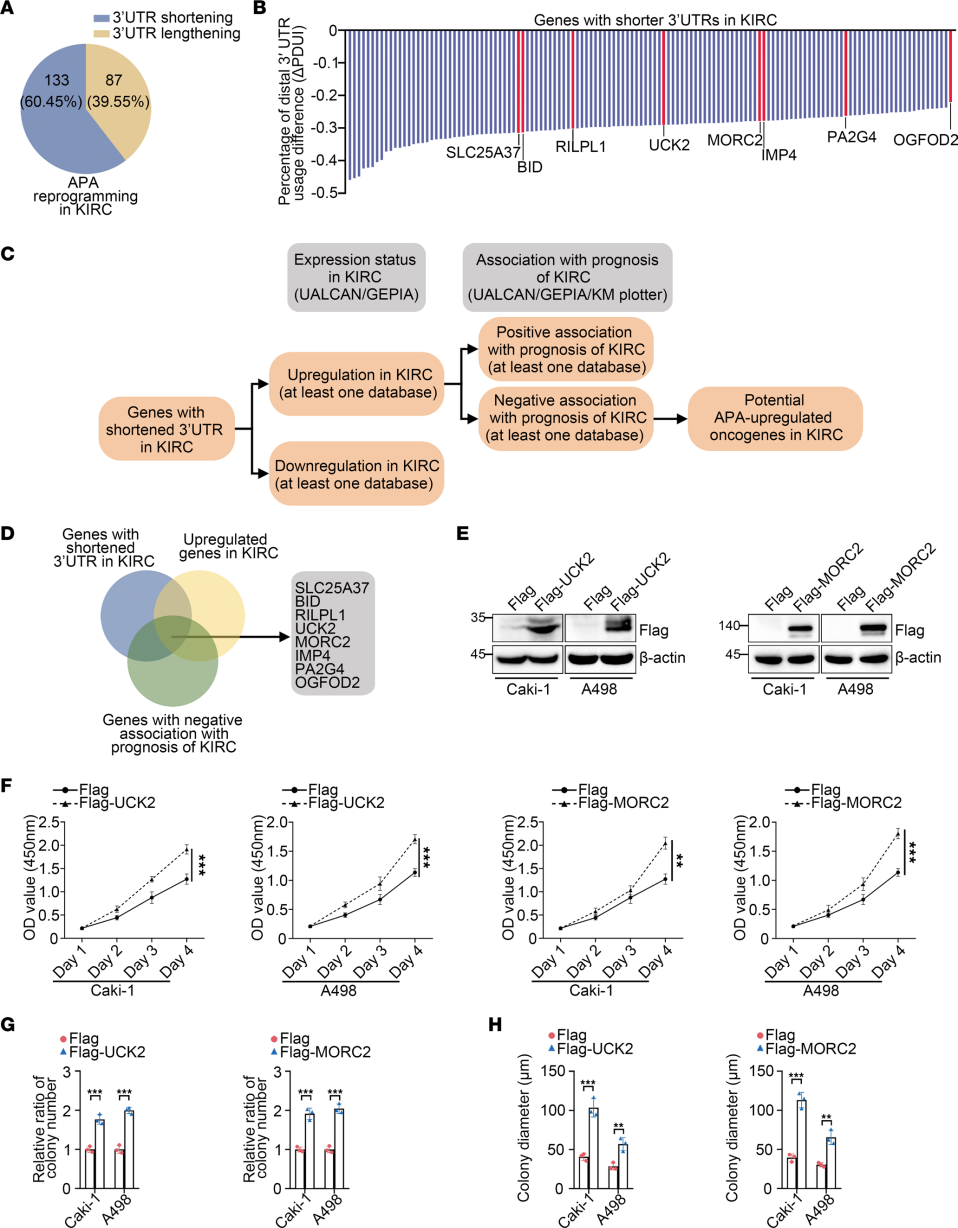
Among the genes with shortened 3′UTRs in KIRC, *UCK2* and *MORC2* gain oncogenic potential. (**A**) The diagram indicating the composition of 3′UTR reprogramming in KIRC. (**B**) The quantitative summary of genes with shortened 3′UTRs in KIRC. (**C**) The sketch map indicating the procedure of bioinformatic analysis to identify potential oncogenes of KIRC. (**D**) The Venn diagram indicating the potential oncogenes among genes with shortened 3′UTRs in KIRC. (**E**) Immunoblotting was performed to evaluate the expression of Flag-UCK2 and Flag-MORC2 in Caki-1 and A498 cells transfected with empty Flag vector, Flag-UCK2, or Flag-MORC2 plasmid. (**F**) CCK8 assays were performed to evaluate the proliferation rate of Caki-1 cells and A498 cells transfected with empty Flag vector, Flag-UCK2, or Flag-MORC2 plasmid (*n* = 3). (**G**) Colony formation assays were performed and quantitatively analyzed to evaluate the clonogenicity of Caki-1 cell and A498 cells transfected with empty Flag vector, Flag-UCK2, or Flag-MORC2 plasmid (*n* = 3). (**H**) Soft agar assays were performed and quantitatively analyzed to evaluate the clonogenicity of Caki-1 cell and A498 cells transfected with empty Flag vector, Flag-UCK2, or Flag-MORC2 plasmid (*n* = 3). All data represent the mean ± SD. Two-tailed *t* test analyses were performed. **P < 0.01; ***P < 0.001.

APA has emerged as a participator in carcinogenesis in more types of cancers. However, in KIRC, whether or not and how APA may function remained unexplored. A previous investigation of APA identified numerous genes that undergo APA reprogramming in 7 types of cancer, including KIRC (16), suggesting a potential role of APA in KIRC. To investigate APA’s potential effect in KIRC, we analyzed the genes with altered 3′UTRs in KIRC. Generally, over 200 genes with shortened or lengthened 3′UTRs were found in KIRC. Consistent with most types of cancers, 3′UTR shortening represents the majority of APA reprogramming in KIRC (Figure 1, A and B, and Supplemental Data 1; supplemental material available online with this article; https://doi.org/10.1172/jci.insight.162893DS1). APA plays a prooncogenic role mainly via shortening 3′UTRs of oncogenes; therefore, we wondered whether APA also upregulated oncogenes in KIRC. To this end, we first screened the 3′UTR-shortened genes to identify known KIRC oncogenes. However, no canonical KIRC oncogenes were found among these genes. This led us to determine whether currently unknown KIRC oncogenes existed among the shortened genes. A bioinformation-based screen was conducted to reduce the scope of oncogene identification (Figure 1C). Briefly, based on the data of 3′UTR-shortened genes, including expression status and prognosis association obtained from the UALCAN (17), GEPIA (18), and Kaplan-Meier (KM) plotter databases (19), genes could be considered as potential KIRC oncogenes if they displayed both upregulation and a negative association with prognosis in KIRC in at least 1 database. However, to lessen controversy, the candidates that were found downregulated in KIRC — or associated positively with prognosis of KIRC even in 1 database — were excluded from the list of potential oncogenes. According to this criteria, 8 genes, including SLC25A37, BID, UCK2, MORC2, IMP4, PA2G4, OGFOD2, and RILPL1, were selected (Figure 1, B and D).

The oncogenic potential of these candidates was then examined in vitro. As an initial screen, CCK8 assays indicated that, compared with the similar Flag-transfected cells, only transfection of Flag-UCK2 or Flag-MORC2 significantly promoted proliferation of both Caki-1 and A498 KIRC cells (Figure 1, E and F, and Supplemental Figure 1). This suggested UCK2 and MORC2 as potential KIRC oncogenes. Colony formation and soft agar assays were also performed and further validated the oncogenic potential of UCK2 and MORC2 in KIRC cells (Figure 1, G and H, and Supplemental Figure 2). These data suggest that APA might function in KIRC by inducing 3′UTR shortening of UCK2 and MORC2.

### 3′UTR shortening enhances the oncogenic potential of MORC2 in KIRC by upregulating MORC2.

**Figure 2:**
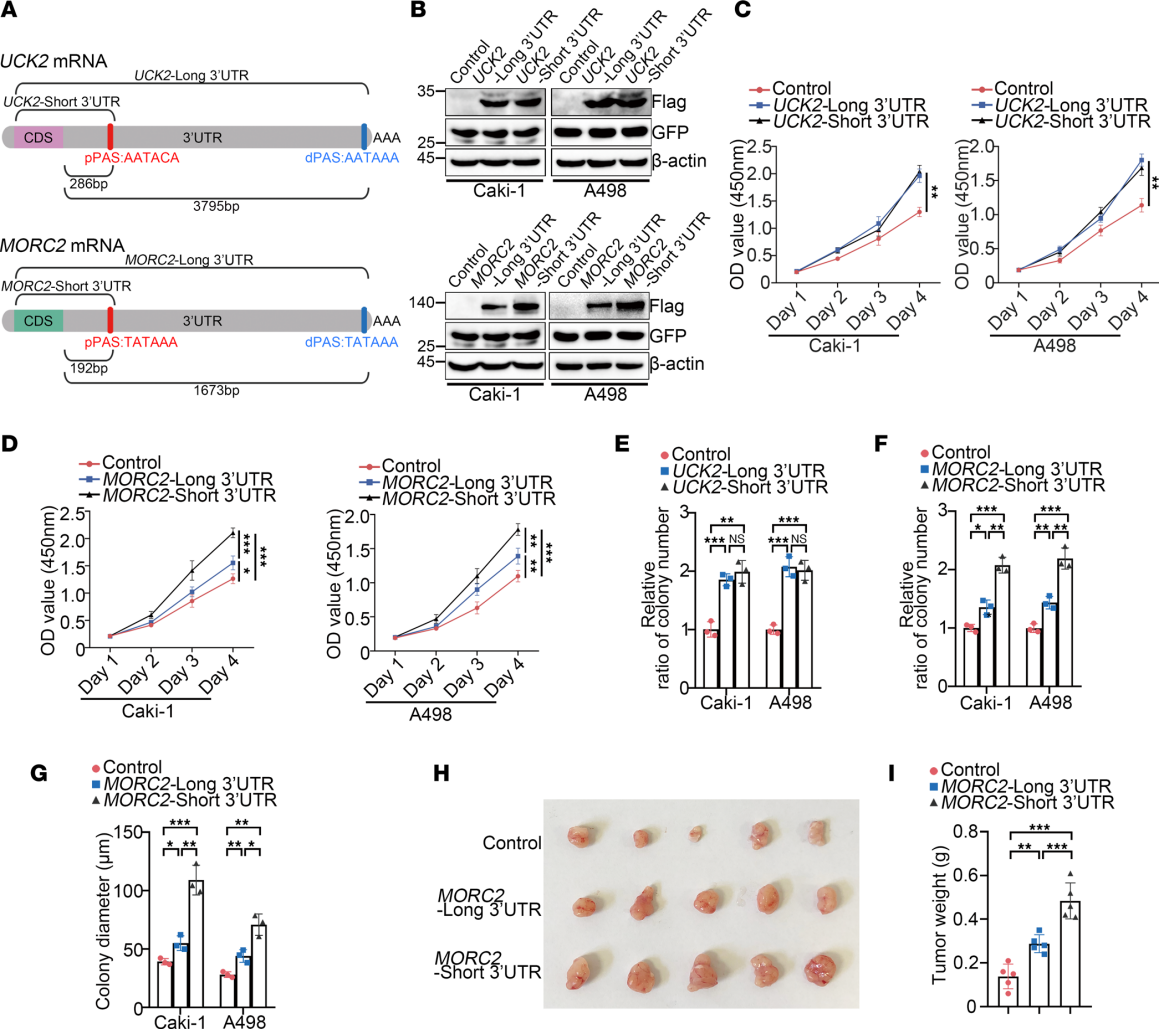
3′UTR shortening enhances the oncogenic potential of *MORC2* in KIRC by upregulating MORC2. (**A**) The schematic diagram indicating the proximal polyA sites of MORC2 and UCK2. (**B**) Immunoblotting was performed to evaluate the expression of Flag-UCK2, Flag-MORC2, and GFP in Caki-1 and A498 cells transfected with indicated plasmids. (**C**) CCK8 assays were performed to evaluate the proliferation rate of Caki-1 cells and A498 cells transfected with short or long 3′UTR UCK2 plasmid (*n* = 3). (**D**) CCK8 assays were performed to evaluate the proliferation rate of Caki-1 cells and A498 cells transfected with short or long 3′UTR MORC2 plasmid (*n* = 3). (**E**) Colony formation assays were performed and quantitatively analyzed to evaluate the clonogenicity of Caki-1 cell and A498 cells transfected with short or long 3′UTR UCK2 plasmid (*n* = 3). (**F**) Colony formation assays were performed and quantitatively analyzed to evaluate the clonogenicity of Caki-1 cell and A498 cells transfected with short or long 3′UTR MORC2 plasmid (*n* = 3). (**G**) Soft agar assays were performed and quantitatively analyzed to evaluate the clonogenicity of Caki-1 cell and A498 cells transfected with short or long 3′UTR MORC2 plasmid (*n* = 3). (**H** and **I**) In vivo xenograft tumor formation experiment was performed (**H**) and quantitatively analyzed (**I**) with control, short 3′UTR, or long 3′UTR MORC2 stably expressed Caki-1 cells (*n* = 5 per group). All data represent the mean ± SD. Two-tailed *t* test or 1-way ANOVA with Tukey multiple-comparison test analyses were performed. *P < 0.05; **P < 0.01; ***P < 0.001.

We next investigated whether APA affected the oncogenic potential of UCK2 and MORC2 in KIRC. According to the NCBI database, a proximal PAS was found in both UCK2 or MORC2 (Supplemental Figure 3, A–C). Therefore, the short and long 3′UTR (full-length) UCK2 or MORC2 plasmids were constructed (Figure 2A) and then transfected in Caki-1 and A498 cells, respectively. In order to confirm equal efficiency of transfection, the pEGFP vector was cotransfected. Although transfection of long or short 3′UTR UCK2 led to similar protein production, short 3′UTR MORC2 produced more MORC2 protein than the long plasmid (Figure 2B), suggesting that MORC2 expression was regulated by APA. We then tested whether APA affected the oncogenic potential of UCK2 and MORC2. Consistent with the above finding, transfection of short or long 3′UTR UCK2 had similar influence on proliferation (Figure 2C) and clonogenicity of KIRC cells (Figure 2E and Supplemental Figure 3D). In contrast, the cells transfected with short 3′UTR MORC2 gained enhanced proliferation (Figure 2D) and clonogenicity (Figure 2, F and G, and Supplemental Figure 3, E and F), compared with those of the cells transfected with long 3′UTR MORC2. Furthermore, the superior oncogenic potential of short 3′UTR MORC2 was validated in vivo by a xenograft tumor formation experiment that showed accelerated tumor formation of short 3′UTR MORC2 stably expressed Caki-1 cells (Figure 2, H and I).

**Figure 3:**
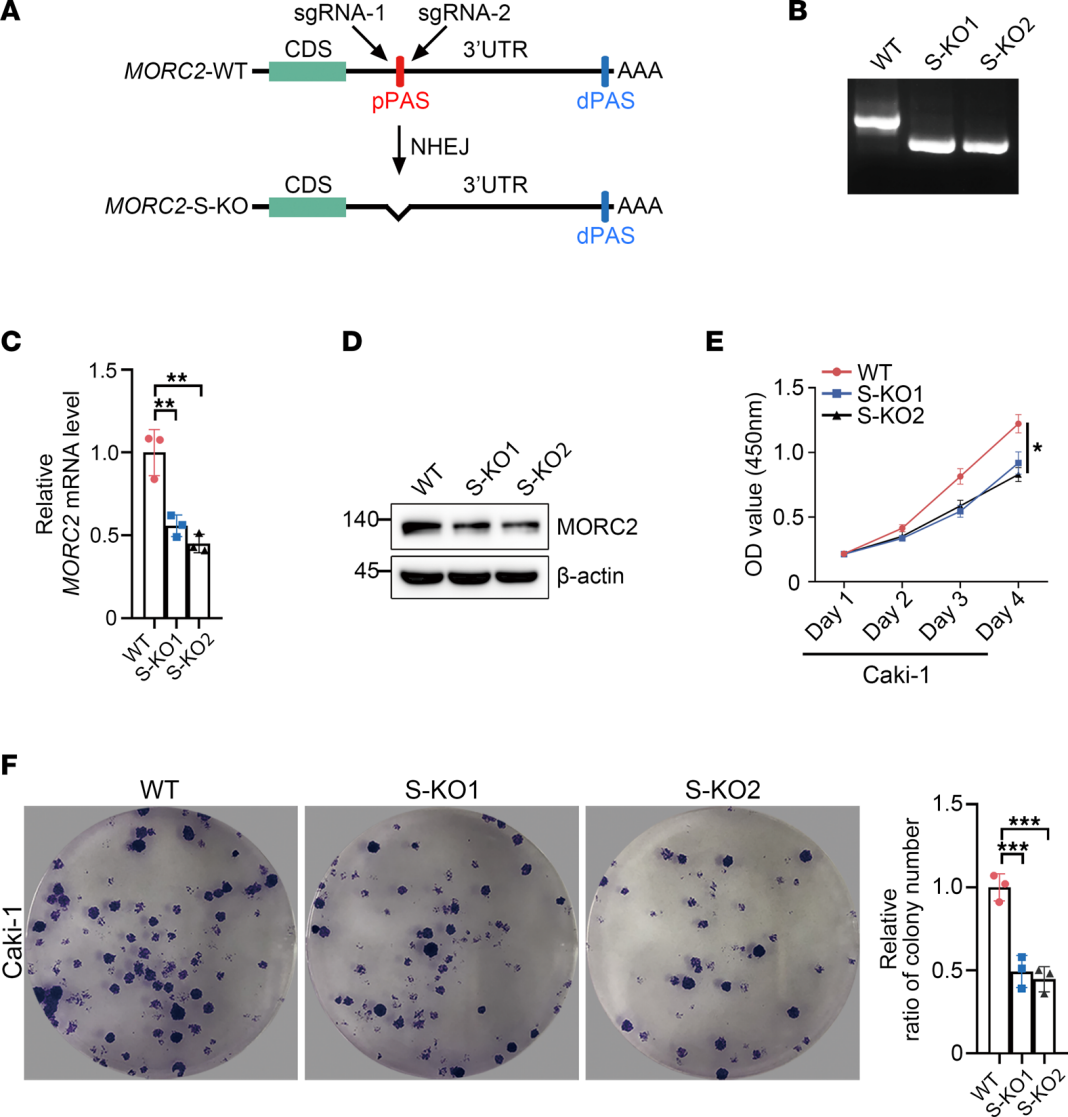
Deletion of short 3′UTR *MORC2* impairs the proliferation of KIRC cells. (**A**) Schematic diagram indicating the principle to construct MORC2-short KO (S-KO) Caki-1 cells. (**B**) PCR was performed to verify the construction of MORC2 S-KO Caki-1 cells. (**C**) qPCR was performed to evaluate the expression of MORC2 in WT and S-KO1/2 (clone nos. 1 and 2) Caki-1 cells (*n* = 3). (**D**) Immunoblotting was performed to evaluate the expression of MORC2 in WT and S-KO Caki-1 cells. (**E**) CCK8 assays were performed to evaluate the proliferation rate of WT and S-KO Caki-1 cells (*n* = 3). (**F**) Colony formation assays were performed and quantitatively analyzed to evaluate the clonogenicity of WT and S-KO Caki-1 cells (*n* = 3). All data represent the mean ± SD. One-way ANOVA with Tukey multiple-comparison test analyses were performed. *P < 0.05; **P < 0.01; ***P < 0.001.

To confirm the above observation, we deleted the proximal PAS of MORC2 in Caki-1 cells (Figure 3, A and B). Surprisingly, MORC2 expression was undermined in S-KO cells compared with that observed in WT Caki-1 cells (Figure 3, C and D). S-KO Caki-1 cells displayed inferior proliferation and clonogenicity (Figure 3, E and F), suggesting that 3′UTR shortening upgraded the oncogenic potential of MORC2 in KIRC by upregulating MORC2.

### 3′UTR shortening upregulates MORC2 expression by stabilizing MORC2 mRNA.

**Figure 4:**
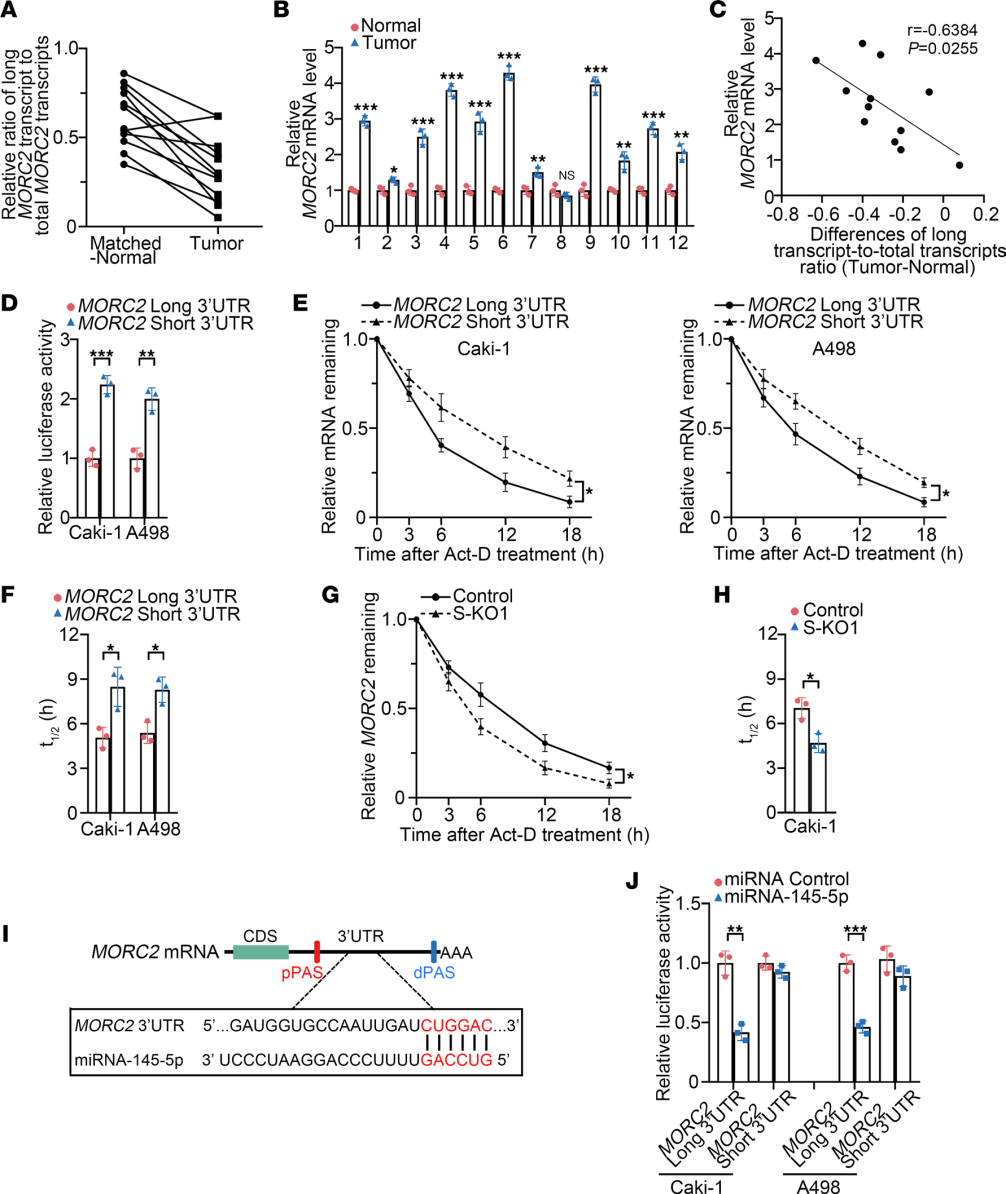
3′UTR shortening upregulates MORC2 expression by stabilizing *MORC2* mRNA. (**A**) qPCR was performed to evaluate the ratio of long 3′UTR MORC2 expression/total MORC2 expression in 12 pairs of KIRC tissues and matched normal tissues (*n* = 3). (**B**) qPCR was performed to evaluate expression of MORC2 in 12 pairs of KIRC tissues and matched normal tissues (*n* = 3). (**C**) The correlation between the extent of MORC2 3′UTR shortening and the extent of MORC2 upregulation in KIRC tissues was analyzed. (**D**) Luciferase reporter assay was performed to examine luciferase activity produced by plasmids carrying short or long 3′UTR of MORC2 (*n* = 3). (**E** and **F**) Isoform-specific qPCR was performed to evaluate (**E**) and quantitatively analyze (**F**) the half-life of long and short 3′UTR MORC2 in Caki-1 (left) and A498 (right) cells (*n* = 3). (**G** and **H**) qPCR was performed to evaluate (**G**) and quantitatively analyze (**H**) the half-life of MORC2 in WT and S-KO Caki-1 cells (*n* = 3). (**I**) Schematic diagram indicating the binding site of miR–145-5p at 3′UTR of MORC2. (**J**) Luciferase reporter assay was performed to evaluate the effect of miR–145-5p on short or long 3′UTR of MORC2 in Caki-1 and A498 cells (*n* = 3). All data represent the mean ± SD. Two-tailed *t* test were performed. *P < 0.05; **P < 0.01; ***P < 0.001.

To verify the above in vitro finding, we collected 12 pairs of matched KIRC and normal kidney tissues. As demonstrated by the ratio of long 3′UTR MORC2 transcript/total MORC2 transcripts, 3′UTR shortening of MORC2 was found in most KIRC tissues (Figure 4A). The mRNA level of MORC2 was also upregulated in the majority of KIRC tissues (Figure 4B). More importantly, the extent of MORC2 3′UTR shortening was associated positively with the extent of MORC2 upregulation in KIRC tissues (Figure 4C). This finding provided confirmation that 3′UTR shortening upregulates MORC2 in KIRC.

We next deciphered how 3′UTR shortening upregulated MORC2. First, reporter plasmids carrying short or long 3′UTR of MORC2 were constructed for luciferase reporter assays. We showed that the luciferase activity of MORC2 short 3′UTR was more than that of the long form (Figure 4D). We then applied isoform-specific quantitative PCR (qPCR) (20) to determine the mRNA stability of long or short 3′UTR MORC2. In both cells, short 3′UTR MORC2 showed a lower degradation rate than the long 3′UTR MORC2 (Figure 4, E and F), suggesting that 3′UTR shortening elevated the stability of MORC2 mRNA. On the other hand, the mRNA stability of MORC2 was weaker in S-KO Caki-1 cells (Figure 4, G and H), again supporting that 3′UTR shortening favored MORC2 expression by stabilizing MORC2 mRNA.

We further investigated how 3′UTR shortening stabilized MORC2. The microRNA that associates with the 3′UTR region of mRNA acts as a decisive role in mRNA stability; therefore, we suspected that 3′UTR shortening stabilized MORC2 by abrogating the miRNA-mRNA interaction. Previous studies identified miR–145-5p as a MORC2-targeted miRNA, with its binding site located downstream of proximal polyA site (pPAS) (21) (Figure 4I). There is also evidence that miR–145-5p has a tumor-suppressive effect in KIRC (22). To test whether 3′UTR shortening facilitated MORC2 mRNA evading from miR–145-5p, the miR–145-5p mimic was cotransfected with a luciferase reporter carrying either short or long 3′UTR of MORC2. We found that the miR–145-5p mimic decreased the luciferase activity of long MORC2 3′UTR (Figure 4J), whereas the luciferase activity of short MORC2 3′UTR was only mildly affected. This indicated that 3′UTR shortening stabilizes MORC2 mRNA by preventing miRNA-mediated degradation.

### MORC2 acts as an oncogene in KIRC mainly depending on downregulating tumor suppressor DAPK1 via DNA methylation.

**Figure 5:**
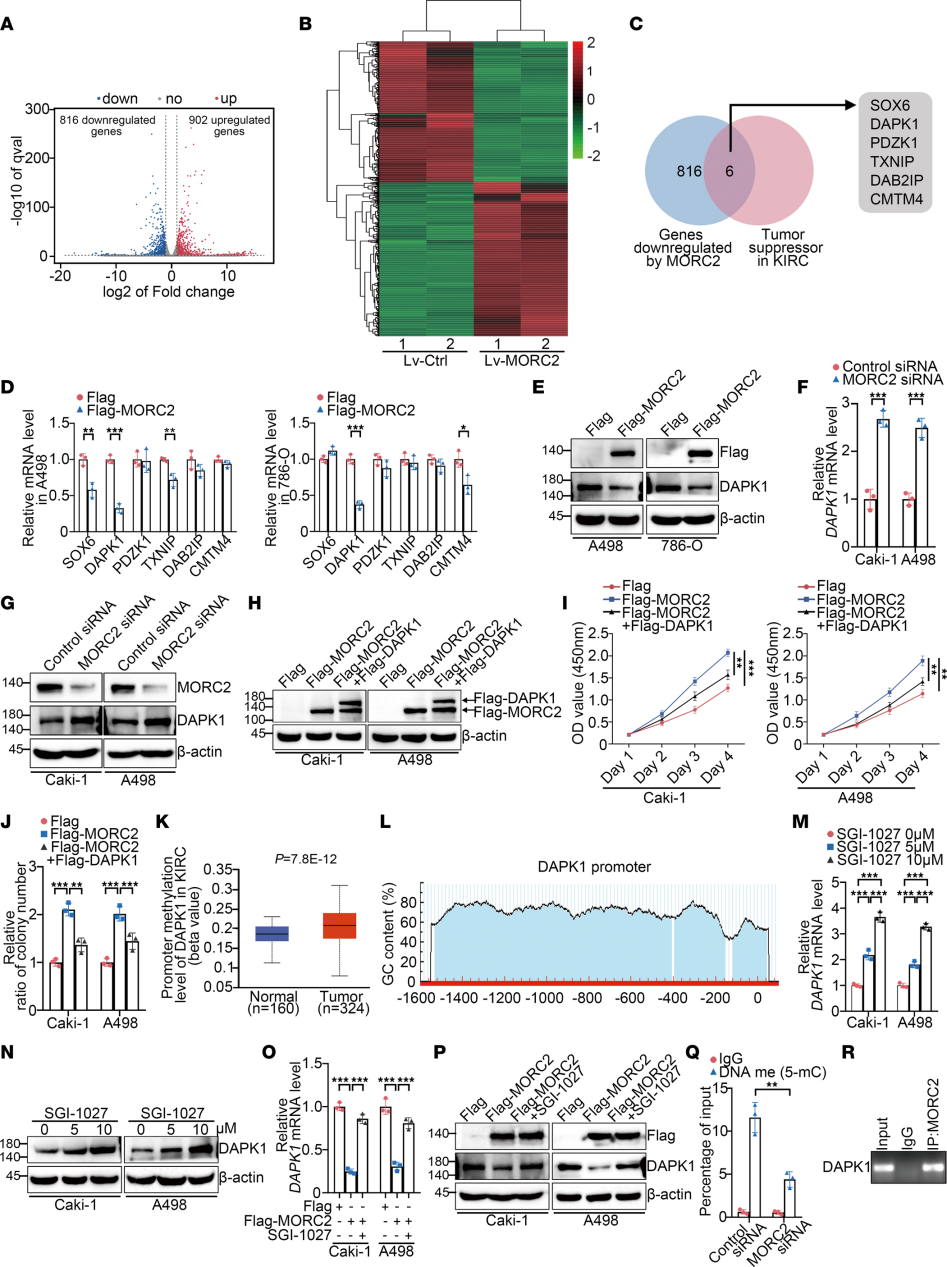
*MORC2* acts as an oncogene in KIRC mainly depending on downregulating tumor suppressor DAPK1 via DNA methylation. (**A** and **B**) Volcano plot (**A**) and heatmap (**B**) indicating the differentially expressed genes in control and MORC2 stably expressed Caki-1 cells (*n* = 2). (**C**) Venn diagram indicating the known KIRC suppressors among MORC2-regulated genes. (**D** and **E**) qPCR (**D**) and immunoblotting (**E**) were performed to evaluate expression of indicated genes in KIRC cells transfected with Flag or Flag-MORC2 plasmid (*n* = 3). (**F** and **G**) qPCR (**F**) and immunoblotting (**G**) were performed to evaluate DAPK1 expression in KIRC cells transfected with control or MORC2-specific siRNA (*n* = 3). (**H**) Immunoblotting was performed to evaluate expression of Flag-MORC2 and Flag-DAPK1 in KIRC cells with indicated treatments. (**I** and **J**) CCK8 (**I**) and colony formation (**J**) assays were performed to evaluate proliferation or clonogenicity of KIRC cells with indicated treatments (*n* = 3). (**K**) The promoter methylation of DAPK1 in KIRC and normal kidney was analyzed with UALCAN database. (**L**) Graph indicating the CpG island of DAPK1 from UROGENE database. (**M** and **N**) qPCR (**M**) and immunoblotting (**N**) were performed to evaluate DAPK1 expression in KIRC cells treated with SGI-1027 (*n* = 3). (**O** and **P**) qPCR (**O**) and immunoblotting (**P**) were performed to evaluate DAPK1 expression in KIRC cells with indicated treatments (*n* = 3). (**Q**) MeDIP-qPCR was performed to evaluate promoter methylation of DAPK1 in Caki-1 cells transfected with control or MORC2-specific siRNA (*n* = 3). (**R**) ChIP-PCR was performed to detect the MORC2-DAPK1 promoter binding in Caki-1 cells. All data represent the mean ± SD. Two-tailed *t* test, 1-way ANOVA with Tukey multiple-comparison test, or 2-way ANOVA with Tukey multiple-comparison test analyses were performed. **P < 0.01; ***P < 0.001.

To better reveal the prooncogenic APA/MORC2 axis in KIRC, we sought to identify the main downstream mediator of MORC2 in KIRC carcinogenesis. RNA-Seq of MORC2 stably expressed Caki-1 cells, and control Caki-1 cells disclosed various differentially expressed genes between these 2 groups (Figure 5, A and B, and Supplemental Data 2). Considering MORC2’s canonical function in epigenetic silencing (23, 24), we focused on the genes downregulated by MORC2 and then performed KEGG and gene ontology (GO) analyses (Supplemental Figure 4, A–D). Surprisingly, multiple previously reported tumor suppressors of KIRC were found in these downregulated genes (Figure 5C), including SOX6, DAPK1, PDZK1, TXNIP, DAB2IP, and CMTM4. We then examined whether these candidates were downregulated consistently by MORC2 in other KIRC cells. Among these genes, DAPK1 was shown to be significantly downregulated by MORC2 in both A498 and 786-O cells (Figure 5, D and E), suggesting DAPK1 as potential downstream target of MORC2 in KIRC. Conversely, MORC2 silencing upregulated DAPK1 (Figure 5, F and G). Next, we examined whether MORC2 functioned in KIRC depending on DAPK1 downregulation. To this end, Flag-DAPK1 was transfected in KIRC cells following Flag-MORC2 transfection (Figure 5H). We showed that the enhanced proliferation and clonogenicity of KIRC cells induced by Flag-MORC2 transfection was attenuated by Flag-DAPK1 transfection (Figure 5, I and J, and Supplemental Figure 4E), indicating that MORC2 functions as a KIRC oncogene mainly via downregulating DAPK1.

We continued to explore how MORC2 downregulated DAPK1 in KIRC. First, we wondered about the mechanism of DAPK1 downregulation in KIRC. Previous publications frequently reported that DNA hypermethylation of DAPK1 was found in patients with KIRC and was associated with a loss of DAPK1 and poor prognosis (25–28). We showed that the methylation level of the DAPK1 promoter was higher in KIRC (Figure 5K). In addition, an analysis of MethPrimer database (www.urogene.org) demonstrated that the DAPK1 promoter contained a typical CpG island (Figure 5L), again implying that DAPK1 is regulated by DNA methylation. Therefore, we treated KIRC cells with the DNA methyltransferase inhibitor SGI-1027 and found that DAPK1 was upregulated by SGI-1027 (Figure 5, M and N), indicating that DAPK1 is repressed by promoter methylation in KIRC. The above findings implied that MORC2 downregulates DAPK1 via DNA methylation. Therefore, after transfection with Flag or Flag-MORC2, KIRC cells was treated with SGI-1027. SGI-1027 significantly reversed MORC2-induced DAPK1 downregulation (Figure 5, O and P), indicating that MORC2 downregulated DAPK1 through DNA methylation. A methylated DNA IP (MeDIP) assay further demonstrated that the level of promoter methylation of DAPK1 was decreased by MORC2 silencing (Figure 5Q). In addition, endogenous MORC2 was confirmed to be associated with the DAPK1 promoter (Figure 5R), indicating that MORC2 participated in DAPK1 promoter methylation. Taken together, MORC2 acts as an oncogene in KIRC, mainly depending on DAPK1 repression via DNA methylation.

### 3′UTR shortening of MORC2 amplifies promoter methylation and downregulation of DAPK1 via enhancing DNMT3A recruitment.

**Figure 6:**
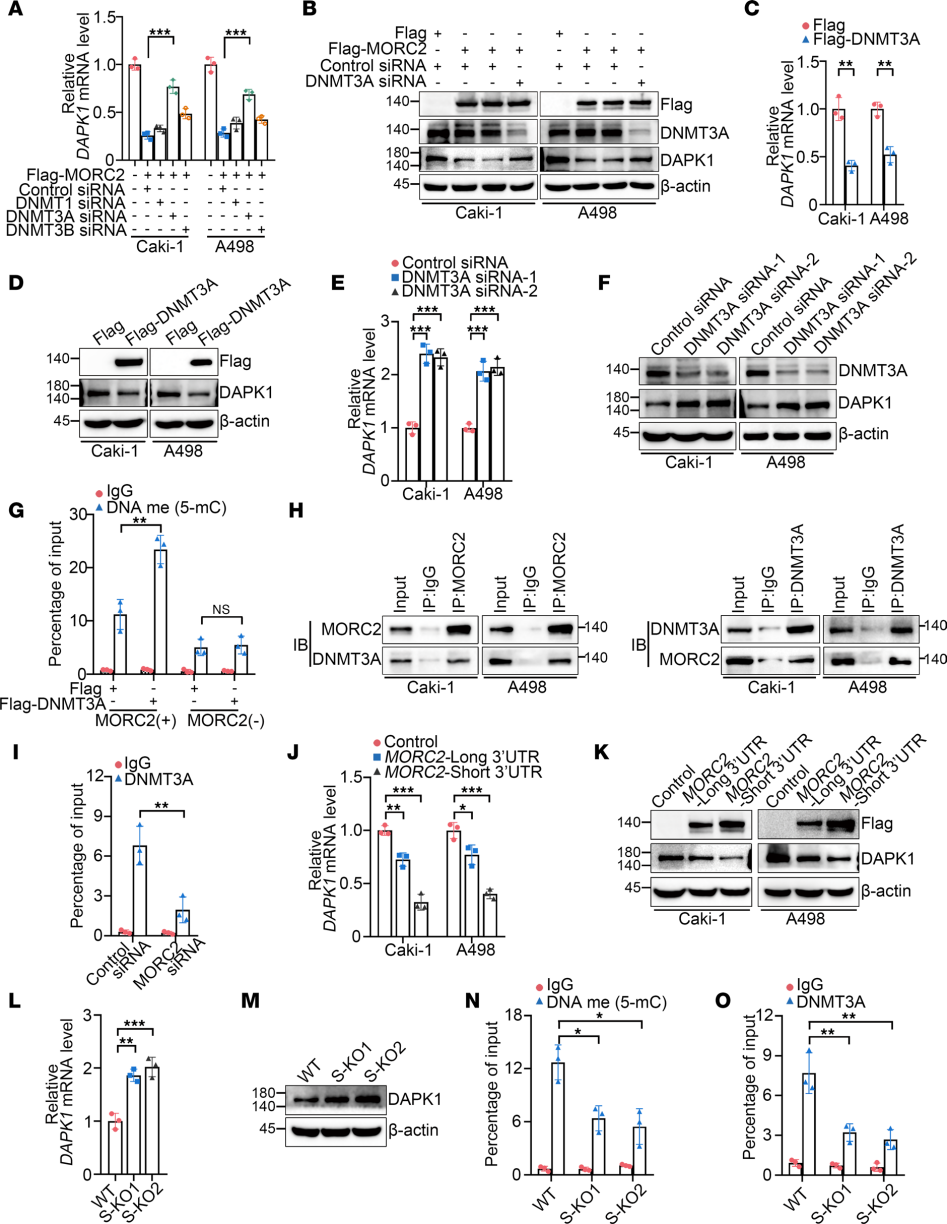
3′UTR shortening of MORC2 amplifies promoter methylation and downregulation of DAPK1 via enhancing DNMT3A recruitment. (**A** and **B**) qPCR (**A**) and immunoblotting (**B**) were performed to evaluate DAPK1 expression in KIRC cells transfected with Flag vector or Flag-MORC2 with silencing of indicated gene respectively (*n* = 3). (**C** and **D**) qPCR (**C**) and immunoblotting (**D**) were performed to evaluate DAPK1 expression in KIRC cells transfected with Flag vector or Flag-DNMT3A (*n* = 3). (**E** and **F**) qPCR (**E**) and immunoblotting (**F**) were performed to evaluate DAPK1 expression in KIRC cells transfected with control or DNMT3A-specific siRNA (*n* = 3). (**G**) MeDIP-qPCR was performed to evaluate promoter methylation level of DAPK1 in WT and MORC2-depleted Caki-1 cells transfected with Flag vector or Flag-DNMT3A (*n* = 3). (**H**) Co-IP was performed to detect the interaction between MORC2 and DNMT3A in KIRC cells. (**I**) ChIP-qPCR was performed to determine the abundance of DNMT3A at the DAPK1 promoter in Caki-1 cells transfected with control or MORC2-specific siRNA (*n* = 3). (**J** and **K**) qPCR (**J**) and immunoblotting (**K**) were performed to evaluate DAPK1 expression in KIRC cells transfected with vector, short 3′UTR, or long 3′UTR MORC2 plasmid (*n* = 3). (**L** and **M**) qPCR (**L**) and immunoblotting (**M**) were performed to evaluate DAPK1 expression in WT and S-KO Caki-1 cells (*n* = 3). (**N**) MeDIP-qPCR was performed to evaluate promoter methylation level of DAPK1 in WT and S-KO Caki-1 cells (*n* = 3). (**O**) ChIP-qPCR was performed to determine the abundance of DNMT3A at the DAPK1 promoter in WT and S-KO Caki-1 cells (*n* = 3). All data represent the mean ± SD. Two-tailed *t* test, 1-way ANOVA with Tukey multiple-comparison test, or 2-way ANOVA with Tukey multiple-comparison test analyses were performed. *P < 0.05; **P < 0.01; ***P < 0.001.

Given that MORC2 gained no DNA methyltransferase activity, we next investigated how MORC2 functioned in methylation of the DAPK1 promoter. We first determined which DNA methyltransferase mediated MORC2-induced DAPK1 downregulation and showed that DNMT3A silencing recovered DAPK1 expression in cells transfected with Flag-MORC2 (Figure 6, A and B). This result suggested that MORC2 depended on DNMT3A to repress DAPK1. We also confirmed that overexpression (Figure 6, C and D) or silencing (Figure 6, E and F) of DNMT3A alone downregulated or upregulated DAPK1, respectively. These results indicate DNMT3A as the direct catalyst of promoter hypermethylation of DAPK1 and provide further support to the possibility that MORC2 downregulated DAPK1 through DNMT3A. More importantly, Flag-DNMT3A transfection elevated methylation level of the DAPK1 promoter, which was markedly attenuated by MORC2 silencing (Figure 6G), implying that MORC2 facilitated DNMT3A to induce hypermethylation of the DAPK1 promoter. Moreover, the interaction between MORC2 and DNMT3A was confirmed in KIRC cells (Figure 6H), with MORC2 silencing impairing the association between DNMT3A and the DAPK1 promoter (Figure 6I). These data indicate that MORC2 interacts with DNMT3A and recruits DNMT3A to the DAPK1 promoter, thereby inducing promoter hypermethylation and downregulation of DAPK1.

We next examined whether 3′UTR shortening of MORC2 affected DAPK1 downregulation. First, we showed that short 3′UTR MORC2 induced significantly greater downregulation of DAPK1 (Figure 6, J and K). In contrast, DAPK1 expression was enhanced in S-KO Caki-1 cells (Figure 6, L and M). In addition, methylation of DAPK1 promoter (Figure 6N) and DNMT3A recruitment to the DAPK1 promoter (Figure 6O) were both suppressed in S-KO cells, indicating that 3′UTR shortening of MORC2 amplified DAPK1 downregulation via strengthening DNMT3A recruitment and methylation of the DAPK1 promoter. These data therefore illustrate an APA/MORC2/DAPK1 axis in KIRC.

### Loss of APA regulator NUDT21 induces 3′UTR shortening and upregulation of MORC2 in KIRC.

**Figure 7:**
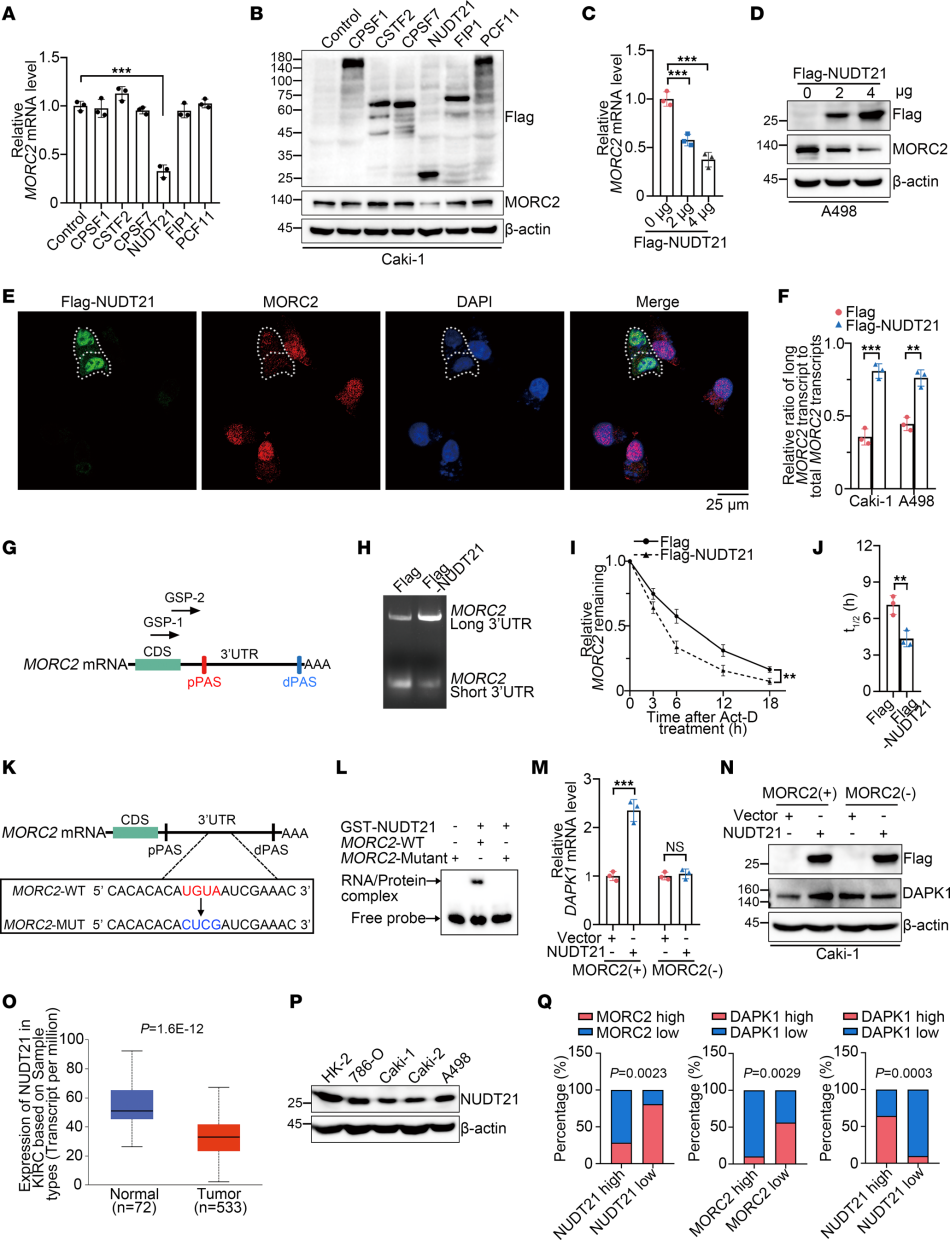
Loss of APA regulator NUDT21 induces 3′UTR shortening and upregulation of *MORC2* in KIRC. (**A** and **B**) qPCR (**A**) and immunoblots (**B**) were performed to evaluate MORC2 expression in Caki-1 cells transfected with indicated plasmid (*n* = 3). (**C** and **D**) qPCR (**C**) and immunoblots (**D**) were performed to evaluate MORC2 expression in A498 cells transfected with Flag vector or Flag-NUDT21 plasmid (*n* = 3). (**E**) Immunofluorescence staining was performed to evaluate MORC2 expression in Caki-1 cells transfected with Flag-NUDT21 plasmid. Scale bar: 25 μm. (**F**) qPCR was performed to evaluate the ratio of long 3′UTR MORC2 expression/total MORC2 expression in KIRC cells transfected with Flag vector or Flag-NUDT21 plasmid (*n* = 3). (**G** and **H**) 3′RACE was performed with GSP-1/2 primers (**G**) to evaluate 3′UTR of MORC2 in Caki-1 cells transfected with Flag vector or Flag-NUDT21 plasmid. (**I** and **J**) qPCR was performed to quantitatively analyze half-life of MORC2 in Caki-1 cells transfected with Flag vector or Flag-NUDT21 plasmid (*n* = 3). (**K**) Diagram indicated the UGUA motif of MORC2 and the mutant motif. (**L**) EMSA was performed to explore the binding site of NUDT21 at MORC2 3′UTR. (**M** and **N**) qPCR (**M**) and immunoblots (**N**) were performed to evaluate DAPK1 expression in WT and MORC2-depleted Caki-1 cells transfected with Flag vector or Flag-NUDT21 plasmid (*n* = 3). (**O**) NUDT21 expression in KIRC tissues and normal kidney tissues was analyzed with UALCAN database. (**P**) Immunoblotting was performed to evaluate NUDT21 expression in HK-2 cells and KIRC cells. (**Q**) The expression associations between NUDT21 and MORC2/DAPK1, and between MORC2 and DAPK1, were analyzed in KIRC specimens. All data represent the mean ± SD. Two-tailed *t* test analyses were performed. **P < 0.01; ***P < 0.001.

We next investigated why 3′UTR shortening of MORC2 occurred in KIRC. Generally, APA is regulated by a variety of APA regulators, with the majority of APA reprogramming in cancer due to their dysregulation (29). To identify the main APA regulator of MORC2, 6 fundamental APA regulators were respectively transfected in Caki-1 cells. We showed that Flag-NUDT21 transfection significantly downregulated MORC2 (Figure 7, A and B). Such MORC2 downregulation by NUDT21 was also validated in A498 cells (Figure 7, C and D). Additionally, immunofluorescence staining was performed in Caki-1 cells, demonstrating that MORC2 expression was impaired in NUDT21-transfected cells (Figure 7E). More importantly, Flag-NUDT21 transfection was shown to promote 3′UTR lengthening of MORC2 (Figure 7F), and this evidence suggests that NUDT21 suppressed MORC2 expression by favoring long 3′UTR MORC2. These findings gained more support from a rapid amplification of 3′cDNA ends (3′RACE) assay (Figure 7, G and H). We then showed a consistent result that Flag-NUDT21 transfection destabilized MORC2 mRNA (Figure 7, I and J). We next specified the binding position of NUDT21 at MORC2 3′UTR. We learned from previous publications that NUDT21 binds with UGUA element upstream of PAS and found an UGUA motif upstream of MORC2 distal PAS (dPAS) (Figure 7K). We then examined whether it was the binding site of NUDT21. The RNA electrophoretic mobility shift assay (EMSA) demonstrated that this UGUA motif was indispensable for NUDT21-MORC2 binding (Figure 7L), supporting NUDT21 as the APA regulator of MORC2. We also explored whether NUDT21 regulated DAPK1 through MORC2 and found that NUDT21 upregulated DAPK1 in WT Caki-1 cells. Nevertheless, only minor effects were observed in MORC2-depleted cells (Figure 7, M and N). These data indicate that NUDT21 favored dPAS utilization of MORC2 to suppress MORC2 expression in KIRC, suggesting a NUDT21/APA/MORC2/DAPK1 axis in KIRC.

According to the UALCAN database (ualcan.path.uab.edu/analysis.html), NUDT21 was downregulated in KIRC (Figure 7O). NUDT21 was also shown downregulated in KIRC cells compared with human proximal tubular HK-2 cells (Figure 7P). Additionally, NUDT21 expression displayed a negative association with tumor grade (Supplemental Figure 5A) and a positive association with KIRC prognosis (Supplemental Figure 5B). To confirm the NUDT21/MORC2/DAPK1 axis in KIRC, the clinical significance of this axis was examined (30, 31). We collected 45 clinical specimens of KIRC and detected the expression of NUDT21, MORC2, and DAPK1 using IHC staining (Supplemental Figure 5C). As shown in Figure 7Q, a negative association was observed between NUDT21 and MORC2 or between MORC2 and DAPK1. Meanwhile, we confirmed that NUDT21 expression was positively associated with DAPK1 expression. Taken together, these data suggest that the loss of NUDT21 is responsible for 3′UTR shortening and upregulation of MORC2, indicating a NUDT21/MORC2/DAPK1 axis in KIRC.

### DNMT3B-induced promoter methylation downregulates NUDT21 in KIRC.

**Figure 8:**
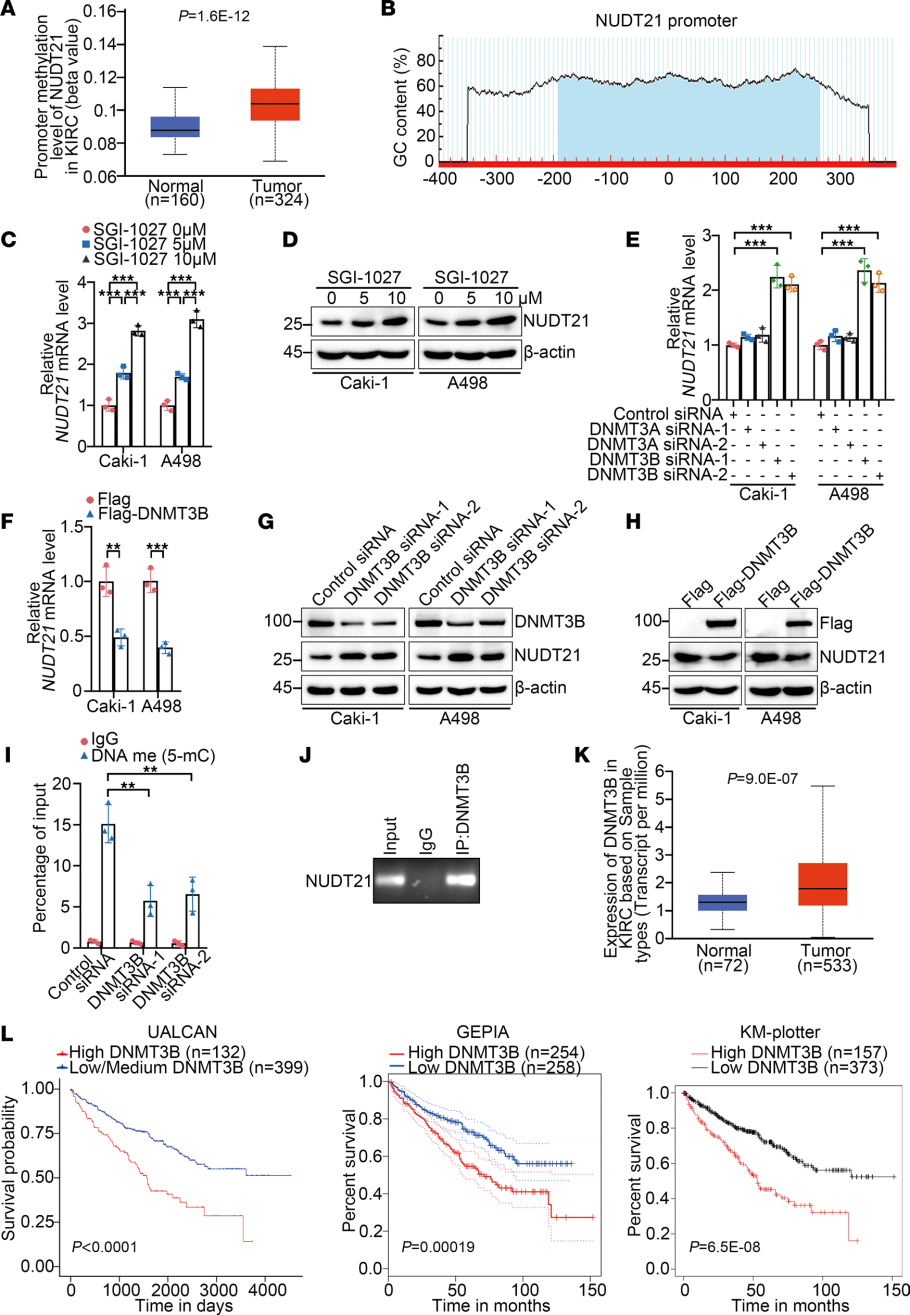
DNMT3B-induced promoter methylation downregulates NUDT21 in KIRC. (**A**) The promoter methylation levels of DAPK1 in KIRC tissues and matched normal kidney tissues were analyzed with the UALCAN database. (**B**) Graph indicating the position of the CpG island at the DAPK1 promoter. (**C**) qPCR was performed to evaluate expression of DAPK1 in Caki-1 and A498 cells treated with SGI-1027 (*n* = 3). (**D**) Immunoblotting was performed to evaluate expression of DAPK1 in Caki-1 and A498 cells treated with SGI-1027. (**E**) qPCR was performed to evaluate expression of DAPK1 in Caki-1 and A498 cells transfected with control, DNMT3A-specific, or DNMT3B-specific siRNAs (*n* = 3). (**F**) qPCR was performed to evaluate expression of DAPK1 in Caki-1 and A498 cells transfected with empty Flag vector or Flag-DNMT3B (*n* = 3). (**G**) Immunoblotting was performed to evaluate expression of DAPK1 in Caki-1 and A498 cells transfected with control siRNA or DNMT3B-specific siRNAs. (**H**) Immunoblotting was performed to evaluate expression of DAPK1 in Caki-1 and A498 cells transfected with empty Flag vector or Flag-DNMT3B. (**I**) MeDIP-qPCR was performed to evaluate promoter methylation level of DAPK1 in Caki-1 cells transfected with empty Flag vector or Flag-DNMT3B (*n* = 3). (**J**) ChIP-PCR was performed to detect the binding of DNMT3B to promoter of DAPK1 in Caki-1 cells (*n* = 3). (**K**) The mRNA levels of DNMT3B in KIRC tissues and matched normal kidney tissues were analyzed with UALCAN database. (**L**) Prognosis association of DNMT3B in KIRC was analyzed with UALCAN (left), GEPIA (middle), and KM plotter (right) Databases. All data represent the mean ± SD. Two-tailed *t* test, 1-way ANOVA with Tukey multiple-comparison test, or 2-way ANOVA with Tukey multiple-comparison test analyses were performed. **P < 0.01; ***P < 0.001.

Given that NUDT21 loss represented a molecular origin of APA reprogramming of MORC2, we wondered how NUDT21 was lost in KIRC. The inferior mRNA level of NUDT21 in KIRC suggested loss of NUDT21 derived from impaired transcription. Surprisingly, the UALCAN database showed that methylation level of NUDT21 promoter was elevated in KIRC (Figure 8A). Meanwhile, a typical CpG island was also found in the NUDT21 promoter (Figure 8B), implying that NUDT21 loss was possibly due to DNA methylation. This hypothesis was supported by NUDT21 upregulation in KIRC cells treated with SGI-1027 (Figure 8, C and D). Next, we investigated which de novo DNA methyltransferase induced NUDT21 promoter hypermethylation. DNMT3B silencing fundamentally upregulated NUDT21 (Figure 8, E and G). Conversely, Flag-DNMT3B transfection downregulated NUDT21 (Figure 8, F and H), suggesting that DNMT3B was responsible for induction of hypermethylation of the NUDT21 promoter. Next, DNMT3B was found to enhance the methylation level of the NUDT21 promoter (Figure 8I) and to be associated with the NUDT21 promoter (Figure 8J), confirming that DNMT3B induced hypermethylation of the NUDT21 promoter in KIRC. Additionally, according to the UALCAN, GEPIA, and KM-plotter databases, DNMT3B expression was elevated and negatively associated with prognosis in KIRC (Figure 8, K and L). These findings suggest that DNTM3B upregulation in KIRC elevated the methylation level of the NUDT21 promoter, leading to the loss of NUDT21.

### NUDT21 functions as a tumor suppressor in KIRC, mainly depending on MORC2 downregulation.

**Figure 9:**
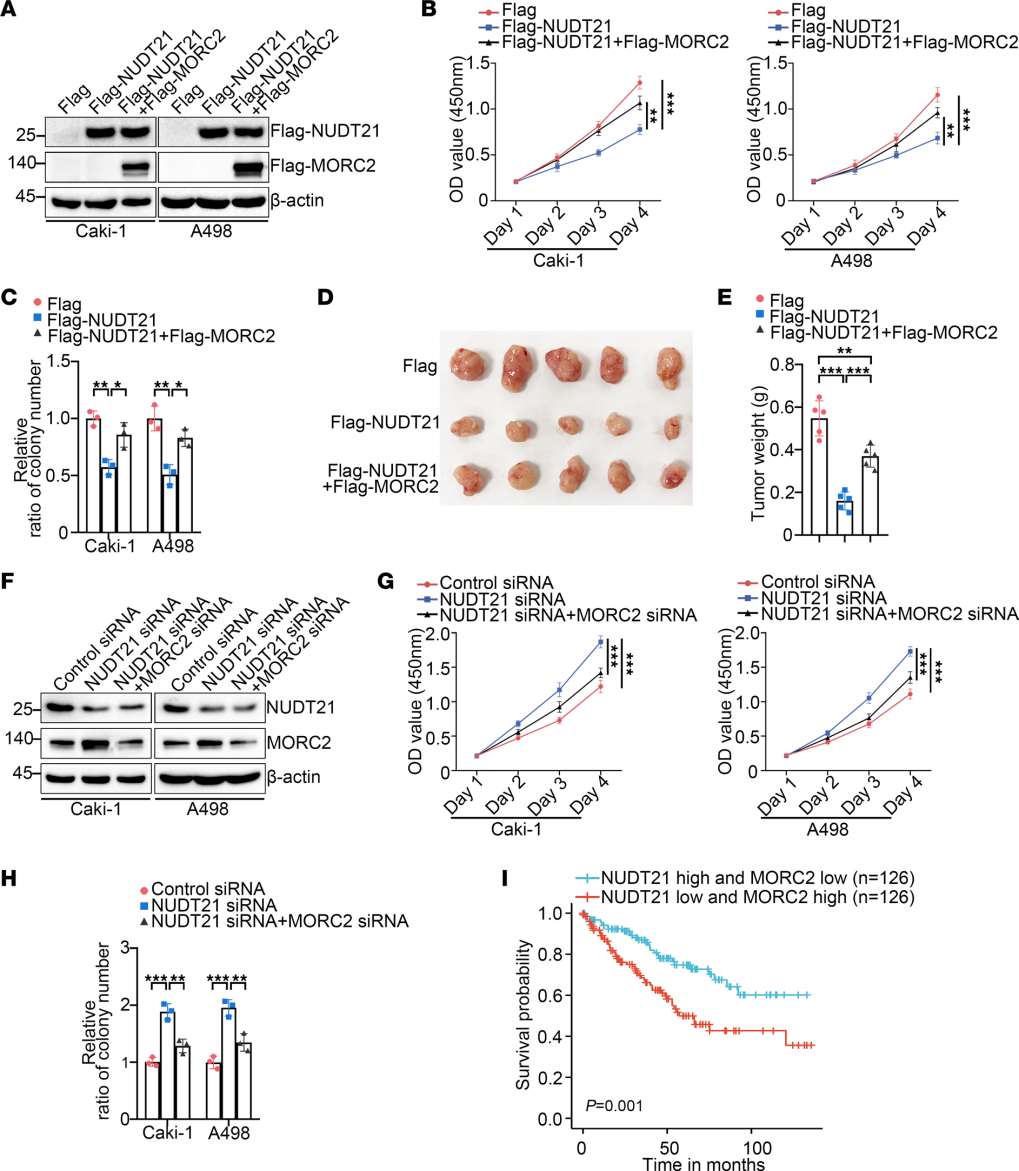
NUDT21 functions as a tumor suppressor mainly depending on MORC2 downregulation. (**A**) Immunoblotting was performed to evaluate the expression of Flag-NUDT21 and Flag-MORC2 in Caki-1 and A498 cells transfected with empty Flag vector or Flag-NUDT21 plasmid with or without MORC2 recovery. (**B**) CCK8 assays were performed to evaluate the proliferation rate of Caki-1 and A498 cells transfected with empty Flag vector or Flag-NUDT21 with or without MORC2 recovery (*n* = 3). (**C**) Colony formation assays were performed and quantitatively analyzed to evaluate the clonogenicity of Caki-1 and A498 cells transfected with empty Flag vector or Flag-NUDT21 with or without MORC2 recovery (*n* = 3). (**D** and **E**) In vivo xenograft tumor formation experiment was performed (**D**) and quantitatively analyzed (**E**) with control Caki-1 and NUDT21-stably expressed Caki-1 cells with or without MORC2 recovery (*n* = 5 per group). (**F**) Immunoblotting was performed to evaluate expression of NUDT21 and MORC2 in Caki-1 and A498 cells transfected with control siRNA or NUDT21-specific siRNA with or without MORC2 silencing. (**G**) CCK8 assays were performed to evaluate the proliferation rate of Caki-1 and A498 cells transfected with control siRNA or NUDT21-specific siRNA with or without MORC2 silencing (*n* = 3). (**H**) Colony formation assays were performed and quantitatively analyzed to evaluate the clonogenicity of Caki-1 and A498 cells transfected with control siRNA or NUDT21-specific siRNA with or without MORC2 silencing (*n* = 3). (**I**) Prognosis of patients with KIRC with high NUDT21 expression and low MORC2 expression or low NUDT21 expression and high MORC2 expression was analyzed with Xiantao database (www.xiantaozi.com) depending on TCGA data. All data represent the mean ± SD. Two-tailed *t* test analyses were performed. *P < 0.05; **P < 0.01; ***P < 0.001.

The above data suggest a potential tumor-suppressive role of NUDT21 in KIRC. To confirm this possibility, we transfected Flag-NUDT21 in KIRC cells and showed that NUDT21 effectively inhibited proliferation (Figure 9, A and B) and clonogenicity (Figure 9, A and C, and Supplemental Figure 6A) of KIRC cells. However, these effects of NUDT21 were attenuated by MORC2 recovery (Figure 9, B and C, and Supplemental Figure 6A). Moreover, as demonstrated by the xenograft tumor formation experiment, NUDT21 inhibited tumor formation of Caki-1 cells in vivo, which was reversed by MORC2 recovery (Figure 9, D and E). These data imply that NUDT21 functions as a tumor suppressor mainly depending on MORC2 downregulation. Conversely, NUDT21 silencing stimulated proliferation (Figure 9, F and G) and clonogenicity (Figure 9, F and H, and Supplemental Figure 6B) of KIRC cells, and this stimulation was relieved following MORC2 silencing, again supporting that NUDT21 acts as a tumor suppressor in KIRC through downregulating MORC2. Furthermore, based on TCGA data, we found that the patients with KIRC with both upregulated NUDT21 expression and downregulated MORC2 expression gained a considerably better prognosis than those with downregulated NUDT21 expression and upregulated MORC2 expression (Figure 9I).

### ASO, which enhances NUDT21 expression, inhibits proliferation and tumor formation of KIRC cells.

**Figure 10:**
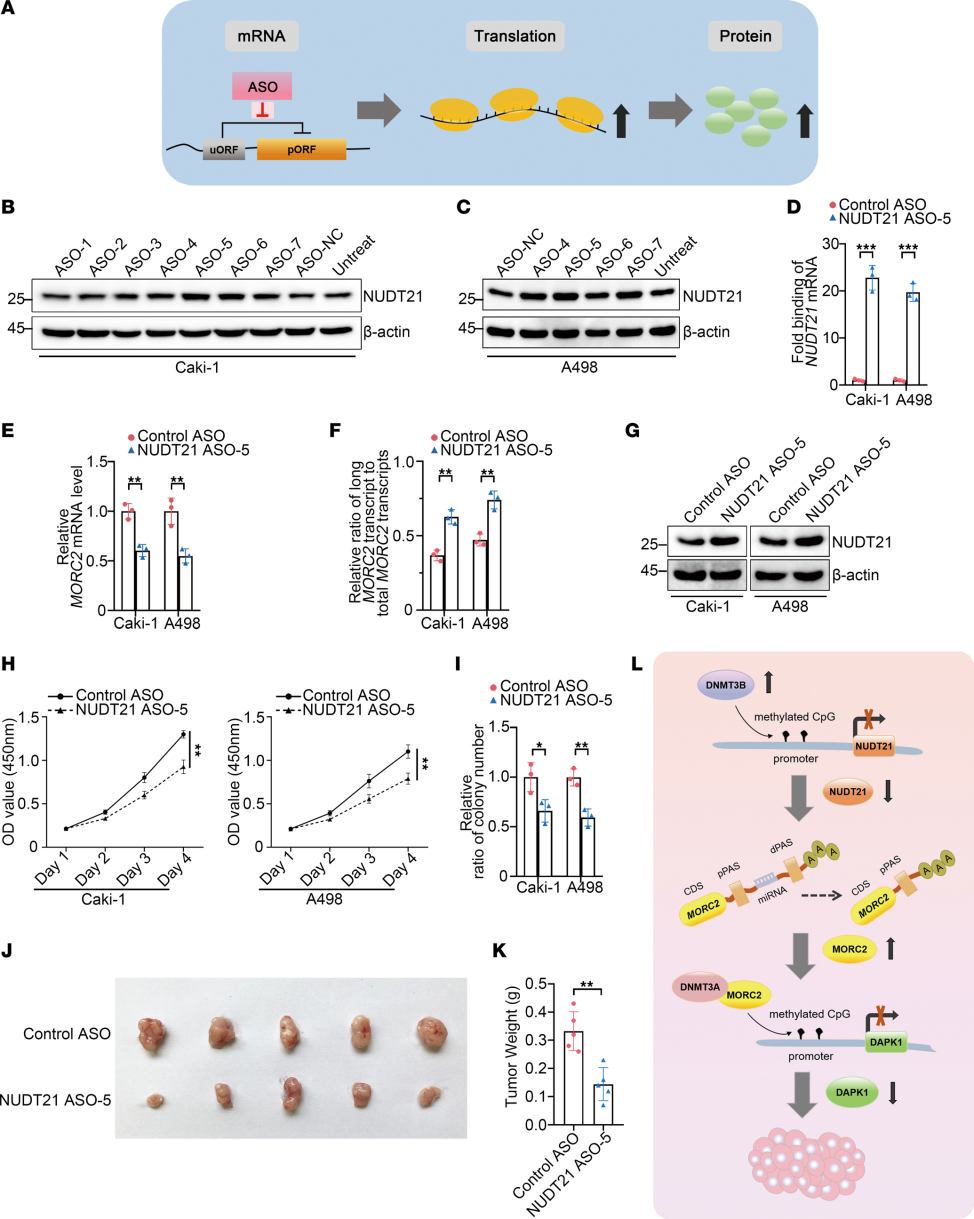
ASO-enhancing NUDT21 expression inhibits proliferation and tumor formation of KIRC cells. (**A**) Schematic diagram indicating the mechanism for ASO in upregulating targeted gene. (**B** and **C**) Immunoblotting was performed to evaluate NUDT21 expression in Caki-1 cells (**B**) and A498 cells (**C**) transfected with indicated ASOs. (**D**) RNA pull-down assay was performed to detect the binding of ASO-5 to the uORF of NUDT21. (**E**) qPCR was performed to evaluate expression of MORC2 in Caki-1 and A498 cells transfected with control ASO or ASO-5 (*n* = 3). (**F**) qPCR was performed to evaluate ratio of long 3′UTR MORC2 expression/total MORC2 expression in Caki-1 and A498 cells transfected with control ASO or ASO-5 (*n* = 3). (**G**) Immunoblotting was performed to evaluate expression of NUDT21 in Caki-1 and A498 cells transfected with control ASO or ASO-5. (**H**) CCK8 assays were performed to evaluate the proliferation rate of Caki-1 and A498 cells transfected with control ASO or ASO-5 (*n* = 3). (**I**) Colony formation assays were performed and quantitatively analyzed to evaluate the clonogenicity of Caki-1 and A498 cells transfected with control ASO or ASO-5 (*n* = 3). (**J** and **K**) In vivo xenograft tumor formation experiment was performed (**J**) and quantitatively analyzed (**K**) with Caki-1 cells transfected with control ASO or ASO-5 (*n* = 5 per group). (**L**) Schematic diagram indicating the mechanism that NUDT21 loss, induced by promoter methylation, reprogrammed APA of MORC2 and further epigenetically downregulated DAPK1, thereby leading to KIRC carcinogenesis. All data represent the mean ± SD. Two-tailed *t* test analyses were performed. *P < 0.05; **P < 0.01; ***P < 0.001.

The above data indicate that NUDT21 is a key tumor suppressor in KIRC, and therefore, we continued to develop a feasible therapy that would account for the enhancement of NUDT21 in KIRC. Elevating the level of therapeutic protein has been a challenge. However, ASO, one of the emerging RNA therapies, may be a promising option. In the process of translation, the occurrence of an upstream open reading frame (uORF) may reduce the efficiency of translation (32, 33). Thus, disrupting the uORF may enhance translation efficiency and protein expression of a target gene (Figure 10A). Based on this strategy, we attempted to enhance NUDT21 expression with ASO in KIRC. Seven ASO candidates, namely ASO-1 to ASO-7, were designed (Supplemental Figure 7, A and B), and the effectiveness in enhancing NUDT21 expression was examined by immunoblot. In Caki-1 cells, ASO-5 and ASO-6 were found to be effective (Figure 10B). Further evaluation in A498 cells indicated that ASO-5 achieved a better effect in enhancing NUDT21 (Figure 10C). Moreover, the RNA pull-down assay indicated that the ASO-5 bound to the uORF of NUDT21 in both cells (Figure 10D). Hence, ASO-5 was selected for further study. We showed that ASO-5 effectively downregulated MORC2 expression (Figure 10E) and elevated the ratio of long 3′UTR MORC2 (Figure 10F) in both KIRC cells, again validating the effect of ASO-5 in NUDT21 enhancement. More importantly, ASO-5 displayed significant effects in suppressing proliferation and clonogenicity of KIRC cells in vitro (Figure 10, G–I, and Supplemental Figure 7C) and in inhibiting tumor formation in vivo (Figure 10, J and K). These results suggest that enhancing NUDT21 with ASO is a promising therapeutic strategy for KIRC.

## Discussion

APA dysregulation has been identified as an emerging origin of carcinogenesis in many types of cancer, offering a potentially novel mechanistic explanation and a promising strategy of early diagnostic and therapy for cancer. However, in KIRC, although abundant APA reprogramming has been observed, whether or not and how APA may function has remained unknown. In view of this situation, we started this study to analyze the KIRC-specific APA changes and to explore the role of APA in KIRC. First, the genes that experience APA reprogramming in KIRC were analyzed. With a bioinformatic screen and further in vitro validation, UCK2 and MORC2 were identified as oncogenes for KIRC. Furthermore, only 3′UTR shortening of MORC2 was found to promote KIRC carcinogenesis by stabilizing MORC2 mRNA, indicating that MORC2 was the downstream effector of APA in KIRC. Mechanistically, MORC2 recruited DNMT3A to induce promoter hypermethylation of the tumor suppressor DAPK1, thereby promoting KIRC carcinogenesis. 3′UTR shortening strengthened this epigenetic silencing of DAPK1. In addition, loss of the APA regulator NUDT21, derived from DNMT3B-mediated promoter hypermethylation, was identified as the molecular origin of 3′UTR shortening of MORC2 in KIRC. We consistently validated that NUDT21 suppressed KIRC carcinogenesis mainly by downregulating MORC2. Finally, an ASO was designed to enhance NUDT21 expression and was validated to be effective in suppressing proliferation and clonogenicity of KIRC cells. In summary, this study first confirms and explains the function of APA in KIRC and elucidates the DNMT3B/NUDT21/APA/MORC2/DAPK1 axis in KIRC (Figure 10L).

This study first identified the role of APA in KIRC. Among the abundant APA reprogramming in KIRC, we identified 3′UTR shortening of MORC2-promoted KIRC carcinogenesis. This finding offered a mechanistic explanation to KIRC carcinogenesis and a promising therapeutic strategy. Although we found that 3′UTR shortening favored MORC2 expression, which is associated with poor prognosis of KIRC, due to the challenge in collecting KIRC tissues and matched normal tissues with prognosis data, we were unable to validate whether 3′UTR shortening of MORC2 could be used as a novel prognosis marker. Considering that 3′UTR shortening is an upstream event of oncogene overactivation, in the future, our focus will be whether an APA-based strategy could be used for early diagnosis for KIRC. Of note, it’s likely that our findings may be the first of many regarding APA’s function in KIRC. For example, we recently found that multiple genes, such as PDGFR, experienced 3′UTR shortening in drug-resistant KIRC cells. We will therefore continue to investigate the function of APA in KIRC, especially related to drug resistance.

Our study also demonstrated that the loss of NUDT21 was the origin of MORC2 3′UTR shortening in KIRC. Generally, prooncogenic 3′UTR shortening is induced by upregulation of APA regulators favoring utilization of pPAS or downregulation of those favoring utilization of dPAS. In KIRC, according to the online database, the pPAS-favoring regulators such as CSTF2, FIP1, and PCF11 displayed similar or even inferior expression, implying that NUDT21, the most important dPAS-favoring APA regulator, is the determinant regulator of APA in KIRC. Therefore, we concluded that NUDT21 loss was likely to be the major origin of prooncogenic APA reprogramming in KIRC. Although NUDT21 was previously found as a tumor suppressor in some types of cancer, no NUDT21-favoring therapy has been developed, possibly due to the challenge in elevating the level of therapeutic protein. On the other hand, ASO, one of the most promising mRNA therapies, has a naturally preferential distribution to the kidney, making it especially suitable for treatment of kidney disease. Given that, we applied ASO to enhanced NUDT21 expression and showed that it was effective in vitro and in vivo, thereby indicating this strategy as a promising one for KIRC.

The crosstalk among various epigenetic regulation is a major focus in epigenetic research. In terms of APA, previous studies identified that DNA methylation favored pPAS usage by enabling CTCF binding and recruitment of cohesin complex recruitment (15), making a difference in APA regulation. Herein, we showed that DNA methylation downregulated NUDT21, the most vital APA regulator favoring dPAS usage, uncovering a mechanism for DNA methylation in promoting pPAS usage. This study also identified that APA, in turn, impacted DNA methylation via regulating epigenetic modifier MORC2. We found various DNA methylation regulator gains at least 1 pPAS, suggesting that the role of APA in DNA methylation is currently underestimated. Collectively, this study clearly demonstrated a crosstalk between DNA methylation and APA in promoting carcinogenesis. We confirmed such crosstalk in other types of cancer cells and normal kidney tubular cells (our unpublished data), suggesting that this crosstalk may also function in other physiological and pathological processes.

MORC2, a newly identified chromatin modifier, has been identified as a vital participator in some types of cancer, especially in breast cancer (34) and liver cancer (23). In this study, we defined MORC2 as a key oncogene in KIRC. Moreover, MORC2 was also shown to function by epigenetically downregulating the tumor suppressor DAPK1, disclosing a prooncogenic pathway for MORC2. More importantly, MORC2 was upregulated in multiple types of cancer (35), with posttranslational modification previously regarded as being responsible for MORC2 upregulation (34). This study is the first to our knowledge to show that MORC2 is overactivated posttranscriptionally, offering a potential strategy for MORC2-targeted therapy. It should be noted that such APA-mediated MORC2 upregulation might not be limited to KIRC. For example, 3′UTR shortening of MORC2 is also reported in lung adenocarcinoma in which MORC2 mRNA level is elevated (16). In addition to APA, MORC2 is also potentially regulated by alternative splicing (AS). According to the TCGA SpliceSeq database (bioinformatics.mdanderson.org/TCGASpliceSeq), the percent spliced-in (PSI) of MORC2 in KIRC seems higher than that in normal kidney (Supplemental Figure 8), suggesting that MORC2 is regulated by AS in KIRC. AS functions by impacting protein diversity; thus, MORC2 upregulation in KIRC is not likely to be induced by AS. Nevertheless, the potential role of AS of MORC2 deserves further investigation. We also wondered whether DNA methylation regulates MORC2 expression in KIRC. We thus collected 18 pairs of KIRC tissues and matched normal kidney tissues, and we found no significant difference in promoter methylation levels of MORC2 between KIRC and normal kidney (Supplemental Figure 9), indicating DNA methylation at least is not the main contributor to MORC2 upregulation in KIRC.

DAPK1, a tumor suppressor of KIRC, is frequent lost in various types of cancer due to promoter hypermethylation. In KIRC, promoter hypermethylation of DAPK1 led to DAPK1 loss, progressive phenotype, and poor prognosis. Nevertheless, how the DAPK1 promoter was hypermethylated remained elusive. Even some controversial observations were found in previous studies. Herein, we confirmed DNMT3A was the DNA methyltransferase inducing DAPK1 promoter hypermethylation. More importantly, we found that the DNMT3A recruitment to the DAPK1 promoter required the mediation of MORC2. Therefore, the MORC2/DNMT3A/DAPK1 regulatory axis was uncovered.

## Methods

### Cell culture.

KIRC cells Caki-1, A498, and 786-O were offered by W.L. Xu (Sun Yat-sen University, Guangzhou, China). Human proximal tubular epithelial cell HK-2 and human embryonic kidney cell 293T were offered by L.T. Chen (Sun Yat-sen University, Guangzhou, China). HK-2 cells were cultured in DMEM/F-12 medium (Thermo Fisher Scientific) containing 10% FBS. 293T, A498, and 786-O cells were cultured in DMEM medium (Thermo Fisher Scientific) containing 10% FBS. Caki-1 cells were cultured in McCOY’s 5A medium (VivaCell) containing 10% FBS. All cells were incubated at 37°C in a humidified atmosphere with 5% CO_2_. In total, 10μM SGI-1027 (MCE) was used to treat cells.

### CCK8, colony formation, and soft agar assays.

For CCK8 assay, cells were seeded in a 96-well plate (2 × 10^3^ cells per well) and counted for 4 days. A CCK-8 cell counting kit (Vazyme) was used for determination of cell proliferation. For colony formation assay, cells were seeded in a 6-well plate (5 × 10^2^ cells per well) and cultured for 2 weeks. Cells were then fixed with 4% PFA and stained with 0.1% crystal violet. For soft agar assay, a mixture of 1.2% soft-agar with 2 × DMEM containing 10% FBS at a ratio of 50:50 was added into a 6-well plate to make the bottom layer. The 6-well plate was incubated at 37°C to solidify. Cells were harvested and suspended at a concentration of 5 × 10^4^ cells/mL. The 0.7% soft-agar was mixed with 2 × DMEM containing 10% FBS at a ratio of 50:50. In total, 100 μL cell suspension was added into 1.5 mL agar/medium mixture and was immediately poured into the bottom layer plate. The plate was incubated at 37°C in a humidified atmosphere with 5% CO_2_ for 3 weeks.

### Plasmid constructs, small interfering RNA (siRNA), and miRNA mimic and transfection.

For functional analyses, the ORF of human SLC25A37, BID, UCK2, MORC2, IMP4, PA2G4, OGFOD2, RILPL1, DAPK1, DNMT3A, DNMT3B, CPSF1, CSTF2, CPSF7, NUDT21, FIP1, and PCF11 were cloned into eukaryotic expression vector pcDNA3.1. All siRNA and miRNA mimics were purchased from GenePharma. Transfection of plasmid, siRNA, or miRNA mimic was performed with Lipofectamine 2000 (Invitrogen) according to the manufacturer’s instruction. The sequences of primers, siRNAs, and miRNA mimics are listed in Supplemental Table 3.

### Generation of stable cell lines.

As previously described (36), to generate the MORC2-KO cell line, lentiCRISPR v2 vector expressing MORC2-KO sgRNA (5′-ACACCTGAGTCTACTCAGAT-3′) was transfected into Caki-1 cells. The cells were selected with puromycin, and then the selected clone was sequenced for validation. To generate the MORC2 S-KO cell lines with deletion of proximal PAS of MORC2, the MORC2 pPAS KO sgRNA-1 (5′-ATGGGTTGGTGGTCGCACCT-3′) and MORC2 pPAS KO sgRNA-2 (5′-GCAACCCAGTATGGCCAAAG-3′) guide RNAs were used. The deletion of the DNA region between the 2-target sgRNA was confirmed by MORC2 pPAS–KO forward primer (5′-GGAAAGGTGAGCCCCCCAATC-3′) and MORC2 pPAS–KO reverse primer (5′-AGATGAGGTGGTTAGACAGG-3′).

### qPCR and quantification of the ratio of long 3′UTR transcript/total transcripts.

Total RNA was extracted from cells with Trizol reagent (MilliporeSigma) and reverse transcribed into cDNA with SuperScript III Reverse Transcriptase Kit (Invtriogen). The qPCR assays were performed with SYBR-GREEN (Roche). The canonical 2^–ΔΔCt^ method was applied to determine the ratio of long 3′UTR transcript to total transcripts. As previously described (16), a pair of primers targeting the ORF was designed to represent the total transcripts, and another pair of primers targeting sequences just before the dPAS to represent the long transcripts. The ratio of long 3′UTR transcript/total transcripts was defined as the percentage of distal PAS usage index (PDUI). The degree of difference of long transcript/total transcripts ratio between tumor and normal tissue was used to identify 3′UTR lengthening (positive index) or 3′UTR shortening (negative index) in tumors. The primers are listed in Supplemental Table 2.

### Immunofluorescence staining.

Immunofluorescence staining was performed as previously described (30). Briefly, after transfection as indicated, cells were fixed in 4% PFA, permeabilized with Triton X-100, and blocked with goat serum. Next, cells were incubated with primary anti-MORC2 antibody (A300-149, Bethyl Laboratories) and Flag (F1804, MilliporeSigma) overnight at 4°C, and secondary antibody (Thermo Fisher Scientific) for 1 hour at 37°C. Finally, cells were stained by DAPI (Servicebio) and imaged with confocal microscope (Leica).

### Immunoblot analysis, IHC staining, and KIRC tissues.

Immunoblotting was performed according to standard protocol. Briefly, cells were harvested and lysed with NP-40 buffer, including NP-40, NaCl, Tris (pH 8.0), and inhibitors of protease and phosphatase. Bicinchoninic acid (BCA) assay was used to quantify protein concentration. Protein was separated by SDS-PAGE and then transferred to PVDF membrane (MilliporeSigma). After blocking with 5% BSA, the PVDF membrane was incubated with primary antibody overnight at 4°C and secondary antibody for 1 hour at room temperature. The images of immunoblot were obtained with Gel Documentation System (Syngene). The antibodies against MORC2 (Bethyl Laboratories), Flag (MilliporeSigma), DAPK1 (A5741, Abclonal), GFP (AE012, Abclonal), DNMT3A (32578, Cell Signaling Technology [CST]), DNMT3B (57868, CST), NUDT21 (A4482, Abclonal), 5mc (ab214727, Abcam), and β-actin (AC026, Abclonal) were used. IHC staining was performed as described in our previous work (37). The expression of indicated proteins was evaluated using the following standard (23). Staining intensity was scored as 0, negative; 1, weak; 2, moderate; and 3, strong. The percentage of positive staining cells was scored as 0, < 1%; 1, 1%–25%; 2, 26%–50%; 3, 51%–75%; and 4, >75%. The score of each tumor sample was obtained by multiplying staining intensity by percentage of positive staining cells. Tumor samples with a final score of 0–4 were defined as low expression, and those with a score of 6-12 were defined as high expression. The KIRC and adjacent normal tissues were collected from The Third-Affiliated Hospital of Sun Yat-sen University.

### 3′RACE assay.

3′Full Race Core Set (Takara) was used to generate MORC2 cDNA. MORC2 GSP-1 primers and 3′RACE outer primers were used to perform the first-round PCR. MORC2 GSP-2 primers and 3′RACE inner primers were used to perform the nested PCR. The PCR products were cloned with TA/Blunt-Zero kit (Vazyme) and further sequenced. The primers are listed in Supplemental Table 1.

### Xenograft tumor formation experiment.

The 5-week-old male BALB/c nude mice were purchased from Guangdong Sijiajingda Biotechnology and randomly divided into different groups (5 mice per group). A total of 1 × 10^6^ or 2 × 10^6^ Caki-1 cells with indicated treatment were injected s.c. into BALB/c nude mice. To test the in vivo effect of ASO-5, as previously described (38), a total of 1 × 10^6^ Caki-1 cells transfected with ASO-5 or control ASO were injected s.c. into BALB/c nude mice. Four or 5 weeks after injection, the mice were sacrificed and the tumors were collected.

### ChIP and MeDIP.

We performed ChIP with commercial ChIP assay kit (Beyotime) according to the manufacturer’s instruction. Briefly, Caki-1 cells were treated with 1% formaldehyde at 37°C for 10 minutes, and glycine solution was added at room temperature for 5 minutes. Next, the cells were washed with PBS, harvested, and lysed with SDS lysis buffer. The cell lysate was sonicated to break DNA into 200–1000 bp fragments. The solution was diluted with dilution buffer, and a small portion was set aside as input. After preclearance with Protein A + G Agarose containing salmon sperm DNA, the solution was immunoprecipitated with antibodies listed in Supplemental Table 5 at 4°C overnight. The solution was added with Protein A + G Agarose containing salmon sperm DNA at 4°C for 1 hour. The beads were washed once with low salt immune complex wash buffer, high salt immune complex wash buffer, LiCl immune complex wash buffer, and twice with TE buffer. The complex was washed twice with elution buffer at room temperature. NaCl was added for cross-linking reversal at 65°C for 4 hours. Finally, after DNA purification, the immunoprecipitated DNA was analyzed by qPCR or PCR with primers listed in Supplemental Table 2.

For MeDIP, DNA was isolated from Caki-1 cells, dissolved in TE buffer, and sonicated. DNA was denatured and incubated with 5mc antibody (Abcam) at 4°C overnight. The immune complex was pulled down with magnetic protein A beads (CST) at 4°C for 1 hour. The captured beads were washed for 3 times with 0.1% SDS, 1% Triton X-100, 20 mM Tris-HCl (pH 8.0), 2 mM EDTA, 150 mM NaCl, and then resuspended in TE buffer. The DNA was recovered with 50 mM Tris-HCl (pH 8.0), 10 mM EDTA, 0.5% SDS, and 35 μg proteinase K at 65°C for 3 hours and analyzed by qPCR with primers listed in Supplemental Table 2.

### Bioinformatic analysis.

For analysis of gene expression in KIRC and normal kidney, GEPIA database (gepia.cancer-pku.cn), and UALCAN database (https://ualcan.path.uab.edu) were used. For analysis of prognosis association in KIRC, KM Plotter database (https://kmplot.com/analysis/), GEPIA database, and UALCAN database were used. For analysis of polyA signals, NCBI database (https://www.ncbi.nlm.nih.gov) was used. For prediction of CpG island, UROGENE database (http://www.urogene.org/) was used. For analysis of DNA methylation in KIRC and normal kidney, UALCAN database was used. For analysis of AS in KIRC, TCGA Splicing Variants Database (TSVdb) (http://www.tsvdb.com) and TCGA SpliceSeq Database (https://bioinformatics.mdanderson.org/TCGASpliceSeq) were used.

### RNA-Seq.

The RNA-Seq was performed by LC-Bio. Briefly, Total RNA was extracted using Trizol reagent, and mRNA was purified from total RNA (5 μg) using Dynabeads Oligo (dT) (Thermo Fisher Scientific) with 2 rounds of purification. Following purification, the mRNA was fragmented into short fragments; then, the cleaved RNA fragments were reverse transcribed to create the cDNA by SuperScript II Reverse Transcriptase (Invitrogen), which was next used to synthesise U-labeled second-stranded DNA with *E. coli* DNA polymerase I (NEB), RNase H (NEB), and dUTP Solution (Thermo Fisher). An A-base was then added to the blunt ends of each strand, preparing them for ligation to the indexed adapters. Each adapter contained a T-base overhang for ligating the adapter to the A-tailed fragmented DNA. Dual-index adapters were ligated to the fragments, and size selection was performed with AMPure XP beads (Beckman). After the heat-labile UDG enzyme (NEB) treatment of the U-labeled second-stranded DNA, the ligated products were amplified with PCR by the following conditions: initial denaturation at 95°C for 3 minutes; 8 cycles of denaturation at 98°C for 15 seconds, annealing at 60°C for 15 seconds, and extension at 72°C for 30 seconds; and then final extension at 72°C for 5 minutes. The average insert size for the final cDNA libraries were 300 ± 50 bp. At last, we performed the 2 × 150 bp paired-end sequencing (PE150) on an Illumina Novaseq 6000 following the vendor’s recommended protocol. The differentially expressed mRNAs were selected with fold change > 2 or fold change < 0.5 and *P* < 0.05 by R package edgeR (https://bioconductor.org/packages/release/bioc/html/edgeR.html) or DESeq2 (http://www.bioconductor.org/packages/release/bioc/html/DESeq2.html).

### Luciferase reporter assay.

The short 3′UTR and long 3′UTR of MORC2 were cloned downstream of the Renila luciferase in psiCHECK-2. After transfection for 48 hours, luciferase activity was tested using the Dual-Luciferase Reporter Assay Kit (Promega).

### RNA EMSA.

RNA EMSA was performed as previously reported (11). For EMSA, GST-NUDT21 fusion protein was prepared following standard protocol (39). The biotin-labeled RNA was incubated with purified GST-NUDT21 form RNA-protein complex, and then the complex was separated by a 4% nondenaturing polyacrylamide gel in 0.5 × TBE. After electrophoresis, the gel and nylon membrane were assembled into a sandwich and transferred with cold 0.5 × TBE. Next, the membrane was placed in a commercial UV-light crosslinking instrument and crosslinked at 120 mJ/cm^2^ with 254 nm bulbs. Then, the crosslinked membrane was detected by Chemiluminescence (Vazyme). The sequences of biotin-labeled RNA oligos are listed in Supplemental Table 4.

### Co-IP.

Co-IP assay was performed according to standard protocol. Briefly, Cells were lysed with standard IP lysis buffer. Protein A/G beads (Santa Cruz Biotechnology Inc.) were incubated with anti-MORC2 or anti-DNMT3A antibodies for 4 hours at 4°C. Then, the antibody-conjugated beads were incubated with cell lysate (1 mg) at 4°C for 2 hours. The immunoprecipitation complexes were washed and subject to immunoblot.

### ASO and biotinylated ASO-RNA pull-down assay.

The ASOs used in this study were produced by Gukebai Bio. The ASOs are uniformly 2’-O-methyl phosphorothioate (2’-OMePS) modified. The transfection of ASO was performed with Lipofectin (Invitrogen). The sequences of ASOs are listed in Supplemental Figure 5. The biotinylated ASO-RNA pull-down assay was performed as previously described (40, 41); briefly, the negative control ASO and NUDT21 ASO-5 were labeled with biotin at the 3′ terminus. Twenty-four hours after transfection of biotinylated ASOs, whole-cell lysates were harvested. We prepared the streptavidin-coated magnetic bead (MCE) according to the manufacturer’s instruction. Biotinylated ASO-bound RNA was pulled down with streptavidin-coated beads. The RNA isolated from streptavidin-coated beads was then analyzed by qPCR with primers specific to the uORF of NUDT21 mRNA. The sequences of primers are listed in Supplemental Table 2.

### Statistics.

In each experiment, data were presented as the mean ± SD. Two-tailed Student’s *t* test was used to analyze the statistical significance between groups. One-way ANOVA with Tukey multiple-comparison test was used for multiple groups. Two-way ANOVA with Tukey multiple-comparison test was used to analyze data with 2 variables. The Pearson correlation test was used to analyze the correlation. Data analysis was performed with GraphPad Prism 8.0. *P* < 0.05 was considered significant.

### Study approval.

All animal experiments were approved by the IACUC of The Third Affiliated Hospital of Sun Yat-Sen University. This study was approved by the Ethics Committee of The Third Affiliated Hospital of Sun Yat-sen University.

### Data availability.

RNA-Seq data sets can be found at NCBI Gene Expression Omnibus (accession GSE221129). Values for all data points in graphs are reported in the Supporting Data Values file.

## Author contributions

NN, YT, and TZ designed the experiments. NN and KL revised the manuscript. KL collected clinical specimens and tissues. The order of co–first authors YT, TZ, ZS, and MC was determined by their degrees of contribution. YT performed most of the experiments. TZ performed some experiments, analyzed the data, and wrote the manuscript. ZS and MC performed the in vivo experiments. SC designed the ASO. RZ and RW contributed to the in vivo experiments. All authors approved the final version of the manuscript.

## Supplementary Material

Supplemental data

Supporting data values

## Version 1 09/22/2023

Electronic publication
