# Supplementary material for: Alternative polyadenylation reprogramming of MORC2 induced by NUDT21 loss promotes KIRC carcinogenesis
Source: JCI Insight. 2023 Sep 22;8(18):e162893. doi: 10.1172/jci.insight.162893 (PMC10561724; doi:10.1172/jci.insight.162893)
Supplement: Supplemental data [file jciinsight-8-162893-s031.pdf]

Supplemental Material

Alternative polyadenylation reprogramming of MORC2 induced by NUDT21 loss promotes KIRC carcinogenesis

Yuqin Tan, Tong Zheng, Zijun Su, Min Chen, Suxiang Chen, Rui Zhang, Ruojiao Wang, Ke Li, and Ning Na

This file contains Supplemental Figures, Supplemental Tables and Supplemental Data.

Supplemental Figures

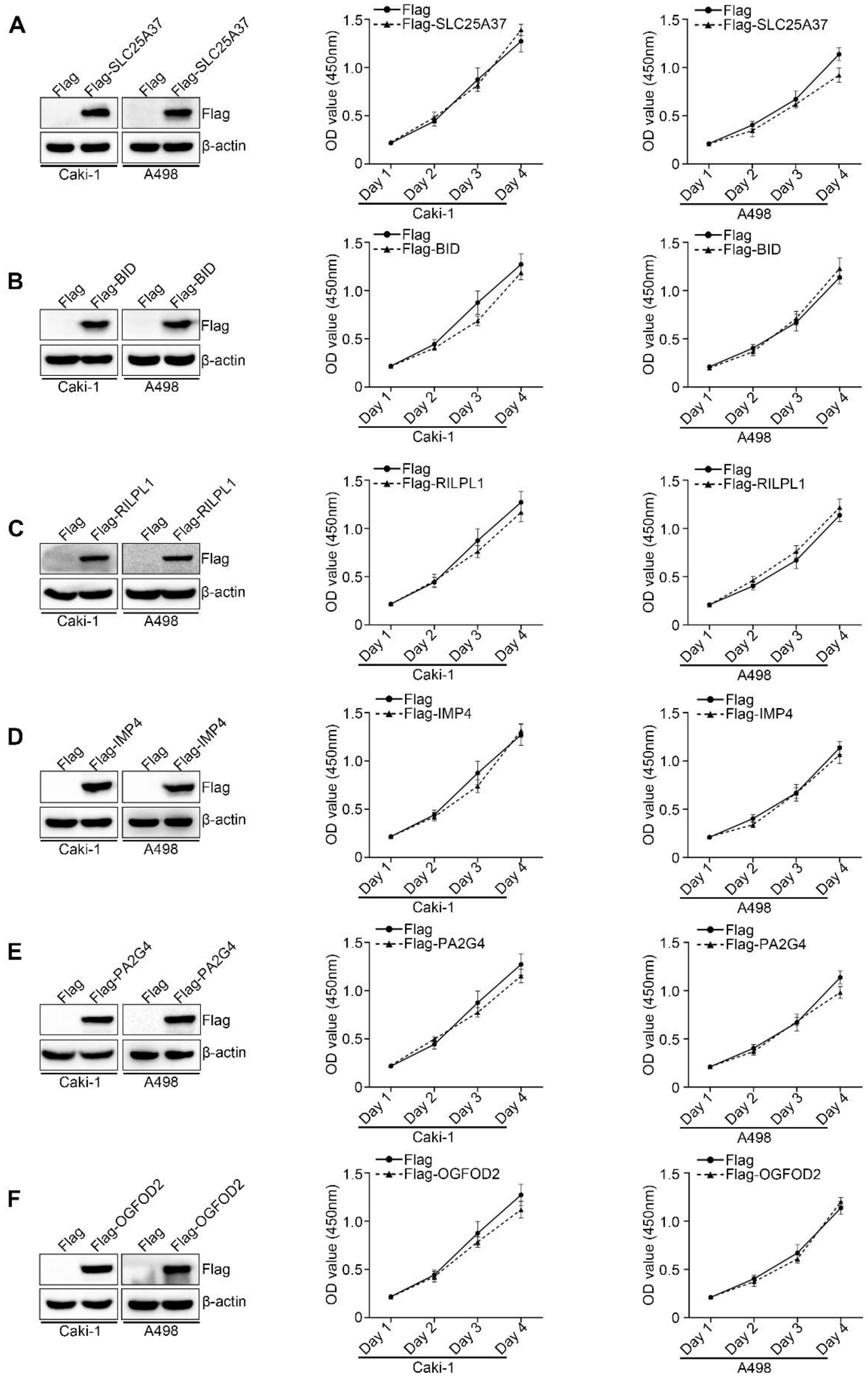

Supplemental Figure 1. Among the genes with shortened 3'UTR in KIRC, *UCK2* and *MORC2* gain oncogenic potential.

(**A**) CCK8 assay was performed to evaluate the proliferation rate of Caki-1 cells and

A498 cells transfected with empty Flag vector, Flag-SLC25A37 plasmid respectively. (**B**) CCK8 assay was performed to evaluate the proliferation rate of Caki-1 cells and A498 cells transfected with empty Flag vector, Flag-BID plasmid respectively. (**C**) CCK8 assay was performed to evaluate the proliferation rate of Caki-1 cells and A498 cells transfected with empty Flag vector, Flag-RILPL1 plasmid respectively. (**D**) CCK8 assay was performed to evaluate the proliferation rate of Caki-1 cells and A498 cells transfected with empty Flag vector, Flag-IMP4 plasmid respectively. (**E**) CCK8 assay was performed to evaluate the proliferation rate of Caki-1 cells and A498 cells transfected with empty Flag vector, Flag-PA2G4 plasmid respectively. (**F**) CCK8 assay was performed to evaluate the proliferation rate of Caki-1 cells and A498 cells transfected with empty Flag vector, Flag-OGFOD2 plasmid respectively.

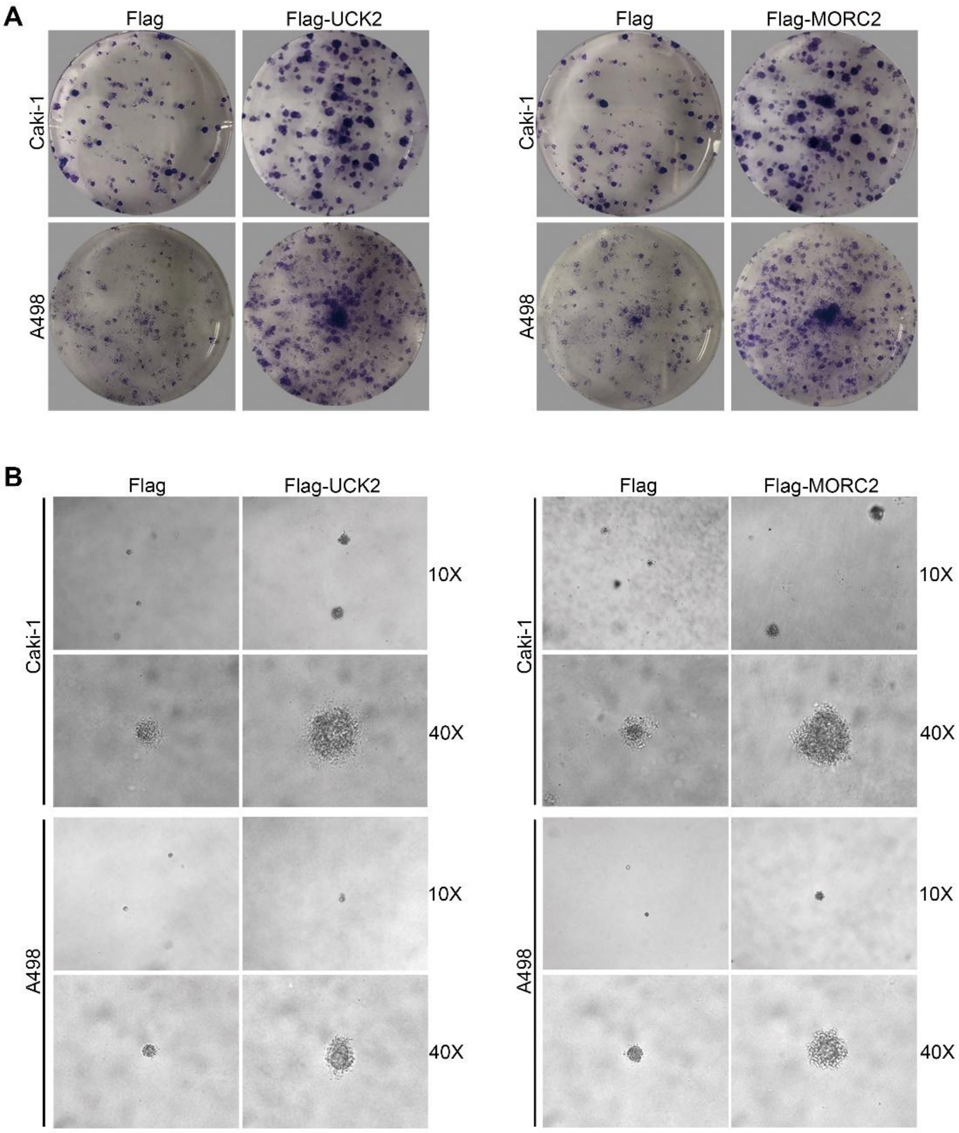

Supplemental Figure 2. Ectopic expression of UCK2 or MORC2 favors the clonogenicity of KIRC cells.

(**A**) The representative images of the clonogenicity of Caki-1 cell and A498 cells transfected with empty Flag vector, Flag-UCK2 or Flag-MORC2 plasmid respectively in colony formation assays. (**B**) The representative images of the clonogenicity of Caki-1 cell and A498 cells transfected with empty Flag vector, Flag-UCK2 or Flag-MORC2 plasmid respectively in soft agar assays.

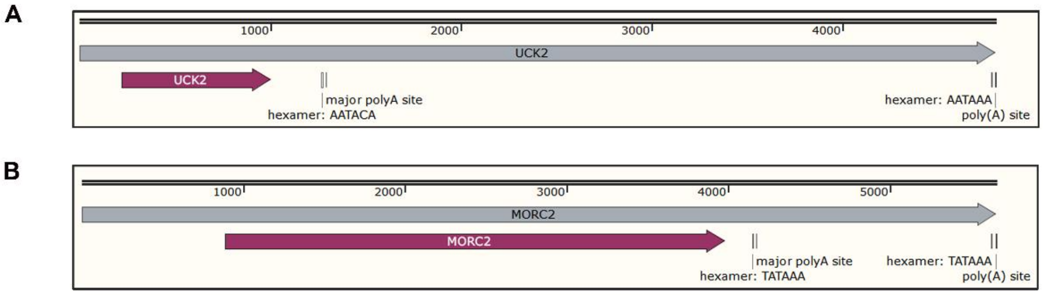

C

mRNA	sequence	position	Proximal or distal	Length
UCK2	AATACA	1269-1274	proximal	6 nt
UCK2	AATAAA	4781-4786	distal	6 nt
MORC2	TATAAA	4152-4157	proximal	6 nt
MORC2	TATAAA	5629-5634	distal	6 nt

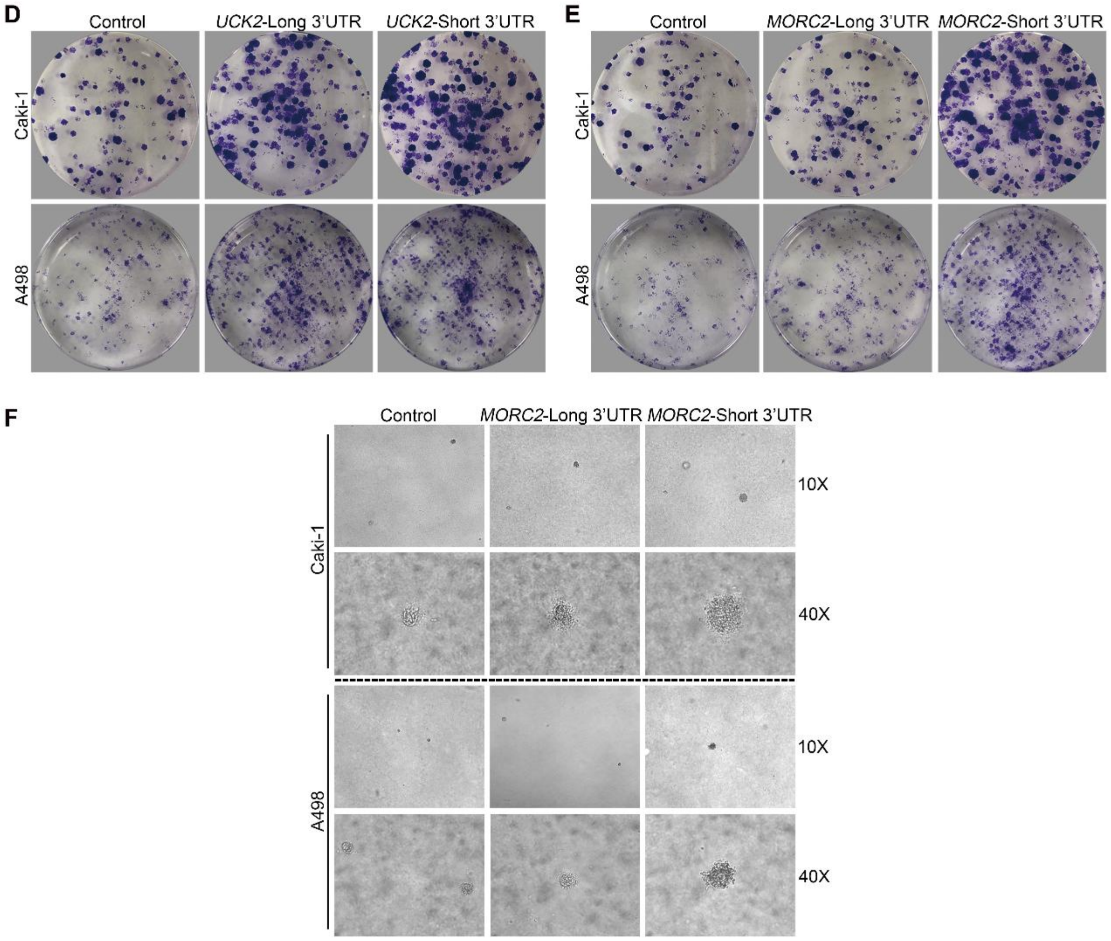

Supplemental Figure 3. 3'UTR shortening enhances oncogenic potential of *MORC2* in KIRC via upregulating MORC2.

(**A**) Schematic diagram indicating the proximal polyA site (pPAS) of *UCK2* from NCBI Database. (**B**) Schematic diagram indicating the proximal polyA site (pPAS) of *MORC2* from NCBI Database. (**C**) The diagram indicating the detailed information of pPAS of *UCK2* and *MORC2* from NCBI Database. (**D**) The representative images of

the clonogenicity of Caki-1 cell and A498 cells transfected with short- or long-3'UTR *UCK2* plasmid respectively in colony formation assays. (**E**) The representative images of the clonogenicity of Caki-1 cell and A498 cells transfected with short- or long-3'UTR *MORC2* plasmid respectively in colony formation assays. (**F**) The representative images of the clonogenicity of Caki-1 cell and A498 cells transfected with short- or long-3'UTR *MORC2* plasmid respectively in soft agar assays.

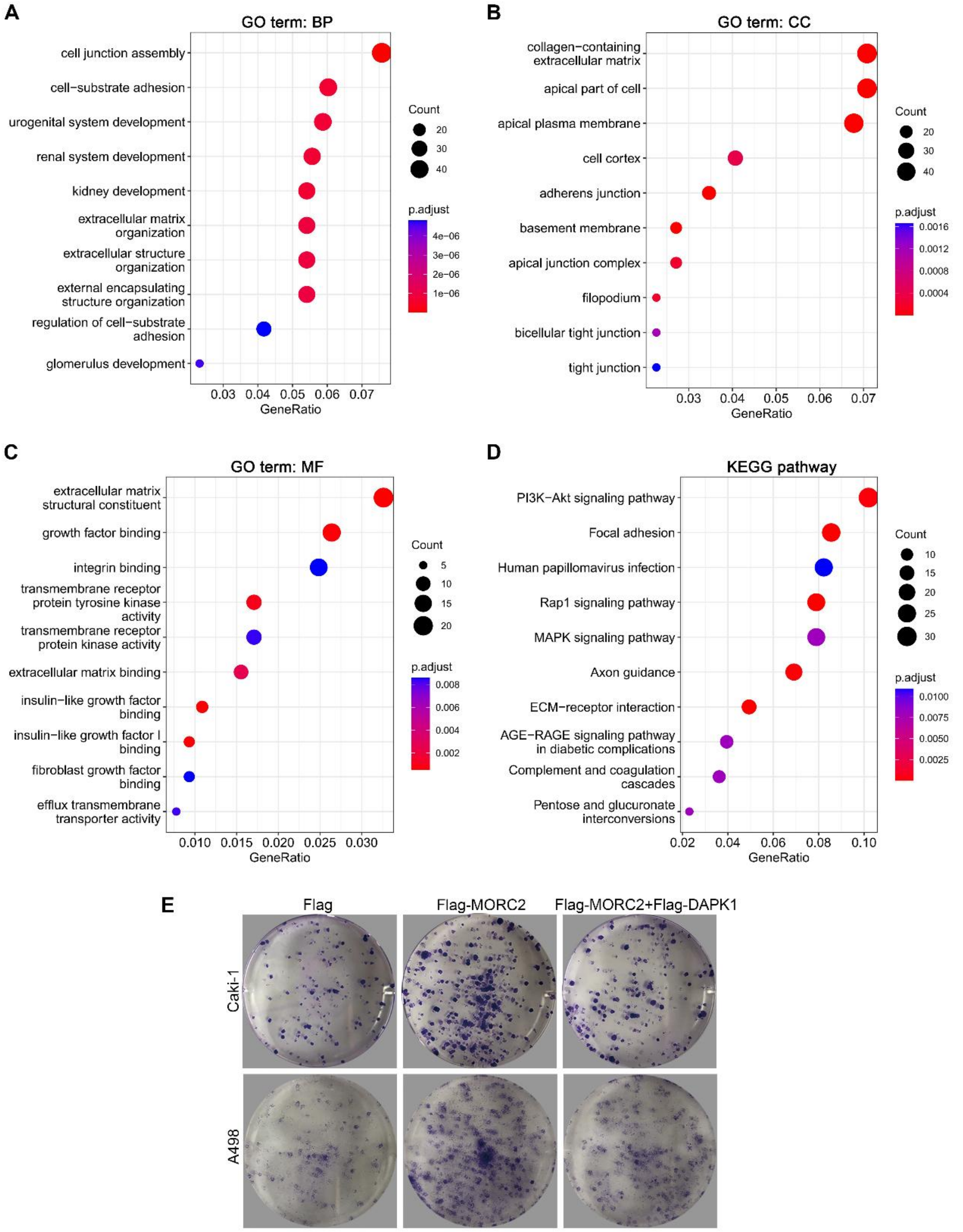

Supplemental Figure 4. *MORC2* acts as oncogene in KIRC mainly depending on downregulating tumor suppressor DAPK1 via DNA methylation.
(**A - C**) The GO analysis of the genes downregulated by MORC2. (**D**) The KEGG analysis of the genes downregulated by MORC2. (**E**) The representative images of the clonogenicity of Caki-1 cell and A498 cells transfected with empty Flag vector or Flag-MORC2 plasmid with or without DAPK1 recovery.

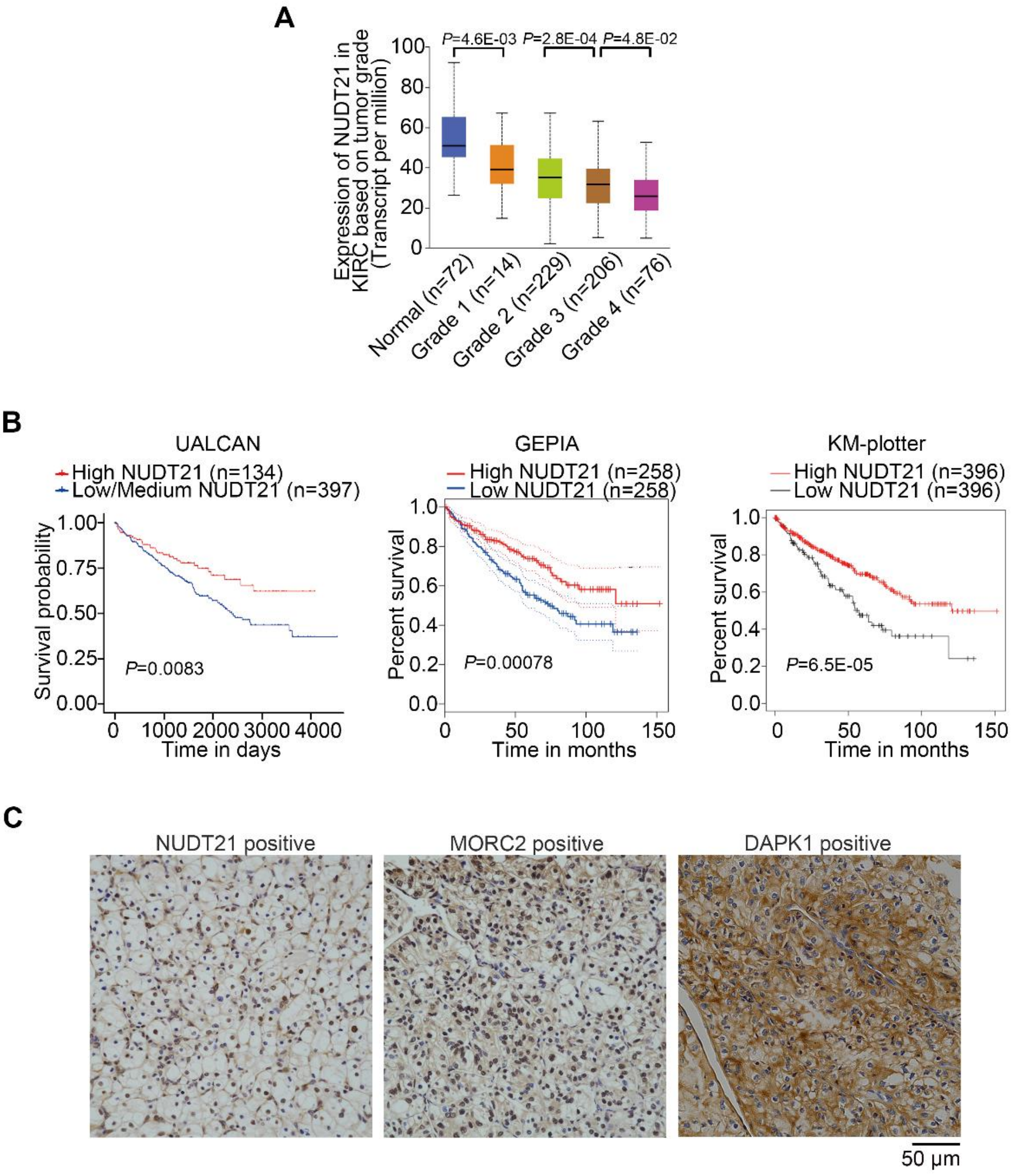

Supplemental Figure 5. Loss of APA regulator NUDT21 induces 3'UTR shortening and downregulation of MORC2 in KIRC.

(**A**) The association between *NUDT21* expression and tumor grade of KIRC was analyzed with UALCAN database. (**B**) Prognosis association of *NUDT21* in KIRC was analyzed with UALCAN (left), GEPIA (middle) and KM plotter (right) databases. (**C**) The representative IHC images of NUDT21-positve, MORC2-positive and DAPK1-positve specimens. Magnification: 40 ×.

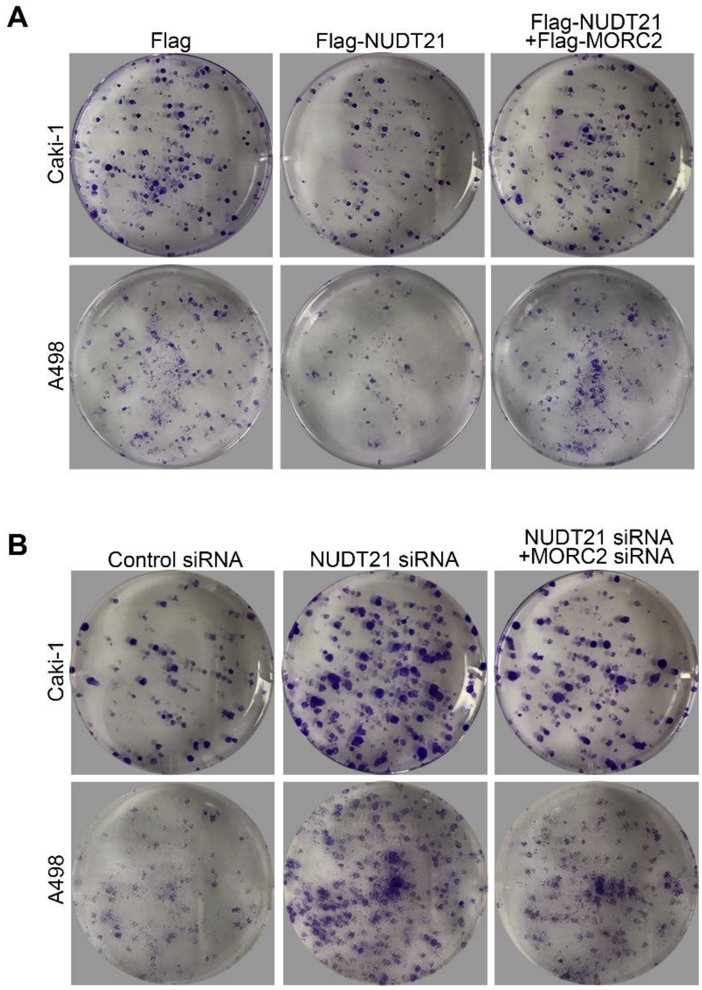

Supplemental Figure 6. NUDT21 functions as a tumor suppressor mainly depending on MORC2 downregulation.

(**A**) The representative images of the clonogenicity of Caki-1 cell and A498 cells transfected with empty Flag vector or Flag-NUDT21 with or without MORC2 recovery. (**B**) The representative images of the clonogenicity of Caki-1 cell and A498 cells transfected with empty Flag vector or Flag-NUDT21 with or without MORC2 recovery.

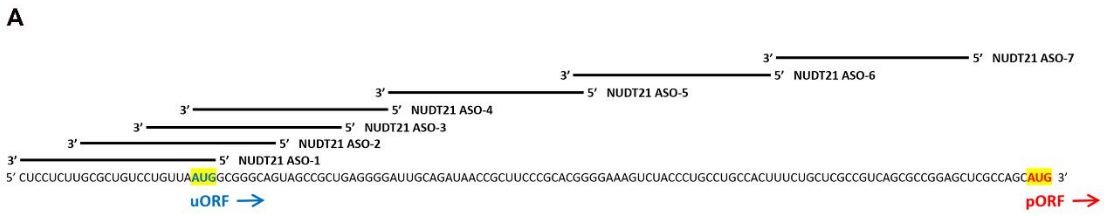

B

NUDT21 ASO-1 (+1+25): CAUUAACAGGACAGCGCAAGAGGAG Targeting region: CUCCUCUUGCGCUGUCCUGUUAAUG

NUDT21 ASO-2 (+9+33): CUGCCCGCCAUUAACAGGACAGCGC Targeting region: GCGCUGUCCUGUUAAUGGCGGGCAG

NUDT21 ASO-3 (+17+41): AGCGGCUACUGCCCGCCAUUAACAG Targeting region: CUGUUAAUGGCGGGCAGUAGCCGCU

NUDT21 ASO-4 (+23+47): CCCCUCAGCGGCUACUGCCCGCCAU Targeting region: AUGGCGGGCAGUAGCCGCUGAGGGG

NUDT21 ASO-5 (+48+72): CGUGCGGGAAGCGGUUAUCUGCAAU Targeting region: AUUGCAGAUAACCGCUUCCCGCACG

NUDT21 ASO-6 (+73+97): AGUGGCAGGCAGGGUAGACUUUCCC Targeting region: GGGAAAGUCUACCCUGCCTGCCACU

NUDT21 ASO-7 (+98+122): GCUCCGGCGCUGACGGCGAGCAGAA Targeting region: UUCUGCUCGCCGUCAGCGCCGGAGC

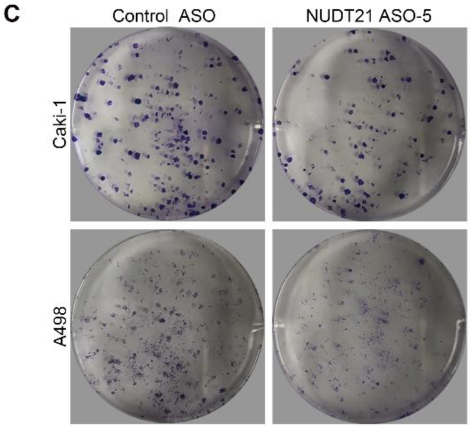

Supplemental Figure 7. Antisense oligonucleotides (ASOs) enhancing NUDT21 expression inhibits proliferation and clonogenicity of KIRC cells.

(**A**) The diagram indicating the region of *NUDT21* mRNA which ASO-1, ASO-1, ASO-3, ASO-4, ASO-5, ASO-6 or ASO-7 targets. (**B**) The diagram indicating the detailed information of ASO-1, ASO-1, ASO-3, ASO-4, ASO-5, ASO-6 and ASO-7. (**C**) The representative images of the clonogenicity of Caki-1 cell and A498 cells transfected with control ASO or ASO-5.

A

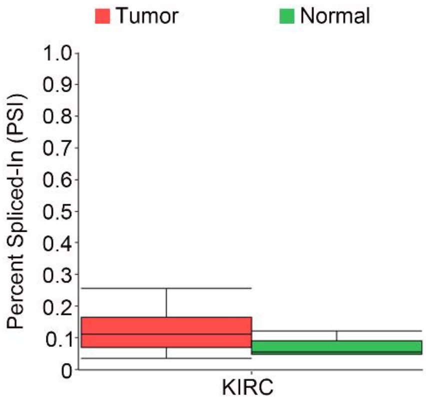

Supplemental Figure 8. The Percent Spliced-In of *MORC2* in KIRC and normal kidney.

(**A**)The Percent Spliced-In of *MORC2* in KIRC and normal kidney was analyzed with TCGA SpliceSeq Database database.

A

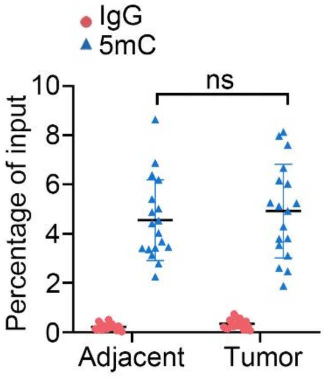

Supplemental Figure 9. The level of promoter methylation of *MORC2* is similar in KIRC tissues and matched kidney tissues.

(**A**) MeDIP assay was performed to evaluate the level of promoter methylation of *MORC2* in KIRC tissues and matched kidney tissues.

Supplemental Tables

Supplemental Table 1. Primers used for 3'RACE (5'-3')

Primers	Sequence
MORC2 GSP-1	GGGGACTGAAGGCAGGAGAGAGAGCAGC
MORC2 GSP-2	GGTCGCACCTTGGTTTGACTTACACGGG

Supplemental Table 2. Primers used for plasmid constructions, qRT-PCR, ChIP/MeDIP and MORC2 3'UTR isoform specific PCR (5'-3')

Primers	Sequence
SLC25A37-F	ATGGAGCTGCGCAGCGGGAGC
SLC25A37-R	GTATGGAGCTCGATTTTCCAG
BID-F	ATGGACTGTGAGGTCAACAAC
BID-R	GTCCATCCCATTTCTGGCTAA
UCK2-F	ATGGCCGGGGACAGCGAGCAG
UCK2-R	ATGCGGCCTGCTGCTGGACTC
MORC2-F	ATGGCTTTCACAAATTACAGC
MORC2-R	GTCCCCCTTGGTGATGAGGTC
IMP4-F	ATGCTGCGCCGCGAGGCCCGC
IMP4-R	CTCGGTGCTCAGGAAGACTCT
PA2G4-F	ATGTCGGGCGAGGACGAGCAA
PA2G4-R	GTCCCCAGCTTCATTTTCTTC
OGFOD2-F	ATGGCGACGGTGGGGGCTCCG
OGFOD2-R	GGTGAGCGCACATACATCCAC
RILPL1-F	ATGGAGGAGGAGCGGGGGTCG
RILPL1-R	CAGATGCTGCAGGGCTTCCTG
DAPK1-F	ATGACCGTGTTCAGGCAGGAA
DAPK1-R	CCGGGATACAACAGAGCTAAT
DNMT3A-F	ATGCCCGCCATGCCCTCCAGC

DNMT3A-R	CACACACGCAAAATACTCCTT
DNMT3B-F	ATGAAGGGAGACACCAGGCAT
DNMT3B-R	TTCACATGCAAAGTAGTCCTT
CPSF1-F	ATGTACGCCGTGTACAAACAG
CPSF1-R	GAAGTGGGCGGTGACGCGGTC
CSTF2-F	ATGGCGGGTTTGACTGTGAGA
CSTF2-R	AGGTGCTCCAGTGGATTTCTG
CPSF7-F	ATGGGACGGCCGGAGTCTGCG
CPSF7-R	GTGGTGCCGGTCCCGTTCTCT
NUDT21-F	ATGTCTGTGGTACCGCCCAAT
NUDT21-R	GTTGTAAATAAAATTGAACCT
PCF11-F	ATGTCAGAGCAGACGCCGGCC
PCF11-R	AACTGACTCGACTGTGTCATT
FIP1-F	ATGTCGGCCGGCGAGGTCGAG
FIP1-R	TTCTGCAGGTGTAGCTTCGGT
MORC2 pPAS-KO-F	GGAAAGGTGAGCCCCCCAATC
MORC2 pPAS-KO-R	AGATGAGGTGGTTAGACAGG
NUDT21 upstream specific-F	TGTTAATGGCGGGCAGTAGC
NUDT21 upstream specific-R	GACGGCGAGCAGAAAGTGGC
RT-*GAPDH*-F	ACAACTTTGGTATCGTGGAAGG
RT-*GAPDH*-R	GCCATCACGCCACAGTTTC
RT-*HPRT*-F	CCTGGCGTCGTGATTAGTGAT
RT-*HPRT*-R	AGACGTTCAGTCCTGTCCATAA
RT-*DAPK1*-F	ACGTGGATGATTACTACGACACC
RT-*DAPK1*-R	TGCTTTTCTCACGGCATTTCT
RT-*NUDT21*-F	GGTCACTCAGTTCGGCAACAA
RT-*NUDT21*-R	CTCATGCGCTGAAATCTGGC
RT-*MORC2*-Total-F	ATGAAGTAGGGCTCCAAAAC
RT-*MORC2*-Total-R	GGTCCTCAATGTAGGCGTCC

RT-*MORC2*-distal-F	CTGGATGTGCCTGTCTGGTGG
RT-*MORC2*-distal-R	TGAAGAGAGAGAGCAGGATGG
DAPK1-ChIP-PCR-F	GCCCAGCTTTAACAAAGGGTG
DAPK1-ChIP-PCR-R	CATGCATCAGTCTCCAGTCCT
DAPK1-ChIP-qPCR-F	GCCCAGCTTTAACAAAGGGTG
DAPK1-ChIP-qPCR-R	GCTAAGACCCCGCTCGCTG
NUDT21-ChIP-PCR-F	TACTGCCCGCCATTAACAGG
NUDT21-ChIP-PCR-R	TCCAGCTATTGCAGCCATCC
NUDT21-ChIP-qPCR-F	GAAGTGAACCCGGAGCCTCT
NUDT21-ChIP-qPCR-R	TCCAGCTATTGCAGCCATCC
oligo(dT) primer	GCTGTCAACGATACGCTACGTAACGGCATGACAGTG$(T)_{18}$

Supplemental Table 3. Sequences of siRNA and CRISPR sgRNA (5’-3’)

siRNA or sgRNA	Sequence
Negative control siRNA	UUCUCCGAACGUGUCACGUTT
MORC2 siRNA	GGACAUGAAGACGCAGGAATT
NUDT21 siRNA	GCUAUAUACAGUGUAGAAUTT
DNMT3A siRNA-1	GCCUGGAGCCACCAGAAGATT
DNMT3A siRNA-2	GAAAGCCAAGGUCAUUGCATT
DNMT3B siRNA-1	GCAACGAUCUCUCAAAUGUTT
DNMT3B siRNA-2	GCUACACACAGGACUUGACTT
MORC2 KO sgRNA	ACACCTGAGTCTACTCAGAT
MORC2 pPAS KO sgRNA-1	ATGGGTTGGTGGTCGCACCT
MORC2 pPAS KO sgRNA-2	GCAACCCAGTATGGCCAAAG
Negative control ASO	CCUCUUACCUCAGUUACAAUUUAUA

Supplemental Table 4. Biotin labeled RNA oligos for RNA-EMSA (5’-3’-Biotin)

Oligos	Sequence
MORC2 WT	CACACACAUGUAAUCGAAAC
MORC2 Mutant:	CACACACACUCGAUCGAAAC

Supplemental Table 5. Antibodies used in this study

Antibody	Vender	Cat. No.
MORC2	Bethyl Laboratories	A300-149
Flag	Sigma-Aldrich	F1804
5mC	Abcam	ab214727
β-actin	Abclonal	AC026
GFP	Abclonal	AE012
DNMT3A	CST	32578
DNMT3B	CST	57868
DAPK1	Abclonal	A5741
NUDT21	Abclonal	A4482

Supplemental Data

Supplemental Data 1. Genes with shortened 3'UTR in KIRC

Event_id	KIRC
NM_024731|KLHL36|chr16|+	-0.460625
NM_001190738|NFIB|chr9|-	-0.455
NM_001135652|EIF2AK2|chr2|-	-0.45037037
NM_001142386|PDK3|chrX|+	-0.425714286
NM_152793|C7orf41|chr7|+	-0.422916667
NM_001164182|ENTPD1|chr10|+	-0.420857143
NM_003599|SUPT3H|chr6|-	-0.405909091
NM_018941|CLN8|chr8|+	-0.399583333
PPP2R5C|chr14|+	-0.373341211
NM_005349|RBPJ|chr4|+	-0.37125
NM_173829|SREK1IP1|chr5|-	-0.361875
NM_004928|C21orf2|chr21|-	-0.361724138
NM_001110792|MECP2|chrX|-	-0.358
NM_001134232|TMEM106B|chr7|+	-0.3568
NM_001098517|CADM1|chr11|-	-0.350625
NM_001173480|ARHGEF9|chrX|-	-0.348235294
NM_001040445|ASB1|chr2|+	-0.347407407
NM_015459|ATL3|chr11|-	-0.345
NR_002791|EMX2OS|chr10|-	-0.338235294
NR_023348|GAS8|chr16|+	-0.336153846
NM_015035|ZHX3|chr20|-	-0.334782609
NM_001424|EMP2|chr16|-	-0.334210526
NM_016418|NF2|chr22|+	-0.332666667
NM_175624|RAB3IP|chr12|+	-0.32952381
NM_001142389|PNPLA4|chrX|-	-0.328333333
NM_005793|NME6|chr3|-	-0.3275
NM_001004313|TMEM220|chr17|-	-0.324375
NM_205861|DHDDS|chr1|+	-0.322666667
NM_001204064|CHURC1|chr14|+	-0.32125
NM_003300|TRAF3|chr14|+	-0.320625
NM_015704|PPPDE2|chr22|-	-0.319310345
NM_153029|N4BP1|chr16|-	-0.318888889
NM_001037333|CYFIP2|chr5|+	-0.318181818
NM_005124|NUP153|chr6|-	-0.318125
NM_005759|ABI2|chr2|+	-0.316923077
GLS|chr2|+	-0.316702851
NM_001173431|OBSL1|chr2|-	-0.315333333
NM_016612|SLC25A37|chr8|+	-0.314857143

NM_001196|BID|chr22|-	-0.313076923
NM_000428|LTBP2|chr14|-	-0.311923077
NM_003875|GMPS|chr3|+	-0.311818182
NM_001105069|ACSM2B|chr16|-	-0.310555556
NM_004863|SPTLC2|chr14|-	-0.309047619
NR_033361|TMCC1|chr3|-	-0.308409091
NM_032812|PLXDC2|chr10|+	-0.308333333
NM_139062|CSNK1D|chr17|-	-0.307948718
NM_024632|SAP30L|chr5|+	-0.305789474
NM_020696|KIAA1143|chr3|-	-0.305
NM_000259|MYO5A|chr15|-	-0.303461538
NM_178314|RILPL1|chr12|-	-0.303181818
PCGF5|chr10|+	-0.302195945
NM_016422|RNF141|chr11|-	-0.300588235
NR_015341|LRRC37BP1|chr17|+	-0.3
NM_001076684|UBTF|chr17|-	-0.298666667
NM_002141|HOXA4|chr7|-	-0.298333333
NM_001202470|RPS10-NUDT3|chr6|-	-0.298235294
NM_001184723|TM4SF18|chr3|-	-0.298214286
NM_152416|C8orf38|chr8|+	-0.298
NM_152734|C6orf89|chr6|+	-0.295454545
NM_013321|SNX8|chr7|-	-0.295416667
NM_001012973|PLAC9|chr10|+	-0.294782609
DEF8|chr16|+	-0.294714747
NM_014965|TRAK1|chr3|+	-0.294705882
NM_015255|UBR2|chr6|+	-0.294615385
NM_178012|TUBB2B|chr6|-	-0.294583333
NM_032448|FAM120B|chr6|+	-0.293181818
NM_015944|AMDHD2|chr16|+	-0.293043478
NM_022899|ACTR8|chr3|-	-0.292142857
NM_003818|CDS2|chr20|+	-0.291764706
NM_012474|UCK2|chr1|+	-0.291111111
NM_005008|NHP2L1|chr22|-	-0.290869565
NM_014934|DZIP1|chr13|-	-0.2908
NM_012192|FXC1|chr11|+	-0.290714286
NM_013449|BAZ2A|chr12|-	-0.29
NM_015305|ANGEL1|chr14|-	-0.289411765
NM_018697|LANCL2|chr7|+	-0.2875
NM_001145354|MKLN1|chr7|+	-0.287333333
NM_004804|CIAO1|chr2|+	-0.2865
NM_012345|NUFIP1|chr13|-	-0.286470588
NM_015607|CHTOP|chr1|+	-0.28625
NM_002154|HSPA4|chr5|+	-0.2859375

NM_002936|RNASEH1|chr2|-	-0.285714286
NM_001206652|SH3GLB1|chr1|+	-0.285333333
NM_003177|SYK|chr9|+	-0.284117647
FLT1|chr13|-	-0.2839709
NM_030813|CLPB|chr11|-	-0.282222222
NM_003681|PDXK|chr21|+	-0.281875
BCCIP|chr10|+	-0.280160305
NM_006741|PPP1R1A|chr12|-	-0.28
NM_014941|MORC2|chr22|-	-0.279722222
NM_033416|IMP4|chr2|+	-0.279130435
NM_015321|CRTC1|chr19|+	-0.278888889
NM_016329|SFMBT1|chr3|-	-0.27875
NM_183323|PAIP1|chr5|-	-0.278
NM_017670|OTUB1|chr11|+	-0.277333333
NM_152388|TMEM237|chr2|-	-0.276470588
NM_020320|RARS2|chr6|-	-0.275
FGFR1OP2|chr12|+	-0.273595038
NM_033637|BTRC|chr10|+	-0.273571429
NM_012141|INTS6|chr13|-	-0.272142857
NM_001173513|TXNRD3|chr3|-	-0.271428571
RPP30|chr10|+	-0.269744303
NM_006110|CD2BP2|chr16|-	-0.269285714
NM_020374|C12orf4|chr12|-	-0.269166667
NAAA|chr4|-	-0.268142971
NM_153329|ALDH16A1|chr19|+	-0.267333333
NM_206831|DPH3|chr3|-	-0.267142857
NM_016141|DYNC1LI1|chr3|-	-0.266875
NM_183245|INVS|chr9|+	-0.266875
NM_006191|PA2G4|chr12|+	-0.2635
NM_172250|MMAA|chr4|+	-0.262666667
NM_203458|NOTCH2NL|chr1|+	-0.262
NM_015247|CYLD|chr16|+	-0.261428571
NM_003062|SLIT3|chr5|-	-0.260588235
PVRL2|chr19|+	-0.257987638
NM_138991|BACE2|chr21|+	-0.257777778
NM_152346|SLC43A2|chr17|-	-0.257727273
NM_017872|THG1L|chr5|+	-0.256315789
CYHR1|chr8|-	-0.255828417
NM_016185|HN1|chr17|-	-0.253157895
NM_001160305|SETD6|chr16|+	-0.253
NM_017994|C7orf42|chr7|+	-0.2525
NM_017896|C20orf11|chr20|+	-0.251764706
NM_018003|UACA|chr15|-	-0.250909091

NM_020662|MRS2|chr6|+	-0.248947368
NM_198901|SRI|chr7|-	-0.2455
NM_022455|NSD1|chr5|+	-0.245
NM_016138|COQ7|chr16|+	-0.242352941
NM_130759|GIMAP1|chr7|+	-0.242
NM_024562|TMCO7|chr16|+	-0.24125
NM_002634|PHB|chr17|-	-0.24
NM_012472|LRRC6|chr8|-	-0.238571429
NM_024623|OGFOD2|chr12|+	-0.221333333

Supplemental Data 2. Downregulated genes upon MORC2 overexpression

Gene_id	Gene_name	Fold change	log2(fc)	*P* value
ENSG00000137801	THBS1	0.03	-4.95	0
ENSG00000165376	CLDN2	0.01	-6.08	0
ENSG00000151892	GFRA1	0.02	-5.84	0
ENSG00000272398	CD24	0.01	-7.15	0
ENSG00000146674	IGFBP3	0.09	-3.54	0.00
ENSG00000120594	PLXDC2	0.10	-3.35	0.00
ENSG00000038427	VCAN	0.11	-3.20	0.00
ENSG00000122870	BICC1	0.18	-2.45	0.00
ENSG00000137731	FXYD2	0.07	-3.77	0.00
ENSG00000108861	DUSP3	0.14	-2.82	0.00
ENSG00000225339	AL354740	0.04	-4.57	0.00
ENSG00000101384	JAG1	0.14	-2.80	0.00
ENSG00000158258	CLSTN2	0.01	-6.26	0.00
ENSG00000137962	ARHGAP29	0.19	-2.37	0.00
ENSG00000168077	SCARA3	0.11	-3.12	0.00
ENSG00000134516	DOCK2	0.24	-2.06	0.00
ENSG00000113361	CDH6	0.27	-1.88	0.00
ENSG00000120885	CLU	0.25	-2.01	0.00
ENSG00000105855	ITGB8	0.09	-3.45	0.00
ENSG00000118898	PPL	0.10	-3.27	0.00
ENSG00000163453	IGFBP7	0.32	-1.65	0.00
ENSG00000065534	MYLK	0.24	-2.07	0.00
ENSG00000166833	NAV2	0.18	-2.49	0.00
ENSG00000198074	AKR1B10	0.16	-2.65	0.00
ENSG00000019505	SYT13	0.28	-1.85	0.00
ENSG00000134215	VAV3	0.08	-3.64	0.00
ENSG00000115221	ITGB6	0.12	-3.03	0.00
ENSG00000101333	PLCB4	0.20	-2.34	0.00
ENSG00000259207	ITGB3	0.30	-1.76	0.00
ENSG00000162692	VCAM1	0.27	-1.91	0.00
ENSG00000182168	UNC5C	0.04	-4.50	0.00
ENSG00000196730	DAPK1	0.18	-2.46	0.00
ENSG00000057019	DCBLD2	0.32	-1.63	0.00
ENSG00000152952	PLOD2	0.31	-1.71	0.00
ENSG00000112769	LAMA4	0.33	-1.61	0.00
ENSG00000145147	SLIT2	0.07	-3.88	0.00
ENSG00000206190	ATP10A	0.10	-3.30	0.00
ENSG00000091656	ZFHX4	0.26	-1.94	0.00
ENSG00000275410	HNF1B	0.28	-1.86	0.00
ENSG00000183018	SPNS2	0.04	-4.55	0.00
ENSG00000163235	TGFA	0.35	-1.50	0.00
ENSG00000136883	KIF12	0.10	-3.31	0.00
ENSG00000083857	FAT1	0.38	-1.40	0.00
ENSG00000120738	EGR1	0.16	-2.61	0.00
ENSG00000123485	HJURP	0.33	-1.61	0.00
ENSG00000064042	LIMCH1	0.31	-1.67	0.00
ENSG00000150938	CRIM1	0.33	-1.62	0.00
ENSG00000154229	PRKCA	0.38	-1.38	0.00
ENSG00000019991	HGF	0.02	-5.47	0.00
ENSG00000189056	RELN	0.25	-1.99	0.00
ENSG00000112175	BMP5	0.15	-2.78	0.00
ENSG00000109321	AREG	0.37	-1.42	0.00
ENSG00000198796	ALPK2	0.19	-2.43	0.00
ENSG00000173482	PTPRM	0.37	-1.44	0.00
ENSG00000053747	LAMA3	0.36	-1.48	0.00
ENSG00000090530	P3H2	0.33	-1.59	0.00
ENSG00000113249	HAVCR1	0.31	-1.70	0.00

ENSG00000149948	HMGA2	0.38	-1.39	0.00
ENSG00000142910	TINAGL1	0.20	-2.29	0.00
ENSG00000196139	AKR1C3	0.43	-1.22	0.00
ENSG00000148498	PARD3	0.38	-1.38	0.00
ENSG00000086062	B4GALT1	0.39	-1.34	0.00
ENSG00000134871	COL4A2	0.43	-1.23	0.00
ENSG00000158089	GALNT14	0.31	-1.68	0.00
ENSG00000133131	MORC4	0.38	-1.40	0.00
ENSG00000112414	ADGRG6	0.09	-3.42	0.00
ENSG00000187498	COL4A1	0.42	-1.25	0.00
ENSG00000105329	TGFB1	0.33	-1.62	0.00
ENSG00000143126	CELSR2	0.24	-2.03	0.00
ENSG00000143674	MAP3K21	0.32	-1.64	0.00
ENSG00000134901	POGLUT2	0.32	-1.66	0.00
ENSG00000169439	SDC2	0.17	-2.59	0.00
ENSG00000242265	PEG10	0.38	-1.41	0.00
ENSG00000117115	PADI2	0.18	-2.51	0.00
ENSG00000163629	PTPN13	0.26	-1.92	0.00
ENSG00000075223	SEMA3C	0.38	-1.39	0.00
ENSG00000182809	CRIP2	0.17	-2.53	0.00
ENSG00000265972	TXNIP	0.40	-1.31	0.00
ENSG00000148468	FAM171A1	0.32	-1.65	0.00
ENSG00000054654	SYNE2	0.21	-2.28	0.00
ENSG00000169604	ANTXR1	0.36	-1.49	0.00
ENSG00000078725	BRINP1	0.09	-3.54	0.00
ENSG00000152377	SPOCK1	0.42	-1.24	0.00
ENSG00000188483	IER5L	0.27	-1.91	0.00
ENSG00000185551	NR2F2	0.44	-1.20	0.00
ENSG00000064393	HIPK2	0.44	-1.17	0.00
ENSG00000168502	MTCL1	0.37	-1.44	0.00
ENSG00000146555	SDK1	0.20	-2.31	0.00
ENSG00000130702	LAMA5	0.32	-1.62	0.00
ENSG00000124602	UNC5CL	0.07	-3.84	0.00
ENSG00000152818	UTRN	0.27	-1.87	0.00
ENSG00000188158	NHS	0.16	-2.60	0.00
ENSG00000184349	EFNA5	0.28	-1.86	0.00
ENSG00000125257	ABCC4	0.38	-1.41	0.00
ENSG00000166741	NNMT	0.43	-1.23	0.00
ENSG00000102271	KLHL4	0.09	-3.48	0.00
ENSG00000147676	MAL2	0.24	-2.03	0.00
ENSG00000080823	MOK	0.32	-1.65	0.00
ENSG00000128849	CGNL1	0.24	-2.07	0.00
ENSG00000183780	SLC35F3	0.15	-2.77	0.00
ENSG00000002587	HS3ST1	0.12	-3.06	0.00
ENSG00000171234	UGT2B7	0.30	-1.76	0.00
ENSG00000151632	AKR1C2	0.28	-1.84	0.00
ENSG00000183098	GPC6	0.31	-1.67	0.00
ENSG00000187244	BCAM	0.15	-2.77	0.00
ENSG00000152767	FARP1	0.37	-1.44	0.00
ENSG00000135919	SERPINE2	0.29	-1.77	0.00
ENSG00000172296	SPTLC3	0.29	-1.78	0.00
ENSG00000187764	SEMA4D	0.40	-1.33	0.00
ENSG00000061918	GUCY1B1	0.20	-2.34	0.00
ENSG00000001617	SEMA3F	0.25	-1.98	0.00
ENSG00000151914	DST	0.38	-1.40	0.00
ENSG00000286546	AL079338	0.25	-1.98	0.00
ENSG00000085563	ABCB1	0.21	-2.29	0.00
ENSG00000283154	IQCJ-SCHIP1	0.25	-2.00	0.00
ENSG00000131069	ACSS2	0.33	-1.59	0.00
ENSG00000134986	NREP	0.37	-1.44	0.00
ENSG00000166025	AMOTL1	0.41	-1.30	0.00

ENSG00000261115	TMEM178B	0.37	-1.44	0.00
ENSG00000243649	CFB	0.41	-1.28	0.00
ENSG00000157483	MYO1E	0.44	-1.20	0.00
ENSG00000125848	FLRT3	0.11	-3.19	0.00
ENSG00000186340	THBS2	0.44	-1.18	0.00
ENSG00000131941	RHPN2	0.38	-1.41	0.00
ENSG00000115306	SPTBN1	0.52	-0.94	0.00
ENSG00000157110	RBPMS	0.44	-1.19	0.00
ENSG00000132470	ITGB4	0.19	-2.40	0.00
ENSG00000124564	SLC17A3	0.12	-3.01	0.00
ENSG00000011028	MRC2	0.51	-0.96	0.00
ENSG00000115738	ID2	0.29	-1.78	0.00
ENSG00000117519	CNN3	0.43	-1.20	0.00
ENSG00000107282	APBA1	0.09	-3.48	0.00
ENSG00000147027	TMEM47	0.29	-1.80	0.00
ENSG00000101335	MYL9	0.48	-1.05	0.00
ENSG00000108639	SYNGR2	0.51	-0.98	0.00
ENSG00000100065	CARD10	0.33	-1.60	0.00
ENSG00000101849	TBL1X	0.44	-1.20	0.00
ENSG00000144810	COL8A1	0.27	-1.89	0.00
ENSG00000139998	RAB15	0.27	-1.89	0.00
ENSG00000101654	RNMT	0.42	-1.24	0.00
ENSG00000139734	DIAPH3	0.35	-1.52	0.00
ENSG00000173530	TNFRSF10D	0.38	-1.38	0.00
ENSG00000180914	OXTR	0.27	-1.91	0.00
ENSG00000160867	FGFR4	0.42	-1.26	0.00
ENSG00000099194	SCD	0.49	-1.04	0.00
ENSG00000105976	MET	0.49	-1.02	0.00
ENSG00000124006	OBSL1	0.34	-1.58	0.00
ENSG00000134369	NAV1	0.51	-0.98	0.00
ENSG00000261298	AC122134	0.14	-2.79	0.00
ENSG00000117152	RGS4	0.08	-3.67	0.00
ENSG00000159335	PTMS	0.44	-1.18	0.00
ENSG00000152078	TLCD4	0.18	-2.47	0.00
ENSG00000111344	RASAL1	0.11	-3.22	0.00
ENSG00000131094	C1QL1	0.26	-1.94	0.00
ENSG00000119403	PHF19	0.46	-1.13	0.00
ENSG00000172915	NBEA	0.28	-1.83	0.00
ENSG00000140416	TPM1	0.40	-1.34	0.00
ENSG00000115419	GLS	0.53	-0.91	0.00
ENSG00000137809	ITGA11	0.13	-2.94	0.00
ENSG00000106688	SLC1A1	0.23	-2.15	0.00
ENSG00000165092	ALDH1A1	0.48	-1.07	0.00
ENSG00000182197	EXT1	0.51	-0.97	0.00
ENSG00000091972	CD200	0.07	-3.85	0.00
ENSG00000206053	JPT2	0.48	-1.06	0.00
ENSG00000127863	TNFRSF19	0.07	-3.93	0.00
ENSG00000162433	AK4	0.42	-1.26	0.00
ENSG00000176842	IRX5	0.26	-1.96	0.00
ENSG00000094916	CBX5	0.52	-0.93	0.00
ENSG00000128973	CLN6	0.47	-1.08	0.00
ENSG00000131018	SYNE1	0.38	-1.40	0.00
ENSG00000137807	KIF23	0.41	-1.29	0.00
ENSG00000164574	GALNT10	0.40	-1.31	0.00
ENSG00000177721	ANXA2R	0.17	-2.55	0.00
ENSG00000137819	PAQR5	0.22	-2.20	0.00
ENSG00000140443	IGF1R	0.48	-1.07	0.00
ENSG00000091136	LAMB1	0.56	-0.85	0.00
ENSG00000137310	TCF19	0.41	-1.27	0.00
ENSG00000162631	NTNG1	0.36	-1.48	0.00
ENSG00000077420	APBB1IP	0.48	-1.06	0.00

ENSG00000112246	SIM1	0.22	-2.19	0.00
ENSG00000018408	WWTR1	0.47	-1.09	0.00
ENSG00000174080	CTSF	0.35	-1.51	0.00
ENSG00000000971	CFH	0.36	-1.49	0.00
ENSG00000186566	GPATCH8	0.45	-1.16	0.00
ENSG00000080493	SLC4A4	0.34	-1.55	0.00
ENSG00000169504	CLIC4	0.52	-0.93	0.00
ENSG00000123080	CDKN2C	0.46	-1.12	0.00
ENSG00000137449	CPEB2	0.35	-1.51	0.00
ENSG00000163762	TM4SF18	0.48	-1.07	0.00
ENSG00000173402	DAG1	0.54	-0.88	0.00
ENSG00000005884	ITGA3	0.56	-0.83	0.00
ENSG00000152894	PTPRK	0.42	-1.25	0.00
ENSG00000092964	DPYSL2	0.51	-0.97	0.00
ENSG00000166448	TMEM130	0.10	-3.39	0.00
ENSG00000114480	GBE1	0.51	-0.99	0.00
ENSG00000167191	GPRC5B	0.41	-1.29	0.00
ENSG00000114346	ECT2	0.46	-1.13	0.00
ENSG00000065328	MCM10	0.44	-1.19	0.00
ENSG00000160094	ZNF362	0.29	-1.76	0.00
ENSG00000145819	ARHGAP26	0.34	-1.57	0.00
ENSG00000156140	ADAMTS3	0.31	-1.68	0.00
ENSG00000008441	NFIX	0.49	-1.02	0.00
ENSG00000114279	FGF12	0.23	-2.14	0.00
ENSG00000134243	SORT1	0.38	-1.40	0.00
ENSG00000040275	SPDL1	0.44	-1.17	0.00
ENSG00000139263	LRIG3	0.38	-1.39	0.00
ENSG00000063180	CA11	0.11	-3.17	0.00
ENSG00000115415	STAT1	0.43	-1.21	0.00
ENSG00000179772	FOXS1	0.05	-4.32	0.00
ENSG00000197444	OGDHL	0.35	-1.52	0.00
ENSG00000182871	COL18A1	0.54	-0.88	0.00
ENSG00000152104	PTPN14	0.57	-0.81	0.00
ENSG00000115252	PDE1A	0.09	-3.49	0.00
ENSG00000115380	EFEMP1	0.28	-1.86	0.00
ENSG00000218336	TENM3	0.39	-1.37	0.00
ENSG00000197457	STMN3	0.16	-2.64	0.00
ENSG00000156218	ADAMTSL3	0.21	-2.28	0.00
ENSG00000143061	IGSF3	0.53	-0.92	0.00
ENSG00000122884	P4HA1	0.51	-0.96	0.00
ENSG00000240694	PNMA2	0.18	-2.48	0.00
ENSG00000114541	FRMD4B	0.16	-2.63	0.00
ENSG00000183856	IQGAP3	0.53	-0.92	0.00
ENSG00000100644	HIF1A	0.51	-0.96	0.00
ENSG00000115414	FN1	0.57	-0.81	0.00
ENSG00000104537	ANXA13	0.03	-4.97	0.00
ENSG00000147862	NFIB	0.47	-1.10	0.00
ENSG00000186185	KIF18B	0.44	-1.17	0.00
ENSG00000241119	UGT1A9	0.26	-1.94	0.00
ENSG00000185104	FAF1	0.49	-1.04	0.00
ENSG00000105429	MEGF8	0.48	-1.06	0.00
ENSG00000154310	TNIK	0.35	-1.53	0.00
ENSG00000188042	ARL4C	0.59	-0.77	0.00
ENSG00000196141	SPATS2L	0.41	-1.27	0.00
ENSG00000104998	IL27RA	0.47	-1.09	0.00
ENSG00000075420	FNDC3B	0.48	-1.06	0.00
ENSG00000178531	CTXN1	0.38	-1.40	0.00
ENSG00000109814	UGDH	0.35	-1.50	0.00
ENSG00000100994	PYGB	0.55	-0.86	0.00
ENSG00000131153	GINS2	0.49	-1.02	0.00
ENSG00000121361	KCNJ8	0.24	-2.08	0.00

ENSG00000151388	ADAMTS12	0.55	-0.87	0.00
ENSG00000167632	TRAPPC9	0.42	-1.27	0.00
ENSG00000116106	EPHA4	0.21	-2.27	0.00
ENSG00000106546	AHR	0.47	-1.08	0.00
ENSG00000079308	TNS1	0.33	-1.59	0.00
ENSG00000237649	KIFC1	0.51	-0.98	0.00
ENSG00000142279	WTIP	0.34	-1.58	0.00
ENSG00000038382	TRIO	0.51	-0.97	0.00
ENSG00000197959	DNM3	0.27	-1.90	0.00
ENSG00000133216	EPHB2	0.59	-0.76	0.00
ENSG00000126878	AIF1L	0.16	-2.64	0.00
ENSG00000162105	SHANK2	0.41	-1.28	0.00
ENSG00000101868	POLA1	0.37	-1.45	0.00
ENSG00000141002	TCF25	0.55	-0.86	0.00
ENSG00000131747	TOP2A	0.48	-1.07	0.00
ENSG00000153048	CARHSP1	0.56	-0.83	0.00
ENSG00000079691	CARMIL1	0.46	-1.13	0.00
ENSG00000162522	KIAA1522	0.48	-1.05	0.00
ENSG00000082438	COBLL1	0.27	-1.90	0.00
ENSG00000166106	ADAMTS15	0.04	-4.72	0.00
ENSG00000104765	BNIP3L	0.55	-0.85	0.00
ENSG00000124171	PARD6B	0.29	-1.78	0.00
ENSG00000066279	ASPM	0.41	-1.29	0.00
ENSG00000151640	DPYSL4	0.20	-2.30	0.00
ENSG00000116237	ICMT	0.57	-0.81	0.00
ENSG00000167930	FAM234A	0.53	-0.92	0.00
ENSG00000164638	SLC29A4	0.25	-2.02	0.00
ENSG00000118762	PKD2	0.51	-0.98	0.00
ENSG00000125966	MMP24	0.33	-1.59	0.00
ENSG00000105767	CADM4	0.20	-2.32	0.00
ENSG00000134333	LDHA	0.62	-0.68	0.00
ENSG00000135476	ESPL1	0.46	-1.11	0.00
ENSG00000186918	ZNF395	0.50	-1.01	0.00
ENSG00000082482	KCNK2	0.14	-2.82	0.00
ENSG00000075711	DLG1	0.49	-1.04	0.00
ENSG00000152256	PDK1	0.46	-1.13	0.00
ENSG00000136636	KCTD3	0.55	-0.86	0.00
ENSG00000163781	TOPBP1	0.54	-0.90	0.00
ENSG00000132561	MATN2	0.30	-1.76	0.00
ENSG00000166589	CDH16	0.15	-2.72	0.00
ENSG00000121152	NCAPH	0.50	-0.99	0.00
ENSG00000183444	OR7E38P	0.15	-2.78	0.00
ENSG00000143228	NUF2	0.43	-1.22	0.00
ENSG00000117724	CENPF	0.46	-1.14	0.00
ENSG00000132182	NUP210	0.54	-0.90	0.00
ENSG00000067167	TRAM1	0.54	-0.88	0.00
ENSG00000171298	GAA	0.52	-0.95	0.00
ENSG00000136156	ITM2B	0.60	-0.74	0.00
ENSG00000134013	LOXL2	0.60	-0.73	0.00
ENSG00000185090	MANEAL	0.27	-1.88	0.00
ENSG00000171791	BCL2	0.17	-2.59	0.00
ENSG00000080986	NDC80	0.43	-1.21	0.00
ENSG00000124568	SLC17A1	0.04	-4.61	0.00
ENSG00000120802	TMPO	0.57	-0.82	0.00
ENSG00000071242	RPS6KA2	0.40	-1.33	0.00
ENSG00000138032	PPM1B	0.47	-1.09	0.00
ENSG00000164050	PLXNB1	0.39	-1.35	0.00
ENSG00000126603	GLIS2	0.49	-1.02	0.00
ENSG00000166224	SGPL1	0.56	-0.84	0.00
ENSG00000130508	PXDN	0.60	-0.73	0.00
ENSG00000166033	HTRA1	0.56	-0.84	0.00

ENSG00000233237	LINC00472	0.48	-1.07	0.00
ENSG00000134690	CDCA8	0.54	-0.90	0.00
ENSG00000170876	TMEM43	0.57	-0.81	0.00
ENSG00000152527	PLEKHH2	0.22	-2.18	0.00
ENSG00000144554	FANCD2	0.46	-1.13	0.00
ENSG00000154734	ADAMTS1	0.55	-0.87	0.00
ENSG00000165626	BEND7	0.58	-0.78	0.00
ENSG00000205403	CFI	0.16	-2.63	0.00
ENSG00000070159	PTPN3	0.55	-0.87	0.00
ENSG00000183255	PTTG1IP	0.61	-0.71	0.00
ENSG00000104419	NDRG1	0.59	-0.77	0.00
ENSG00000170421	KRT8	0.61	-0.70	0.00
ENSG00000129173	E2F8	0.29	-1.78	0.00
ENSG00000198053	SIRPA	0.57	-0.81	0.00
ENSG00000132849	PATJ	0.41	-1.29	0.00
ENSG00000082497	SERTAD4	0.43	-1.22	0.00
ENSG00000179862	CITED4	0.47	-1.09	0.00
ENSG00000165102	HGSNAT	0.58	-0.79	0.00
ENSG00000196670	ZFP62	0.17	-2.58	0.00
ENSG00000118007	STAG1	0.51	-0.96	0.00
ENSG00000163359	COL6A3	0.52	-0.93	0.00
ENSG00000154175	ABI3BP	0.42	-1.24	0.00
ENSG00000130193	THEM6	0.45	-1.14	0.00
ENSG00000184897	H1-10	0.48	-1.07	0.00
ENSG00000196975	ANXA4	0.61	-0.71	0.00
ENSG00000145715	RASA1	0.54	-0.88	0.00
ENSG00000187634	SAMD11	0.08	-3.70	0.00
ENSG00000170044	ZPLD1	0.13	-2.96	0.00
ENSG00000073350	LLGL2	0.15	-2.76	0.00
ENSG00000172380	GNG12	0.57	-0.81	0.00
ENSG00000110328	GALNT18	0.34	-1.57	0.00
ENSG00000164116	GUCY1A1	0.14	-2.79	0.00
ENSG00000115935	WIPF1	0.48	-1.06	0.00
ENSG00000112984	KIF20A	0.57	-0.82	0.00
ENSG00000235142	LINC02532	0.01	-7.19	0.00
ENSG00000124766	SOX4	0.57	-0.80	0.00
ENSG00000131067	GGT7	0.41	-1.30	0.00
ENSG00000113657	DPYSL3	0.62	-0.69	0.00
ENSG00000175215	CTDSP2	0.62	-0.69	0.00
ENSG00000197249	SERPINA1	0.28	-1.83	0.00
ENSG00000142303	ADAMTS10	0.29	-1.78	0.00
ENSG00000115902	SLC1A4	0.42	-1.26	0.00
ENSG00000137673	MMP7	0.16	-2.64	0.00
ENSG00000076003	MCM6	0.57	-0.82	0.00
ENSG00000205336	ADGRG1	0.39	-1.34	0.00
ENSG00000131981	LGALS3	0.57	-0.82	0.00
ENSG00000275342	PRAG1	0.44	-1.18	0.00
ENSG00000173068	BNC2	0.40	-1.33	0.00
ENSG00000118495	PLAGL1	0.44	-1.19	0.00
ENSG00000058804	NDC1	0.56	-0.84	0.00
ENSG00000108175	ZMIZ1	0.60	-0.74	0.00
ENSG00000173334	TRIB1	0.54	-0.89	0.00
ENSG00000183248	PRR36	0.20	-2.34	0.00
ENSG00000107331	ABCA2	0.52	-0.93	0.00
ENSG00000169432	SCN9A	0.14	-2.81	0.00
ENSG00000100811	YY1	0.58	-0.79	0.00
ENSG00000013619	MAMLD1	0.35	-1.51	0.00
ENSG00000153395	LPCAT1	0.57	-0.80	0.00
ENSG00000197226	TBC1D9B	0.60	-0.73	0.00
ENSG00000163584	RPL22L1	0.44	-1.18	0.00
ENSG00000146477	SLC22A3	0.26	-1.93	0.00

ENSG00000206503	HLA-A	0.56	-0.83	0.00
ENSG00000176171	BNIP3	0.62	-0.69	0.00
ENSG00000132326	PER2	0.34	-1.55	0.00
ENSG00000141959	PFKL	0.57	-0.81	0.00
ENSG00000143653	SCCPDH	0.60	-0.73	0.00
ENSG00000008710	PKD1	0.50	-0.99	0.00
ENSG00000213123	TCTEX1D2	0.29	-1.77	0.00
ENSG00000106012	IQCE	0.34	-1.58	0.00
ENSG00000116044	NFE2L2	0.54	-0.88	0.00
ENSG00000121039	RDH10	0.52	-0.93	0.00
ENSG00000115109	EPB41L5	0.35	-1.52	0.00
ENSG00000182718	ANXA2	0.59	-0.75	0.00
ENSG00000147852	VLDLR	0.47	-1.09	0.00
ENSG00000160299	PCNT	0.53	-0.91	0.00
ENSG00000178401	DNAJC22	0.35	-1.50	0.00
ENSG00000184445	KNTC1	0.47	-1.08	0.00
ENSG00000077684	JADE1	0.47	-1.09	0.00
ENSG00000088325	TPX2	0.63	-0.67	0.00
ENSG00000035403	VCL	0.65	-0.63	0.00
ENSG00000047648	ARHGAP6	0.13	-2.94	0.00
ENSG00000072501	SMC1A	0.63	-0.67	0.00
ENSG00000158856	DMTN	0.58	-0.79	0.00
ENSG00000086758	HUWE1	0.63	-0.66	0.00
ENSG00000157514	TSC22D3	0.48	-1.05	0.00
ENSG00000149503	INCENP	0.55	-0.87	0.00
ENSG00000149571	KIRREL3	0.13	-2.98	0.00
ENSG00000147324	MFHAS1	0.55	-0.87	0.00
ENSG00000184661	CDCA2	0.50	-1.01	0.00
ENSG00000106462	EZH2	0.55	-0.85	0.00
ENSG00000135636	DYSF	0.21	-2.26	0.00
ENSG00000049239	H6PD	0.61	-0.72	0.00
ENSG00000175970	UNC119B	0.61	-0.71	0.00
ENSG00000141858	SAMD1	0.56	-0.83	0.00
ENSG00000198535	C2CD4A	0.01	-6.69	0.00
ENSG00000153721	CNKSR3	0.41	-1.29	0.00
ENSG00000173706	HEG1	0.58	-0.78	0.00
ENSG00000141738	GRB7	0.30	-1.73	0.00
ENSG00000075539	FRYL	0.55	-0.85	0.00
ENSG00000145431	PDGFC	0.52	-0.94	0.00
ENSG00000058091	CDK14	0.47	-1.08	0.00
ENSG00000149591	TAGLN	0.38	-1.38	0.00
ENSG00000101265	RASSF2	0.51	-0.96	0.00
ENSG00000137942	FNBP1L	0.46	-1.11	0.00
ENSG00000236039	AC019117	0.14	-2.81	0.00
ENSG00000143847	PPFIA4	0.22	-2.18	0.00
ENSG00000145687	SSBP2	0.37	-1.44	0.00
ENSG00000177303	CASKIN2	0.49	-1.02	0.00
ENSG00000007968	E2F2	0.31	-1.70	0.00
ENSG00000138448	ITGAV	0.59	-0.76	0.00
ENSG00000133135	RNF128	0.53	-0.91	0.00
ENSG00000170312	CDK1	0.55	-0.86	0.00
ENSG00000168140	VASN	0.43	-1.21	0.00
ENSG00000049130	KITLG	0.41	-1.30	0.00
ENSG00000067606	PRKCZ	0.20	-2.34	0.00
ENSG00000187955	COL14A1	0.33	-1.58	0.00
ENSG00000197157	SND1	0.65	-0.63	0.00
ENSG00000260927	AC009107	0.09	-3.42	0.00
ENSG00000105486	LIG1	0.56	-0.84	0.00
ENSG00000066629	EML1	0.55	-0.85	0.00
ENSG00000146592	CREB5	0.55	-0.87	0.00
ENSG00000162591	MEGF6	0.06	-4.01	0.00

ENSG00000111276	CDKN1B	0.54	-0.90	0.00
ENSG00000100154	TTC28	0.49	-1.01	0.00
ENSG00000087274	ADD1	0.64	-0.65	0.00
ENSG00000180263	FGD6	0.59	-0.77	0.00
ENSG00000187741	FANCA	0.54	-0.89	0.00
ENSG00000174332	GLIS1	0.44	-1.19	0.00
ENSG00000157985	AGAP1	0.60	-0.75	0.00
ENSG00000164128	NPY1R	0.30	-1.73	0.00
ENSG00000108924	HLF	0.36	-1.48	0.00
ENSG00000205542	TMSB4X	0.67	-0.58	0.00
ENSG00000147394	ZNF185	0.34	-1.57	0.00
ENSG00000198589	LRBA	0.57	-0.80	0.00
ENSG00000151474	FRMD4A	0.61	-0.71	0.00
ENSG00000111057	KRT18	0.53	-0.93	0.00
ENSG00000139266	MARCHF9	0.30	-1.72	0.00
ENSG00000109107	ALDOC	0.46	-1.14	0.00
ENSG00000108846	ABCC3	0.65	-0.63	0.00
ENSG00000132394	EEFSEC	0.50	-1.00	0.00
ENSG00000185483	ROR1	0.30	-1.73	0.00
ENSG00000173517	PEAK1	0.54	-0.88	0.00
ENSG00000079616	KIF22	0.60	-0.73	0.00
ENSG00000105974	CAV1	0.58	-0.78	0.00
ENSG00000142197	DOP1B	0.61	-0.71	0.00
ENSG00000128272	ATF4	0.57	-0.81	0.00
ENSG00000253729	PRKDC	0.67	-0.58	0.00
ENSG00000160145	KALRN	0.42	-1.26	0.00
ENSG00000166508	MCM7	0.64	-0.65	0.00
ENSG00000177084	POLE	0.55	-0.85	0.00
ENSG00000077063	CTTNBP2	0.27	-1.91	0.00
ENSG00000180340	FZD2	0.61	-0.71	0.00
ENSG00000160949	TONSL	0.57	-0.80	0.00
ENSG00000058056	USP13	0.61	-0.71	0.00
ENSG00000105088	OLFM2	0.23	-2.10	0.00
ENSG00000163683	SMIM14	0.53	-0.91	0.00
ENSG00000143878	RHOB	0.59	-0.77	0.00
ENSG00000066933	MYO9A	0.52	-0.95	0.00
ENSG00000113810	SMC4	0.48	-1.05	0.00
ENSG00000286190	AC055839	0.24	-2.03	0.00
ENSG00000162613	FUBP1	0.62	-0.68	0.00
ENSG00000173875	ZNF791	0.22	-2.18	0.00
ENSG00000075218	GTSE1	0.51	-0.98	0.00
ENSG00000165527	ARF6	0.61	-0.71	0.00
ENSG00000140396	NCOA2	0.56	-0.84	0.00
ENSG00000168778	TCTN2	0.49	-1.03	0.00
ENSG00000178202	POGLUT3	0.55	-0.85	0.00
ENSG00000004660	CAMKK1	0.57	-0.82	0.00
ENSG00000100106	TRIOBP	0.55	-0.85	0.00
ENSG00000141736	ERBB2	0.56	-0.85	0.00
ENSG00000134294	SLC38A2	0.60	-0.75	0.00
ENSG00000156639	ZFAND3	0.59	-0.76	0.00
ENSG00000010803	SCMH1	0.38	-1.40	0.00
ENSG00000168056	LTBP3	0.58	-0.79	0.00
ENSG00000119333	WDR34	0.54	-0.88	0.00
ENSG00000162607	USP1	0.59	-0.75	0.00
ENSG00000143845	ETNK2	0.30	-1.73	0.00
ENSG00000269378	AC022149	0.57	-0.81	0.00
ENSG00000171843	MLLT3	0.48	-1.05	0.00
ENSG00000174891	RSRC1	0.51	-0.98	0.00
ENSG00000138160	KIF11	0.56	-0.83	0.00
ENSG00000109586	GALNT7	0.57	-0.82	0.00
ENSG00000224389	C4B	0.10	-3.36	0.00

ENSG00000163535	SGO2	0.41	-1.30	0.00
ENSG00000154080	CHST9	0.23	-2.09	0.00
ENSG00000132549	VPS13B	0.53	-0.91	0.00
ENSG00000234912	SNHG20	0.40	-1.33	0.00
ENSG00000146648	EGFR	0.66	-0.59	0.00
ENSG00000101935	AMMECR1	0.61	-0.72	0.00
ENSG00000113721	PDGFRB	0.43	-1.20	0.00
ENSG00000258947	TUBB3	0.59	-0.77	0.00
ENSG00000158352	SHROOM4	0.42	-1.24	0.00
ENSG00000135862	LAMC1	0.69	-0.54	0.00
ENSG00000182132	KCNIP1	0.10	-3.37	0.00
ENSG00000171564	FGB	0.00	-12.56	0.00
ENSG00000054277	OPN3	0.46	-1.13	0.00
ENSG00000171388	APLN	0.09	-3.41	0.00
ENSG00000146670	CDCA5	0.61	-0.71	0.00
ENSG00000187720	THSD4	0.52	-0.93	0.00
ENSG00000181894	ZNF329	0.27	-1.90	0.00
ENSG00000106714	CNTNAP3	0.20	-2.36	0.00
ENSG00000172716	SLFN11	0.61	-0.70	0.00
ENSG00000118263	KLF7	0.52	-0.94	0.00
ENSG00000154839	SKA1	0.34	-1.57	0.00
ENSG00000163808	KIF15	0.34	-1.57	0.00
ENSG00000165304	MELK	0.54	-0.88	0.00
ENSG00000175643	RMI2	0.48	-1.05	0.00
ENSG00000253661	ZFHX4-AS1	0.20	-2.30	0.00
ENSG00000159788	RGS12	0.55	-0.87	0.00
ENSG00000074054	CLASP1	0.61	-0.71	0.00
ENSG00000177030	DEAF1	0.56	-0.83	0.00
ENSG00000118523	CCN2	0.57	-0.81	0.00
ENSG00000196230	TUBB	0.68	-0.56	0.00
ENSG00000123095	BHLHE41	0.60	-0.74	0.00
ENSG00000154978	VOPP1	0.56	-0.84	0.00
ENSG00000069275	NUCKS1	0.67	-0.58	0.00
ENSG00000182010	RTKN2	0.24	-2.04	0.00
ENSG00000285417	BX571818	0.58	-0.79	0.00
ENSG00000141298	SSH2	0.48	-1.05	0.00
ENSG00000076382	SPAG5	0.60	-0.74	0.00
ENSG00000167972	ABCA3	0.18	-2.44	0.00
ENSG00000073921	PICALM	0.57	-0.80	0.00
ENSG00000109084	TMEM97	0.59	-0.77	0.00
ENSG00000213853	EMP2	0.41	-1.28	0.00
ENSG00000110917	MLEC	0.69	-0.53	0.00
ENSG00000172037	LAMB2	0.60	-0.74	0.00
ENSG00000120875	DUSP4	0.55	-0.85	0.00
ENSG00000167601	AXL	0.66	-0.59	0.00
ENSG00000088812	ATRN	0.62	-0.68	0.00
ENSG00000085999	RAD54L	0.46	-1.13	0.00
ENSG00000214517	PPME1	0.57	-0.82	0.00
ENSG00000114554	PLXNA1	0.65	-0.62	0.00
ENSG00000233491	AC008163	0.12	-3.08	0.00
ENSG00000038945	MSR1	0.34	-1.55	0.00
ENSG00000168575	SLC20A2	0.55	-0.87	0.00
ENSG00000158195	WASF2	0.66	-0.59	0.00
ENSG00000182481	KPNA2	0.69	-0.53	0.00
ENSG00000101447	FAM83D	0.55	-0.85	0.00
ENSG00000122863	CHST3	0.64	-0.65	0.00
ENSG00000258586	LINC02274	0.36	-1.49	0.00
ENSG00000080345	RIF1	0.52	-0.95	0.00
ENSG00000123684	LPGAT1	0.66	-0.60	0.00
ENSG00000104324	CPQ	0.52	-0.95	0.00
ENSG00000118407	FILIP1	0.13	-2.90	0.00

ENSG00000109805	NCAPG	0.53	-0.92	0.00
ENSG00000185585	OLFML2A	0.43	-1.20	0.00
ENSG00000148848	ADAM12	0.52	-0.93	0.00
ENSG00000071894	CPSF1	0.63	-0.67	0.00
ENSG00000116127	ALMS1	0.44	-1.20	0.00
ENSG00000179403	VWA1	0.40	-1.33	0.00
ENSG00000227766	AL671277	0.59	-0.77	0.00
ENSG00000116791	CRYZ	0.59	-0.75	0.00
ENSG00000176890	TYMS	0.53	-0.91	0.00
ENSG00000019485	PRDM11	0.56	-0.84	0.00
ENSG00000162063	CCNF	0.59	-0.76	0.00
ENSG00000162636	FAM102B	0.42	-1.26	0.00
ENSG00000090889	KIF4A	0.65	-0.63	0.00
ENSG00000165868	HSPA12A	0.58	-0.79	0.00
ENSG00000115464	USP34	0.56	-0.83	0.00
ENSG00000105971	CAV2	0.66	-0.60	0.00
ENSG00000231991	ANXA2P2	0.56	-0.83	0.00
ENSG00000131558	EXOC4	0.65	-0.61	0.00
ENSG00000072071	ADGRL1	0.29	-1.81	0.00
ENSG00000254300	LINC01111	0.08	-3.63	0.00
ENSG00000112624	BICRAL	0.53	-0.92	0.00
ENSG00000138604	GLCE	0.46	-1.11	0.00
ENSG00000101003	GINS1	0.55	-0.87	0.00
ENSG00000021300	PLEKHB1	0.34	-1.58	0.00
ENSG00000185697	MYBL1	0.44	-1.19	0.00
ENSG00000111371	SLC38A1	0.68	-0.56	0.00
ENSG00000234745	HLA-B	0.55	-0.85	0.00
ENSG00000188157	AGRN	0.65	-0.63	0.00
ENSG00000075275	CELSR1	0.61	-0.72	0.00
ENSG00000154127	UBASH3B	0.66	-0.60	0.00
ENSG00000140534	TICRR	0.48	-1.05	0.00
ENSG00000104738	MCM4	0.66	-0.61	0.00
ENSG00000144824	PHLDB2	0.51	-0.99	0.00
ENSG00000107863	ARHGAP21	0.65	-0.62	0.00
ENSG00000150093	ITGB1	0.60	-0.74	0.00
ENSG00000144218	AFF3	0.58	-0.78	0.00
ENSG00000214944	ARHGEF28	0.60	-0.74	0.00
ENSG00000055609	KMT2C	0.62	-0.68	0.00
ENSG00000100557	CCDC198	0.44	-1.20	0.00
ENSG00000118922	KLF12	0.37	-1.44	0.00
ENSG00000176692	FOXC2	0.44	-1.18	0.00
ENSG00000196526	AFAP1	0.61	-0.72	0.00
ENSG00000180815	MAP3K15	0.40	-1.32	0.00
ENSG00000166483	WEE1	0.57	-0.80	0.00
ENSG00000140525	FANCI	0.57	-0.81	0.00
ENSG00000122966	CIT	0.58	-0.78	0.00
ENSG00000143815	LBR	0.65	-0.61	0.00
ENSG00000156802	ATAD2	0.53	-0.93	0.00
ENSG00000092470	WDR76	0.46	-1.13	0.00
ENSG00000139174	PRICKLE1	0.38	-1.40	0.00
ENSG00000118785	SPP1	0.66	-0.60	0.00
ENSG00000125457	MIF4GD	0.48	-1.06	0.00
ENSG00000102189	EEA1	0.54	-0.90	0.00
ENSG00000143248	RGS5	0.33	-1.62	0.00
ENSG00000152253	SPC25	0.54	-0.90	0.00
ENSG00000198838	RYR3	0.22	-2.19	0.00
ENSG00000109794	FAM149A	0.37	-1.45	0.00
ENSG00000124225	PMEPA1	0.64	-0.64	0.00
ENSG00000120685	PROSER1	0.57	-0.82	0.00
ENSG00000285155	AC092153	0.08	-3.69	0.00
ENSG00000141562	NARF	0.63	-0.66	0.00

ENSG00000175567	UCP2	0.44	-1.18	0.00
ENSG00000115310	RTN4	0.71	-0.50	0.00
ENSG00000123989	CHPF	0.61	-0.72	0.00
ENSG00000258366	RTEL1	0.52	-0.95	0.00
ENSG00000137845	ADAM10	0.63	-0.66	0.00
ENSG00000184985	SORCS2	0.58	-0.78	0.00
ENSG00000142599	RERE	0.65	-0.63	0.00
ENSG00000011426	ANLN	0.58	-0.79	0.00
ENSG00000156970	BUB1B	0.59	-0.76	0.00
ENSG00000130827	PLXNA3	0.47	-1.09	0.00
ENSG00000157800	SLC37A3	0.49	-1.04	0.00
ENSG00000168610	STAT3	0.67	-0.59	0.00
ENSG00000174827	PDZK1	0.19	-2.37	0.00
ENSG00000092853	CLSPN	0.55	-0.87	0.00
ENSG00000286070	AP000356	0.01	-6.42	0.00
ENSG00000104341	LAPTM4B	0.71	-0.50	0.00
ENSG00000119280	C1orf198	0.60	-0.73	0.00
ENSG00000144455	SUMF1	0.44	-1.17	0.00
ENSG00000248098	BCKDHA	0.60	-0.74	0.00
ENSG00000163412	EIF4E3	0.21	-2.22	0.00
ENSG00000159216	RUNX1	0.52	-0.94	0.00
ENSG00000168300	PCMTD1	0.48	-1.07	0.00
ENSG00000072134	EPN2	0.50	-1.00	0.00
ENSG00000134198	TSPAN2	0.24	-2.04	0.00
ENSG00000073849	ST6GAL1	0.48	-1.07	0.00
ENSG00000187134	AKR1C1	0.62	-0.70	0.00
ENSG00000153904	DDAH1	0.36	-1.47	0.00
ENSG00000168096	ANKS3	0.56	-0.85	0.00
ENSG00000113441	LNPEP	0.65	-0.62	0.00
ENSG00000171435	KSR2	0.20	-2.34	0.00
ENSG00000047410	TPR	0.62	-0.68	0.00
ENSG00000131378	RFTN1	0.52	-0.95	0.00
ENSG00000184374	COLEC10	0.59	-0.75	0.00
ENSG00000163625	WDFY3	0.55	-0.85	0.00
ENSG00000171848	RRM2	0.66	-0.59	0.00
ENSG00000171603	CLSTN1	0.66	-0.60	0.00
ENSG00000181104	F2R	0.58	-0.79	0.00
ENSG00000105810	CDK6	0.60	-0.74	0.00
ENSG00000112759	SLC29A1	0.52	-0.95	0.00
ENSG00000100228	RAB36	0.51	-0.97	0.00
ENSG00000131779	PEX11B	0.58	-0.79	0.00
ENSG00000189060	H1-0	0.55	-0.85	0.00
ENSG00000107679	PLEKHA1	0.59	-0.76	0.00
ENSG00000138182	KIF20B	0.46	-1.12	0.00
ENSG00000137693	YAP1	0.66	-0.59	0.00
ENSG00000172824	CES4A	0.42	-1.27	0.00
ENSG00000182670	TTC3	0.51	-0.97	0.00
ENSG00000174684	B4GAT1	0.59	-0.76	0.00
ENSG00000130270	ATP8B3	0.41	-1.28	0.00
ENSG00000138449	SLC40A1	0.32	-1.62	0.00
ENSG00000174233	ADCY6	0.54	-0.88	0.00
ENSG00000068078	FGFR3	0.31	-1.69	0.00
ENSG00000109686	SH3D19	0.60	-0.73	0.00
ENSG00000135052	GOLM1	0.69	-0.54	0.00
ENSG00000254536	AL360181	0.27	-1.90	0.00
ENSG00000102007	PLP2	0.69	-0.54	0.00
ENSG00000137770	CTDSPL2	0.57	-0.82	0.00
ENSG00000161921	CXCL16	0.25	-2.00	0.00
ENSG00000102384	CENPI	0.53	-0.91	0.00
ENSG00000003436	TFPI	0.64	-0.66	0.00
ENSG00000173852	DPY19L1	0.63	-0.67	0.00

ENSG00000124440	HIF3A	0.37	-1.44	0.00
ENSG00000128951	DUT	0.58	-0.78	0.00
ENSG00000137831	UACA	0.50	-1.00	0.00
ENSG00000153071	DAB2	0.66	-0.59	0.00
ENSG00000130816	DNMT1	0.67	-0.57	0.00
ENSG00000151490	PTPRO	0.33	-1.59	0.00
ENSG00000269556	TMEM185A	0.58	-0.77	0.00
ENSG00000100196	KDELR3	0.56	-0.83	0.00
ENSG00000147044	CASK	0.63	-0.66	0.00
ENSG00000145386	CCNA2	0.63	-0.66	0.00
ENSG00000177565	TBL1XR1	0.67	-0.58	0.00
ENSG00000106484	MEST	0.16	-2.69	0.00
ENSG00000151725	CENPU	0.49	-1.03	0.00
ENSG00000143157	POGK	0.68	-0.55	0.00
ENSG00000092621	PHGDH	0.66	-0.61	0.00
ENSG00000177728	TMEM94	0.52	-0.94	0.00
ENSG00000099991	CABIN1	0.60	-0.74	0.00
ENSG00000176834	VSIG10	0.62	-0.69	0.00
ENSG00000175063	UBE2C	0.63	-0.67	0.00
ENSG00000120708	TGFBI	0.62	-0.70	0.00
ENSG00000177181	RIMKLA	0.07	-3.87	0.00
ENSG00000157510	AFAP1L1	0.64	-0.65	0.00
ENSG00000080824	HSP90AA1	0.68	-0.56	0.00
ENSG00000111206	FOXM1	0.69	-0.54	0.00
ENSG00000123815	COQ8B	0.55	-0.85	0.00
ENSG00000143776	CDC42BPA	0.62	-0.69	0.00
ENSG00000103811	CTSH	0.59	-0.76	0.00
ENSG00000185480	PARPBP	0.44	-1.20	0.00
ENSG00000151240	DIP2C	0.61	-0.70	0.00
ENSG00000119242	CCDC92	0.63	-0.68	0.00
ENSG00000188486	H2AX	0.67	-0.58	0.00
ENSG00000071967	CYBRD1	0.64	-0.64	0.00
ENSG00000133315	MACROD1	0.44	-1.18	0.00
ENSG00000144560	VGLL4	0.66	-0.60	0.00
ENSG00000128791	TWSG1	0.67	-0.57	0.00
ENSG00000164741	DLC1	0.62	-0.70	0.00
ENSG00000167165	UGT1A6	0.14	-2.79	0.00
ENSG00000100422	CERK	0.66	-0.60	0.00
ENSG00000081692	JMJD4	0.55	-0.86	0.00
ENSG00000017797	RALBP1	0.69	-0.53	0.00
ENSG00000138134	STAMBPL1	0.51	-0.97	0.00
ENSG00000125398	SOX9	0.48	-1.05	0.00
ENSG00000128245	YWHAH	0.70	-0.52	0.00
ENSG00000278259	MYO19	0.66	-0.60	0.00
ENSG00000126787	DLGAP5	0.59	-0.77	0.00
ENSG00000137968	SLC44A5	0.17	-2.57	0.00
ENSG00000117713	ARID1A	0.68	-0.55	0.00
ENSG00000142173	COL6A2	0.67	-0.57	0.00
ENSG00000144724	PTPRG	0.40	-1.31	0.00
ENSG00000138587	MNS1	0.27	-1.90	0.00
ENSG00000125970	RALY	0.67	-0.58	0.00
ENSG00000077585	GPR137B	0.53	-0.92	0.00
ENSG00000139645	ANKRD52	0.64	-0.64	0.00
ENSG00000149929	HIRIP3	0.59	-0.77	0.00
ENSG00000197763	TXNRD3	0.53	-0.93	0.00
ENSG00000173715	C11orf80	0.42	-1.23	0.00
ENSG00000226445	BX322234	0.24	-2.06	0.00
ENSG00000259316	AC087632	0.03	-5.19	0.00
ENSG00000169129	AFAP1L2	0.63	-0.67	0.00
ENSG00000173950	XXYLT1	0.64	-0.64	0.00
ENSG00000154309	DISP1	0.58	-0.80	0.00

ENSG00000185591	SP1	0.69	-0.54	0.00
ENSG00000147274	RBMX	0.67	-0.57	0.00
ENSG00000125386	FAM193A	0.65	-0.62	0.00
ENSG00000170043	TRAPPC1	0.64	-0.64	0.00
ENSG00000110400	NECTIN1	0.45	-1.17	0.00
ENSG00000104881	PPP1R13L	0.56	-0.84	0.00
ENSG00000049618	ARID1B	0.64	-0.64	0.00
ENSG00000129925	PGAP6	0.65	-0.62	0.00
ENSG00000107140	TESK1	0.59	-0.76	0.00
ENSG00000150760	DOCK1	0.65	-0.62	0.00
ENSG00000173930	SLCO4C1	0.09	-3.44	0.00
ENSG00000090863	GLG1	0.68	-0.56	0.00
ENSG00000110693	SOX6	0.18	-2.50	0.00
ENSG00000091409	ITGA6	0.51	-0.98	0.00
ENSG00000117114	ADGRL2	0.40	-1.34	0.00
ENSG00000111087	GLI1	0.13	-2.98	0.00
ENSG00000197747	S100A10	0.72	-0.48	0.00
ENSG00000156103	MMP16	0.50	-0.99	0.00
ENSG00000251432	LINC02615	0.33	-1.62	0.00
ENSG00000102181	CD99L2	0.67	-0.57	0.00
ENSG00000181555	SETD2	0.62	-0.69	0.00
ENSG00000259768	AC004943	0.52	-0.93	0.00
ENSG00000164849	GPR146	0.31	-1.67	0.00
ENSG00000142871	CCN1	0.59	-0.76	0.00
ENSG00000173230	GOLGB1	0.65	-0.62	0.00
ENSG00000169122	FAM110B	0.12	-3.04	0.00
ENSG00000077782	FGFR1	0.49	-1.04	0.00
ENSG00000169418	NPR1	0.32	-1.66	0.00
ENSG00000101464	PIGU	0.59	-0.76	0.00
ENSG00000112294	ALDH5A1	0.57	-0.82	0.00
ENSG00000138185	ENTPD1	0.17	-2.57	0.00
ENSG00000100767	PAPLN	0.14	-2.88	0.00
ENSG00000173456	RNF26	0.67	-0.57	0.00
ENSG00000100297	MCM5	0.62	-0.68	0.00
ENSG00000026950	BTN3A1	0.54	-0.90	0.00
ENSG00000113269	RNF130	0.64	-0.64	0.00
ENSG00000142733	MAP3K6	0.53	-0.92	0.00
ENSG00000044115	CTNNA1	0.67	-0.57	0.00
ENSG00000117632	STMN1	0.57	-0.81	0.00
ENSG00000143147	GPR161	0.62	-0.68	0.00
ENSG00000103502	CDIPT	0.66	-0.60	0.00
ENSG00000178904	DPY19L3	0.49	-1.03	0.00
ENSG00000138246	DNAJC13	0.66	-0.59	0.00
ENSG00000151276	MAGI1	0.53	-0.91	0.00
ENSG00000147162	OGT	0.70	-0.52	0.00
ENSG00000180592	SKIDA1	0.31	-1.69	0.00
ENSG00000162545	CAMK2N1	0.41	-1.29	0.00
ENSG00000128833	MYO5C	0.12	-3.04	0.00
ENSG00000179364	PACS2	0.58	-0.78	0.00
ENSG00000143643	TTC13	0.56	-0.82	0.00
ENSG00000102531	FNDC3A	0.66	-0.59	0.00
ENSG00000154240	CEP112	0.49	-1.04	0.00
ENSG00000137812	KNL1	0.38	-1.39	0.00
ENSG00000137804	NUSAP1	0.62	-0.68	0.00
ENSG00000127415	IDUA	0.50	-1.00	0.00
ENSG00000185347	TEDC1	0.49	-1.04	0.00
ENSG00000117394	SLC2A1	0.54	-0.90	0.00
ENSG00000133812	SBF2	0.59	-0.77	0.00
ENSG00000198914	POU3F3	0.65	-0.63	0.00
ENSG00000197969	VPS13A	0.56	-0.84	0.00
ENSG00000196367	TRRAP	0.69	-0.53	0.00

ENSG00000104723	TUSC3	0.59	-0.76	0.00
ENSG00000135968	GCC2	0.57	-0.81	0.00
ENSG00000100461	RBM23	0.66	-0.60	0.00
ENSG00000112851	ERBIN	0.64	-0.63	0.00
ENSG00000152795	HNRNPDL	0.72	-0.48	0.00
ENSG00000141027	NCOR1	0.68	-0.56	0.00
ENSG00000136492	BRIP1	0.43	-1.22	0.00
ENSG00000138778	CENPE	0.40	-1.32	0.00
ENSG00000185950	IRS2	0.65	-0.61	0.00
ENSG00000179241	LDLRAD3	0.60	-0.74	0.00
ENSG00000150630	VEGFC	0.67	-0.57	0.00
ENSG00000048471	SNX29	0.63	-0.67	0.00
ENSG00000138759	FRAS1	0.33	-1.59	0.00
ENSG00000183049	CAMK1D	0.61	-0.71	0.00
ENSG00000139613	SMARCC2	0.72	-0.48	0.00
ENSG00000215114	UBXN2B	0.65	-0.63	0.00
ENSG00000186871	ERCC6L	0.54	-0.88	0.00
ENSG00000127564	PKMYT1	0.58	-0.79	0.00
ENSG00000111877	MCM9	0.46	-1.14	0.00
ENSG00000164830	OXR1	0.55	-0.86	0.00
ENSG00000125107	CNOT1	0.71	-0.50	0.00
ENSG00000131873	CHSY1	0.67	-0.59	0.00
ENSG00000205476	CCDC85C	0.51	-0.97	0.00
ENSG00000071564	TCF3	0.66	-0.61	0.00
ENSG00000284118	MIR4707	0.00	-17.49	0.00
ENSG00000138079	SLC3A1	0.10	-3.26	0.00
ENSG00000142687	KIAA0319L	0.68	-0.55	0.00
ENSG00000121892	PDS5A	0.66	-0.60	0.00
ENSG00000000003	TSPAN6	0.63	-0.67	0.00
ENSG00000128191	DGCR8	0.64	-0.65	0.00
ENSG00000112118	MCM3	0.59	-0.76	0.00
ENSG00000159128	IFNGR2	0.61	-0.71	0.00
ENSG00000256043	CTSO	0.60	-0.73	0.00
ENSG00000077549	CAPZB	0.70	-0.51	0.00
ENSG00000184083	FAM120C	0.47	-1.09	0.00
ENSG00000276107	AC037198	0.00	-13.70	0.00
ENSG00000159403	C1R	0.57	-0.81	0.00
ENSG00000225663	MCRIP1	0.62	-0.69	0.00
ENSG00000100714	MTHFD1	0.71	-0.49	0.00
ENSG00000257923	CUX1	0.69	-0.53	0.00
ENSG00000242110	AMACR	0.55	-0.86	0.00
ENSG00000185909	KLHDC8B	0.45	-1.15	0.00
ENSG00000250899	AC125807	0.53	-0.90	0.00
ENSG00000130024	PHF10	0.57	-0.82	0.00
ENSG00000198901	PRC1	0.71	-0.50	0.00
ENSG00000168785	TSPAN5	0.59	-0.76	0.00
ENSG00000159267	HLCS	0.57	-0.80	0.00
ENSG00000205978	NYNRIN	0.45	-1.17	0.00
ENSG00000091622	PITPNM3	0.50	-0.99	0.00
ENSG00000163485	ADORA1	0.67	-0.57	0.00
ENSG00000170017	ALCAM	0.64	-0.65	0.00
ENSG00000182544	MFSD5	0.62	-0.70	0.00
ENSG00000120254	MTHFD1L	0.63	-0.66	0.00
ENSG00000101412	E2F1	0.62	-0.68	0.00
ENSG00000253552	HOXA-AS2	0.42	-1.24	0.00
ENSG00000110108	TMEM109	0.70	-0.51	0.00
ENSG00000276851	AC002401	0.33	-1.59	0.00
ENSG00000105854	PON2	0.67	-0.58	0.00
ENSG00000110987	BCL7A	0.45	-1.16	0.00
ENSG00000134533	RERG	0.13	-2.96	0.00
ENSG00000106804	C5	0.21	-2.28	0.00

ENSG00000135622	SEMA4F	0.44	-1.18	0.00
ENSG00000178409	BEND3	0.45	-1.14	0.00
ENSG00000142945	KIF2C	0.64	-0.64	0.00
ENSG00000116679	IVNS1ABP	0.59	-0.76	0.00
ENSG00000260032	NORAD	0.72	-0.47	0.00
ENSG00000132842	AP3B1	0.67	-0.58	0.00
ENSG00000153956	CACNA2D1	0.57	-0.82	0.00
ENSG00000185650	ZFP36L1	0.72	-0.47	0.00
ENSG00000170558	CDH2	0.58	-0.78	0.00
ENSG00000196411	EPHB4	0.61	-0.72	0.00
ENSG00000087266	SH3BP2	0.69	-0.54	0.00
ENSG00000071282	LMCD1	0.52	-0.95	0.00
ENSG00000204634	TBC1D8	0.53	-0.90	0.00
ENSG00000074047	GLI2	0.42	-1.26	0.00
ENSG00000146247	PHIP	0.60	-0.75	0.00
ENSG00000079246	XRCC5	0.65	-0.61	0.00
ENSG00000047849	MAP4	0.72	-0.48	0.00
ENSG00000151746	BICD1	0.58	-0.78	0.00
ENSG00000104728	ARHGEF10	0.67	-0.57	0.00
ENSG00000116117	PARD3B	0.43	-1.23	0.00
ENSG00000168763	CNNM3	0.61	-0.71	0.00
ENSG00000100596	SPTLC2	0.65	-0.62	0.00
ENSG00000261796	ISY1-RAB43	0.00	-13.50	0.00
ENSG00000151414	NEK7	0.61	-0.72	0.00
ENSG00000198554	WDHD1	0.53	-0.92	0.00
ENSG00000156113	KCNMA1	0.66	-0.60	0.00
ENSG00000170949	ZNF160	0.44	-1.20	0.00
ENSG00000158169	FANCC	0.59	-0.75	0.00
ENSG00000183723	CMTM4	0.45	-1.16	0.00
ENSG00000167670	CHAF1A	0.67	-0.59	0.00
ENSG00000188641	DPYD	0.46	-1.12	0.00
ENSG00000067141	NEO1	0.65	-0.62	0.00
ENSG00000000460	C1orf112	0.62	-0.69	0.00
ENSG00000127328	RAB3IP	0.42	-1.24	0.00
ENSG00000111799	COL12A1	0.46	-1.11	0.00
ENSG00000115461	IGFBP5	0.27	-1.89	0.00
ENSG00000115364	MRPL19	0.53	-0.91	0.00
ENSG00000164761	TNFRSF11B	0.50	-0.99	0.00
ENSG00000281332	LINC00997	0.31	-1.67	0.00
ENSG00000100345	MYH9	0.74	-0.43	0.00
ENSG00000126777	KTN1	0.69	-0.54	0.00
ENSG00000145996	CDKAL1	0.55	-0.86	0.00
ENSG00000278540	ACACA	0.69	-0.54	0.00
ENSG00000184162	NR2C2AP	0.58	-0.77	0.00
ENSG00000158106	RHPN1	0.43	-1.22	0.00
ENSG00000170776	AKAP13	0.70	-0.51	0.00
ENSG00000130768	SMPDL3B	0.47	-1.10	0.00
ENSG00000077514	POLD3	0.66	-0.60	0.00
ENSG00000101680	LAMA1	0.73	-0.46	0.00
ENSG00000115970	THADA	0.66	-0.61	0.00
ENSG00000166783	MARF1	0.68	-0.55	0.00
ENSG00000214193	SH3D21	0.57	-0.80	0.00
ENSG00000159792	PSKH1	0.72	-0.48	0.00
ENSG00000165185	KIAA1958	0.43	-1.21	0.00
ENSG00000112029	FBXO5	0.52	-0.94	0.00
ENSG00000134884	ARGLU1	0.71	-0.50	0.00
ENSG00000189362	NEMP2	0.57	-0.81	0.00
ENSG00000005812	FBXL3	0.63	-0.67	0.00
ENSG00000174483	BBS1	0.47	-1.09	0.00
ENSG00000077232	DNAJC10	0.70	-0.52	0.00
ENSG00000082805	ERC1	0.58	-0.78	0.00

ENSG00000196935	SRGAP1	0.57	-0.80	0.00
ENSG00000132589	FLOT2	0.61	-0.71	0.00
ENSG00000139641	ESYT1	0.74	-0.43	0.00
ENSG00000144283	PKP4	0.65	-0.62	0.00
ENSG00000156398	SFXN2	0.59	-0.75	0.00
ENSG00000141458	NPC1	0.70	-0.51	0.00
ENSG00000189057	FAM111B	0.39	-1.35	0.00
ENSG00000248187	AC078850	0.62	-0.70	0.00
ENSG00000165480	SKA3	0.55	-0.85	0.00
ENSG00000134644	PUM1	0.64	-0.64	0.00
ENSG00000169607	CKAP2L	0.56	-0.84	0.00
ENSG00000140848	CPNE2	0.66	-0.59	0.00
ENSG00000019186	CYP24A1	0.69	-0.54	0.00
ENSG00000163516	ANKZF1	0.64	-0.64	0.00
ENSG00000105426	PTPRS	0.63	-0.66	0.00
ENSG00000181625	SLX1B	0.60	-0.75	0.00
ENSG00000197702	PARVA	0.73	-0.45	0.00
ENSG00000180801	ARSJ	0.62	-0.68	0.00
ENSG00000174428	GTF2IRD2B	0.54	-0.90	0.00
ENSG00000176903	PNMA1	0.71	-0.50	0.00
ENSG00000162714	ZNF496	0.71	-0.49	0.00
ENSG00000141376	BCAS3	0.61	-0.71	0.00
ENSG00000079112	CDH17	0.29	-1.80	0.00
ENSG00000065809	FAM107B	0.56	-0.83	0.00
ENSG00000078674	PCM1	0.58	-0.80	0.00
ENSG00000215252	GOLGA8B	0.64	-0.64	0.00
ENSG00000138796	HADH	0.69	-0.54	0.00
ENSG00000003249	DBNDD1	0.46	-1.12	0.00
ENSG00000111684	LPCAT3	0.57	-0.80	0.00
ENSG00000185499	MUC1	0.27	-1.86	0.00
ENSG00000013810	TACC3	0.59	-0.77	0.00
ENSG00000178764	ZHX2	0.60	-0.73	0.00
ENSG00000112379	ARFGEF3	0.49	-1.03	0.00
ENSG00000139618	BRCA2	0.46	-1.12	0.00
ENSG00000170571	EMB	0.58	-0.77	0.00
ENSG00000152558	TMEM123	0.69	-0.53	0.00
ENSG00000021645	NRXN3	0.02	-5.44	0.00
ENSG00000108784	NAGLU	0.69	-0.53	0.00
ENSG00000146955	RAB19	0.08	-3.72	0.00
ENSG00000067182	TNFRSF1A	0.74	-0.44	0.00
ENSG00000067715	SYT1	0.44	-1.19	0.00
ENSG00000244274	DBNDD2	0.33	-1.61	0.00
ENSG00000170961	HAS2	0.27	-1.91	0.00
ENSG00000161395	PGAP3	0.57	-0.82	0.00
ENSG00000107186	MPDZ	0.65	-0.61	0.00
ENSG00000180011	ZADH2	0.56	-0.83	0.00
ENSG00000134057	CCNB1	0.74	-0.44	0.00
ENSG00000263001	GTF2I	0.64	-0.65	0.00
ENSG00000120837	NFYB	0.61	-0.72	0.00
ENSG00000102606	ARHGEF7	0.70	-0.51	0.00
ENSG00000112312	GMNN	0.61	-0.71	0.00
ENSG00000276043	UHRF1	0.59	-0.77	0.00
ENSG00000131652	THOC6	0.61	-0.72	0.00
ENSG00000163576	EFHB	0.16	-2.68	0.00
ENSG00000084774	CAD	0.71	-0.49	0.00
ENSG00000158470	B4GALT5	0.73	-0.45	0.00
ENSG00000246859	STARD4-AS1	0.39	-1.34	0.00
ENSG00000094841	UPRT	0.47	-1.10	0.00
ENSG00000151364	KCTD14	0.45	-1.16	0.00
ENSG00000164040	PGRMC2	0.23	-2.10	0.00
ENSG00000031003	FAM13B	0.63	-0.66	0.00

ENSG00000100302	RASD2	0.05	-4.30	0.00
ENSG00000068024	HDAC4	0.63	-0.67	0.00
ENSG00000165244	ZNF367	0.45	-1.15	0.00
ENSG00000142192	APP	0.71	-0.50	0.00
ENSG00000089006	SNX5	0.71	-0.49	0.00
ENSG00000153086	ACMSD	0.22	-2.19	0.00
ENSG00000133119	RFC3	0.65	-0.62	0.00
ENSG00000141560	FN3KRP	0.71	-0.49	0.00
ENSG00000259976	AC093010	0.50	-1.01	0.00
ENSG00000166897	ELFN2	0.61	-0.72	0.00
ENSG00000072778	ACADVL	0.71	-0.50	0.00
ENSG00000002822	MAD1L1	0.56	-0.85	0.00
ENSG00000137522	RNF121	0.55	-0.87	0.00
ENSG00000196313	POM121	0.73	-0.45	0.00
ENSG00000271383	NBPF19	0.54	-0.89	0.00
ENSG00000103227	LMF1	0.33	-1.61	0.00
ENSG00000175727	MLXIP	0.74	-0.43	0.00
ENSG00000072274	TFRC	0.74	-0.44	0.00
ENSG00000204685	STARD7-AS1	0.33	-1.61	0.00
ENSG00000249115	HAUS5	0.57	-0.82	0.00
ENSG00000072571	HMMR	0.64	-0.64	0.00
ENSG00000172830	SSH3	0.65	-0.61	0.00
ENSG00000133026	MYH10	0.74	-0.44	0.00
ENSG00000167123	CERCAM	0.55	-0.87	0.00
ENSG00000113368	LMNB1	0.57	-0.81	0.00
ENSG00000149218	ENDOD1	0.57	-0.81	0.00
ENSG00000104320	NBN	0.68	-0.56	0.00
ENSG00000187210	GCNT1	0.40	-1.33	0.00
ENSG00000112033	PPARD	0.69	-0.54	0.00
ENSG00000113163	CERT1	0.63	-0.67	0.00
ENSG00000124942	AHNAK	0.66	-0.61	0.00
ENSG00000153317	ASAP1	0.69	-0.53	0.00
ENSG00000106609	TMEM248	0.72	-0.48	0.00
ENSG00000164985	PSIP1	0.68	-0.55	0.00
ENSG00000146918	NCAPG2	0.73	-0.46	0.00
ENSG00000157837	SPPL3	0.68	-0.56	0.00
ENSG00000159433	STARD9	0.56	-0.83	0.00
ENSG00000114631	PODXL2	0.70	-0.51	0.00
ENSG00000170482	SLC23A1	0.30	-1.76	0.00
ENSG00000197299	BLM	0.51	-0.98	0.00
ENSG00000149639	SOGA1	0.72	-0.47	0.00
ENSG00000261051	AC107021	0.13	-2.93	0.00
ENSG00000104267	CA2	0.71	-0.49	0.00
ENSG00000088756	ARHGAP28	0.36	-1.46	0.00
ENSG00000140694	PARN	0.66	-0.59	0.00
ENSG00000138190	EXOC6	0.48	-1.06	0.00
ENSG00000067082	KLF6	0.72	-0.47	0.00
ENSG00000101439	CST3	0.64	-0.64	0.00
ENSG00000134709	HOOK1	0.27	-1.88	0.00
ENSG00000180901	KCTD2	0.68	-0.55	0.00
ENSG00000196968	FUT11	0.57	-0.82	0.00
ENSG00000100201	DDX17	0.76	-0.39	0.00
ENSG00000196584	XRCC2	0.66	-0.61	0.00
ENSG00000154358	OBSCN	0.57	-0.80	0.00
ENSG00000164808	SPIDR	0.63	-0.67	0.00
ENSG00000101199	ARFGAP1	0.71	-0.49	0.00
ENSG00000125954	CHURC1-FNTB	0.00	-11.70	0.00
ENSG00000069535	MAOB	0.17	-2.60	0.00
ENSG00000227500	SCAMP4	0.56	-0.83	0.00
ENSG00000140839	CLEC18B	0.06	-3.95	0.00
ENSG00000172175	MALT1	0.60	-0.74	0.00

ENSG00000140743	CDR2	0.66	-0.59	0.00
ENSG00000134352	IL6ST	0.69	-0.53	0.00
ENSG00000154545	MAGED4	0.58	-0.78	0.00
ENSG00000244731	C4A	0.20	-2.34	0.00
ENSG00000103335	PIEZO1	0.68	-0.56	0.00
ENSG00000197905	TEAD4	0.67	-0.57	0.00
ENSG00000141576	RNF157	0.67	-0.58	0.00
ENSG00000137075	RNF38	0.62	-0.69	0.00
ENSG00000165730	STOX1	0.17	-2.53	0.00
ENSG00000044446	PHKA2	0.65	-0.62	0.00
ENSG00000143590	EFNA3	0.38	-1.41	0.00
ENSG00000110719	TCIRG1	0.71	-0.49	0.00
ENSG00000069966	GNB5	0.65	-0.62	0.00
ENSG00000119906	SLF2	0.66	-0.59	0.00
ENSG00000126822	PLEKHG3	0.58	-0.79	0.00
ENSG00000112531	QKI	0.62	-0.69	0.00
ENSG00000119522	DENND1A	0.67	-0.59	0.00
ENSG00000167536	DHRS13	0.53	-0.92	0.00
ENSG00000274070	CASTOR2	0.49	-1.02	0.00
ENSG00000146067	FAM193B	0.62	-0.68	0.00
ENSG00000167513	CDT1	0.65	-0.62	0.00
ENSG00000115073	ACTR1B	0.61	-0.72	0.00
ENSG00000157827	FMNL2	0.72	-0.48	0.00
ENSG00000135423	GLS2	0.01	-6.44	0.00
ENSG00000031823	RANBP3	0.73	-0.46	0.00
ENSG00000070413	DGCR2	0.70	-0.52	0.00
ENSG00000104361	NIPAL2	0.44	-1.18	0.00
ENSG00000235272	RAMACL	0.09	-3.48	0.00
ENSG00000066739	ATG2B	0.59	-0.75	0.00
ENSG00000184640	SEPTIN9	0.73	-0.46	0.00
ENSG00000114670	NEK11	0.45	-1.14	0.00
ENSG00000014138	POLA2	0.67	-0.58	0.00
ENSG00000250634	LINC01182	0.67	-0.57	0.00
ENSG00000155816	FMN2	0.37	-1.43	0.00
ENSG00000069248	NUP133	0.72	-0.47	0.00
ENSG00000081913	PHLPP1	0.58	-0.80	0.00
ENSG00000138639	ARHGAP24	0.46	-1.13	0.00
ENSG00000136861	CDK5RAP2	0.68	-0.56	0.00
ENSG00000163633	C4orf36	0.16	-2.61	0.00
ENSG00000079432	CIC	0.74	-0.44	0.00
ENSG00000092847	AGO1	0.71	-0.49	0.00
ENSG00000143850	PLEKHA6	0.71	-0.49	0.00
ENSG00000143641	GALNT2	0.77	-0.37	0.00
ENSG00000112539	C6orf118	0.00	-11.35	0.00
ENSG00000071655	MBD3	0.71	-0.50	0.00
ENSG00000138606	SHF	0.28	-1.86	0.00
ENSG00000167815	PRDX2	0.58	-0.79	0.00
ENSG00000155974	GRIP1	0.55	-0.86	0.00
ENSG00000185033	SEMA4B	0.64	-0.64	0.00
ENSG00000159140	SON	0.76	-0.39	0.00
ENSG00000213551	DNAJC9	0.70	-0.52	0.00
ENSG00000122952	ZWINT	0.70	-0.51	0.00
ENSG00000141401	IMPA2	0.60	-0.73	0.00
ENSG00000160972	PPP1R16A	0.54	-0.90	0.00
ENSG00000198889	DCAF12L1	0.55	-0.87	0.00
ENSG00000176974	SHMT1	0.65	-0.62	0.00
ENSG00000277209	RPPH1	0.02	-5.44	0.00
ENSG00000145555	MYO10	0.58	-0.78	0.00
ENSG00000138030	KHK	0.34	-1.56	0.00
ENSG00000184602	SNN	0.66	-0.60	0.00
ENSG00000101639	CEP192	0.65	-0.62	0.00

ENSG00000163918	RFC4	0.60	-0.74	0.00
ENSG00000131351	HAUS8	0.65	-0.62	0.00
ENSG00000170921	TANC2	0.76	-0.40	0.00
ENSG00000136824	SMC2	0.56	-0.83	0.00
ENSG00000167978	SRRM2	0.71	-0.50	0.00
ENSG00000100014	SPECC1L	0.60	-0.73	0.00
ENSG00000166147	FBN1	0.74	-0.43	0.00
ENSG00000145425	RPS3A	0.60	-0.75	0.00
ENSG00000100473	COCH	0.54	-0.89	0.00
ENSG00000170653	ATF7	0.71	-0.49	0.00
ENSG00000133597	ADCK2	0.58	-0.80	0.00
ENSG00000168411	RFWD3	0.70	-0.52	0.00
ENSG00000173218	VANGL1	0.72	-0.47	0.00
ENSG00000183087	GAS6	0.70	-0.52	0.00
ENSG00000167799	NUDT8	0.25	-2.00	0.00
ENSG00000146373	RNF217	0.53	-0.91	0.00
ENSG00000198909	MAP3K3	0.73	-0.45	0.00
ENSG00000112983	BRD8	0.65	-0.62	0.00
ENSG00000186767	SPIN4	0.55	-0.87	0.00
ENSG00000162687	KCNT2	0.39	-1.34	0.00
ENSG00000178999	AURKB	0.66	-0.59	0.00
ENSG00000171467	ZNF318	0.69	-0.53	0.00
ENSG00000204852	TCTN1	0.60	-0.75	0.00
ENSG00000204217	BMPR2	0.69	-0.54	0.00
ENSG00000005483	KMT2E	0.67	-0.58	0.00
ENSG00000155313	USP25	0.58	-0.78	0.00
ENSG00000071539	TRIP13	0.73	-0.46	0.00
ENSG00000163872	YEATS2	0.72	-0.47	0.00
ENSG00000128242	GAL3ST1	0.19	-2.41	0.00
ENSG00000257167	TMPO-AS1	0.41	-1.29	0.00
ENSG00000228594	FNDC10	0.21	-2.23	0.00
ENSG00000138346	DNA2	0.55	-0.86	0.00
ENSG00000132670	PTPRA	0.73	-0.46	0.00
ENSG00000174106	LEMD3	0.64	-0.64	0.00
ENSG00000016082	ISL1	0.43	-1.23	0.00
ENSG00000081052	COL4A4	0.24	-2.03	0.00
ENSG00000197119	SLC25A29	0.67	-0.58	0.00
ENSG00000052126	PLEKHA5	0.60	-0.74	0.00
ENSG00000176678	FOXL1	0.56	-0.84	0.00
ENSG00000149311	ATM	0.62	-0.68	0.00
ENSG00000197296	FITM2	0.57	-0.81	0.00
ENSG00000175216	CKAP5	0.73	-0.45	0.00
ENSG00000154760	SLFN13	0.57	-0.80	0.00
ENSG00000198646	NCOA6	0.74	-0.44	0.00
ENSG00000075240	GRAMD4	0.57	-0.81	0.00
ENSG00000142327	RNPEPL1	0.48	-1.05	0.00
ENSG00000198056	PRIM1	0.59	-0.77	0.00
ENSG00000105875	WDR91	0.61	-0.72	0.00
ENSG00000150347	ARID5B	0.59	-0.76	0.00
ENSG00000111752	PHC1	0.67	-0.58	0.00
ENSG00000170881	RNF139	0.68	-0.55	0.00
ENSG00000109685	NSD2	0.55	-0.86	0.00
ENSG00000276386	CNTNAP3P2	0.17	-2.57	0.00
ENSG00000183722	LHFPL6	0.58	-0.78	0.00
ENSG00000101224	CDC25B	0.53	-0.92	0.00
ENSG00000145623	OSMR	0.77	-0.37	0.00
ENSG00000184304	PRKD1	0.56	-0.83	0.00
ENSG00000104332	SFRP1	0.00	-10.21	0.00
ENSG00000111912	NCOA7	0.55	-0.87	0.00
ENSG00000234184	LINC01781	0.16	-2.67	0.00
ENSG00000243709	LEFTY1	0.00	-11.51	0.00

ENSG00000046653	GPM6B	0.32	-1.65	0.00
ENSG00000177119	ANO6	0.71	-0.49	0.00
ENSG00000137700	SLC37A4	0.55	-0.87	0.00
ENSG00000160932	LY6E	0.64	-0.64	0.00
ENSG00000132478	UNK	0.73	-0.45	0.00
ENSG00000162976	SLC66A3	0.55	-0.86	0.00
ENSG00000126970	ZC4H2	0.59	-0.77	0.00
ENSG00000185275	CD24P4	0.06	-4.05	0.00
ENSG00000175455	CCDC14	0.49	-1.03	0.00
ENSG00000130638	ATXN10	0.75	-0.42	0.00
ENSG00000012504	NR1H4	0.33	-1.58	0.00
ENSG00000133030	MPRIP	0.73	-0.45	0.00
ENSG00000101236	RNF24	0.58	-0.79	0.00
ENSG00000138092	CENPO	0.52	-0.93	0.00
ENSG00000177469	CAVIN1	0.77	-0.38	0.00
ENSG00000093009	CDC45	0.64	-0.65	0.00
ENSG00000196950	SLC39A10	0.64	-0.64	0.00
ENSG00000171824	EXOSC10	0.75	-0.42	0.00
ENSG00000101057	MYBL2	0.75	-0.41	0.00
ENSG00000198700	IPO9	0.78	-0.36	0.00
ENSG00000100393	EP300	0.72	-0.48	0.00
ENSG00000078018	MAP2	0.42	-1.25	0.00
ENSG00000168916	ZNF608	0.71	-0.49	0.00
ENSG00000169057	MECP2	0.68	-0.55	0.00
ENSG00000108557	RAI1	0.74	-0.43	0.00
ENSG00000065413	ANKRD44	0.32	-1.66	0.00
ENSG00000099381	SETD1A	0.69	-0.54	0.00
ENSG00000182263	FIGN	0.67	-0.58	0.00
ENSG00000169564	PCBP1	0.77	-0.37	0.00
ENSG00000124160	NCOA5	0.71	-0.49	0.00
ENSG00000283809	AC007326	0.00	-11.56	0.00
ENSG00000198920	KIAA0753	0.65	-0.63	0.00
ENSG00000161800	RACGAP1	0.63	-0.66	0.00
ENSG00000143578	CREB3L4	0.55	-0.87	0.00
ENSG00000114698	PLSCR4	0.34	-1.55	0.00
ENSG00000174371	EXO1	0.64	-0.64	0.00
ENSG00000135108	FBXO21	0.62	-0.69	0.00
ENSG00000144730	IL17RD	0.28	-1.84	0.00
ENSG00000122642	FKBP9	0.53	-0.93	0.00
ENSG00000121211	MND1	0.53	-0.91	0.00
ENSG00000149269	PAK1	0.73	-0.45	0.00
ENSG00000170871	KIAA0232	0.70	-0.51	0.00
ENSG00000124098	FAM210B	0.63	-0.67	0.00
ENSG00000134775	FHOD3	0.74	-0.43	0.00
ENSG00000168672	LRATD2	0.62	-0.70	0.00
ENSG00000160948	VPS28	0.69	-0.53	0.00
ENSG00000173473	SMARCC1	0.74	-0.43	0.00
ENSG00000185000	DGAT1	0.57	-0.80	0.00
ENSG00000168014	C2CD3	0.69	-0.54	0.00
ENSG00000003056	M6PR	0.70	-0.52	0.00
ENSG00000135697	BCO1	0.33	-1.58	0.00
ENSG00000237296	SMG1P1	0.62	-0.70	0.00
ENSG00000167325	RRM1	0.76	-0.41	0.00
ENSG00000054965	FAM168A	0.75	-0.41	0.00
ENSG00000163435	ELF3	0.76	-0.39	0.00
ENSG00000143476	DTL	0.66	-0.59	0.00
ENSG00000172197	MBOAT1	0.42	-1.25	0.00
ENSG00000169375	SIN3A	0.74	-0.44	0.00
ENSG00000082512	TRAF5	0.66	-0.61	0.00
ENSG00000165650	PDZD8	0.71	-0.49	0.00
ENSG00000125798	FOXA2	0.18	-2.49	0.00

ENSG00000206127	GOLGA8O	0.12	-3.09	0.00
ENSG00000135845	PIGC	0.73	-0.45	0.00
ENSG00000162402	USP24	0.73	-0.46	0.00
ENSG00000092969	TGFB2	0.57	-0.81	0.00
ENSG00000104960	PTOV1	0.71	-0.48	0.00
ENSG00000197321	SVIL	0.77	-0.39	0.00
ENSG00000100726	TELO2	0.70	-0.51	0.00
ENSG00000205835	GMNC	0.08	-3.60	0.00
ENSG00000104067	TJP1	0.69	-0.53	0.00
ENSG00000038295	TLL1	0.04	-4.55	0.00
ENSG00000158163	DZIP1L	0.62	-0.69	0.00
ENSG00000108370	RGS9	0.53	-0.91	0.00
ENSG00000105397	TYK2	0.68	-0.55	0.00
ENSG00000117650	NEK2	0.61	-0.72	0.00
ENSG00000115687	PASK	0.59	-0.77	0.00
ENSG00000163125	RPRD2	0.76	-0.40	0.00
ENSG00000204991	SPIRE2	0.55	-0.87	0.00
ENSG00000152990	ADGRA3	0.74	-0.43	0.00
ENSG00000168496	FEN1	0.73	-0.44	0.00
ENSG00000159692	CTBP1	0.76	-0.40	0.00
ENSG00000130313	PGLS	0.71	-0.50	0.00
ENSG00000111011	RSRC2	0.62	-0.70	0.00
ENSG00000131508	UBE2D2	0.77	-0.38	0.00
ENSG00000138772	ANXA3	0.26	-1.96	0.00
ENSG00000144711	IQSEC1	0.67	-0.58	0.00
ENSG00000126001	CEP250	0.73	-0.45	0.00
ENSG00000168936	TMEM129	0.72	-0.48	0.00
ENSG00000108312	UBTF	0.76	-0.39	0.00
ENSG00000188707	ZBED6CL	0.47	-1.08	0.00
ENSG00000198121	LPAR1	0.55	-0.87	0.00
ENSG00000147799	ARHGAP39	0.61	-0.70	0.00
ENSG00000197694	SPTAN1	0.77	-0.37	0.00
ENSG00000174099	MSRB3	0.70	-0.52	0.00
ENSG00000008382	MPND	0.54	-0.88	0.00
ENSG00000167889	MGAT5B	0.40	-1.31	0.00
ENSG00000015171	ZMYND11	0.48	-1.04	0.00
ENSG00000157870	PRXL2B	0.27	-1.89	0.00
ENSG00000178695	KCTD12	0.67	-0.57	0.00
ENSG00000204406	MBD5	0.56	-0.84	0.00
ENSG00000125885	MCM8	0.65	-0.62	0.00
ENSG00000060237	WNK1	0.71	-0.50	0.00
ENSG00000225614	ZNF469	0.24	-2.06	0.00
ENSG00000177570	SAMD12	0.55	-0.85	0.00
ENSG00000111665	CDCA3	0.65	-0.62	0.00
ENSG00000150403	TMCO3	0.75	-0.41	0.00
ENSG00000096060	FKBP5	0.70	-0.51	0.00
ENSG00000129353	SLC44A2	0.67	-0.58	0.00
ENSG00000054118	THRAP3	0.78	-0.35	0.00
ENSG00000079931	MOXD1	0.71	-0.49	0.00
ENSG00000186187	ZNRF1	0.52	-0.95	0.00
ENSG00000172379	ARNT2	0.20	-2.29	0.00
ENSG00000214425	LRRC37A4P	0.55	-0.85	0.00
ENSG00000132207	SLX1A	0.61	-0.72	0.00
ENSG00000148700	ADD3	0.68	-0.57	0.00
ENSG00000268043	NBPF12	0.67	-0.57	0.00
ENSG00000137834	SMAD6	0.64	-0.64	0.00
ENSG00000196914	ARHGEF12	0.66	-0.60	0.00
ENSG00000138031	ADCY3	0.62	-0.70	0.00
ENSG00000161999	JMJD8	0.72	-0.48	0.00
ENSG00000148773	MKI67	0.54	-0.90	0.00
ENSG00000072864	NDE1	0.66	-0.61	0.00

ENSG00000074800	ENO1	0.76	-0.40	0.00
ENSG00000122779	TRIM24	0.72	-0.48	0.00
ENSG00000090565	RAB11FIP3	0.74	-0.44	0.00
ENSG00000196547	MAN2A2	0.59	-0.77	0.00
ENSG00000271092	TLCD4-RWDD3	0.00	-14.17	0.00
ENSG00000065060	UHRF1BP1	0.73	-0.45	0.00
ENSG00000126217	MCF2L	0.12	-3.08	0.00
ENSG00000146143	PRIM2	0.68	-0.55	0.00
ENSG00000107554	DNMBP	0.76	-0.40	0.00
ENSG00000080839	RBL1	0.64	-0.64	0.00
ENSG00000004799	PDK4	0.36	-1.46	0.00
ENSG00000143442	POGZ	0.69	-0.53	0.00
ENSG00000087903	RFX2	0.49	-1.02	0.00
ENSG00000137135	ARHGEF39	0.55	-0.87	0.00
ENSG00000170915	PAQR8	0.57	-0.81	0.00
ENSG00000198899	MT-ATP6	0.79	-0.33	0.00
ENSG00000257365	FNTB	0.62	-0.68	0.00
ENSG00000152953	STK32B	0.60	-0.74	0.00
ENSG00000100311	PDGFB	0.43	-1.20	0.00
ENSG00000169683	LRRC45	0.70	-0.52	0.00
ENSG00000012660	ELOVL5	0.77	-0.38	0.00
ENSG00000138180	CEP55	0.70	-0.52	0.00
ENSG00000145416	MARCHF1	0.39	-1.35	0.00
ENSG00000118193	KIF14	0.59	-0.75	0.00
ENSG00000112655	PTK7	0.68	-0.56	0.00
ENSG00000164142	FAM160A1	0.49	-1.03	0.00
ENSG00000121653	MAPK8IP1	0.51	-0.97	0.00
ENSG00000279041	AC102945	0.46	-1.13	0.00
ENSG00000197858	GPAA1	0.75	-0.41	0.00
ENSG00000116539	ASH1L	0.73	-0.46	0.00
ENSG00000183840	GPR39	0.64	-0.63	0.00
ENSG00000175048	ZDHHC14	0.55	-0.86	0.00
ENSG00000079805	DNM2	0.75	-0.42	0.00
ENSG00000221869	CEBPD	0.75	-0.41	0.00
ENSG00000124207	CSE1L	0.75	-0.41	0.00
ENSG00000072195	SPEG	0.67	-0.58	0.00
ENSG00000109861	CTSC	0.75	-0.42	0.00
ENSG00000115163	CENPA	0.37	-1.43	0.00
ENSG00000237914	SIRPG-AS1	0.25	-2.01	0.00
ENSG00000094631	HDAC6	0.73	-0.46	0.00
ENSG00000177054	ZDHHC13	0.67	-0.58	0.00
ENSG00000166503	HDGFL3	0.68	-0.56	0.00
ENSG00000087111	PIGS	0.77	-0.39	0.00
ENSG00000147130	ZMYM3	0.75	-0.42	0.00
ENSG00000166971	AKTIP	0.59	-0.76	0.00
ENSG00000119772	DNMT3A	0.68	-0.55	0.00
ENSG00000213648	SULT1A4	0.37	-1.44	0.00
ENSG00000177990	DPY19L2	0.44	-1.19	0.00
ENSG00000171320	ESCO2	0.54	-0.88	0.00
ENSG00000136044	APPL2	0.64	-0.65	0.00
ENSG00000197746	PSAP	0.80	-0.32	0.00
ENSG00000204525	HLA-C	0.59	-0.76	0.00
ENSG00000118217	ATF6	0.76	-0.40	0.00
ENSG00000213672	NCKIPSD	0.69	-0.53	0.00
ENSG00000025770	NCAPH2	0.70	-0.51	0.00
ENSG00000070950	RAD18	0.57	-0.80	0.00
ENSG00000112343	TRIM38	0.76	-0.40	0.00
ENSG00000114861	FOXP1	0.61	-0.71	0.00
ENSG00000134247	PTGFRN	0.79	-0.34	0.00
ENSG00000180730	SHISA2	0.78	-0.35	0.00
ENSG00000171105	INSR	0.17	-2.60	0.00

ENSG00000184220	CMSS1	0.74	-0.44	0.00
ENSG00000170500	LONRF2	0.68	-0.56	0.00
ENSG00000104472	CHRAC1	0.65	-0.62	0.00
ENSG00000165392	WRN	0.55	-0.86	0.00
ENSG00000119900	OGFRL1	0.74	-0.43	0.00
ENSG00000135749	PCNX2	0.67	-0.58	0.00
ENSG00000198932	GPRASP1	0.12	-3.08	0.00
ENSG00000106351	AGFG2	0.60	-0.75	0.00
ENSG00000266074	BAHCC1	0.61	-0.72	0.00
ENSG00000102981	PARD6A	0.20	-2.32	0.00
ENSG00000160298	C21orf58	0.43	-1.20	0.00
ENSG00000228262	LINC01320	0.10	-3.27	0.00
ENSG00000070669	ASNS	0.72	-0.48	0.00
ENSG00000010292	NCAPD2	0.74	-0.43	0.00
ENSG00000235092	ID2-AS1	0.41	-1.30	0.00
ENSG00000177706	FAM20C	0.29	-1.79	0.00
ENSG00000123473	STIL	0.59	-0.75	0.00
ENSG00000141258	SGSM2	0.74	-0.44	0.00
ENSG00000111674	ENO2	0.71	-0.50	0.00
ENSG00000181938	GINS3	0.62	-0.70	0.00
ENSG00000166484	MAPK7	0.59	-0.76	0.00
ENSG00000100242	SUN2	0.56	-0.84	0.00
ENSG00000163075	CFAP221	0.27	-1.87	0.00
ENSG00000180357	ZNF609	0.77	-0.39	0.00
ENSG00000138756	BMP2K	0.71	-0.50	0.00
ENSG00000174373	RALGAPA1	0.51	-0.98	0.00
ENSG00000143819	EPHX1	0.76	-0.40	0.00
ENSG00000173273	TNKS	0.75	-0.41	0.00
ENSG00000168792	ABHD15	0.69	-0.54	0.00
ENSG00000080815	PSEN1	0.74	-0.43	0.00
ENSG00000108100	CCNY	0.70	-0.52	0.00
ENSG00000015676	NUDCD3	0.75	-0.41	0.00
ENSG00000124920	MYRF	0.40	-1.34	0.00
ENSG00000001561	ENPP4	0.58	-0.80	0.00
ENSG00000183458	AC138932	0.62	-0.68	0.00
ENSG00000167460	TPM4	0.65	-0.63	0.00
ENSG00000153187	HNRNPU	0.77	-0.37	0.00
ENSG00000163913	IFT122	0.67	-0.58	0.00
ENSG00000186638	KIF24	0.56	-0.83	0.00
ENSG00000089091	DZANK1	0.36	-1.47	0.00
ENSG00000204536	CCHCR1	0.70	-0.52	0.00
ENSG00000185361	TNFAIP8L1	0.69	-0.54	0.00
ENSG00000166881	NEMP1	0.70	-0.51	0.00
ENSG00000171962	DRC3	0.35	-1.51	0.00
ENSG00000133466	C1QTNF6	0.63	-0.66	0.00
ENSG00000161618	ALDH16A1	0.69	-0.54	0.00
ENSG00000235333	PVRIG2P	0.44	-1.19	0.00
ENSG00000139718	SETD1B	0.73	-0.46	0.00
ENSG00000142089	IFITM3	0.75	-0.42	0.00
ENSG00000143158	MPC2	0.56	-0.84	0.00
ENSG00000132646	PCNA	0.72	-0.48	0.00
ENSG00000184178	SCFD2	0.69	-0.54	0.00
ENSG00000156052	GNAQ	0.73	-0.45	0.00
ENSG00000136485	DCAF7	0.79	-0.34	0.00
ENSG00000091844	RGS17	0.56	-0.83	0.00
ENSG00000168385	SEPTIN2	0.66	-0.59	0.00
ENSG00000156381	ANKRD9	0.54	-0.89	0.00
ENSG00000170214	ADRA1B	0.42	-1.24	0.00
ENSG00000231890	DARS-AS1	0.21	-2.24	0.00
ENSG00000187653	TMSB4XP8	0.64	-0.65	0.00
ENSG00000130635	COL5A1	0.74	-0.44	0.00

ENSG00000135776	ABCB10	0.71	-0.50	0.00
ENSG00000196712	NF1	0.76	-0.40	0.00
ENSG00000171150	SOCS5	0.68	-0.56	0.00
ENSG00000089280	FUS	0.79	-0.34	0.00
ENSG00000225733	FGD5-AS1	0.76	-0.39	0.00
ENSG00000197586	ENTPD6	0.77	-0.38	0.00
ENSG00000167523	SPATA33	0.55	-0.86	0.00
ENSG00000101474	APMAP	0.77	-0.38	0.00
ENSG00000224712	NPIPA3	0.00	-11.71	0.00
ENSG00000115170	ACVR1	0.72	-0.48	0.00
ENSG00000189079	ARID2	0.62	-0.70	0.00
ENSG00000022567	SLC45A4	0.67	-0.58	0.00
ENSG00000057294	PKP2	0.52	-0.93	0.00
ENSG00000246695	RASSF8-AS1	0.54	-0.88	0.00
ENSG00000110321	EIF4G2	0.77	-0.39	0.00
ENSG00000184584	STING1	0.67	-0.58	0.00
ENSG00000279019	AC009090	0.26	-1.96	0.00
ENSG00000177602	HASPIN	0.63	-0.67	0.00
ENSG00000160957	RECQL4	0.67	-0.58	0.00
ENSG00000196663	TECPR2	0.72	-0.48	0.00
ENSG00000105516	DBP	0.47	-1.09	0.00
ENSG00000198363	ASPH	0.77	-0.37	0.00
ENSG00000171889	MIR31HG	0.35	-1.52	0.00
ENSG00000197603	CPLANE1	0.64	-0.65	0.00
ENSG00000206560	ANKRD28	0.63	-0.67	0.00
ENSG00000185324	CDK10	0.64	-0.63	0.00
ENSG00000186260	MRTFB	0.70	-0.51	0.00
ENSG00000115760	BIRC6	0.73	-0.46	0.00
ENSG00000090581	GNPTG	0.73	-0.46	0.00
ENSG00000105085	MED26	0.43	-1.23	0.00
ENSG00000068796	KIF2A	0.73	-0.46	0.00
ENSG00000196821	ILRUN	0.79	-0.35	0.00
ENSG00000260604	AL590004	0.55	-0.86	0.00
ENSG00000152495	CAMK4	0.58	-0.80	0.00
ENSG00000159842	ABR	0.77	-0.38	0.00
ENSG00000239305	RNF103	0.68	-0.57	0.00
ENSG00000176087	SLC35A4	0.77	-0.37	0.00
ENSG00000253187	HOXA10-AS	0.31	-1.67	0.00
ENSG00000112893	MAN2A1	0.69	-0.53	0.00
ENSG00000154920	EME1	0.63	-0.66	0.00
ENSG00000136813	ECPAS	0.77	-0.38	0.00
ENSG00000164010	ERMAP	0.65	-0.61	0.00
ENSG00000150551	LYPD1	0.16	-2.63	0.00
ENSG00000078900	TP73	0.03	-4.90	0.00
ENSG00000144481	TRPM8	0.67	-0.57	0.00
ENSG00000057608	GDI2	0.80	-0.32	0.00
ENSG00000166340	TPP1	0.61	-0.70	0.00
ENSG00000100258	LMF2	0.61	-0.72	0.00
ENSG00000134291	TMEM106C	0.76	-0.39	0.00
ENSG00000165312	OTUD1	0.48	-1.05	0.00
ENSG00000163348	PYGO2	0.76	-0.39	0.00
ENSG00000116670	MAD2L2	0.71	-0.50	0.00
ENSG00000160325	CACFD1	0.55	-0.87	0.00
ENSG00000157445	CACNA2D3	0.43	-1.22	0.00
ENSG00000075151	EIF4G3	0.76	-0.39	0.00
ENSG00000113273	ARSB	0.71	-0.50	0.00
ENSG00000172817	CYP7B1	0.56	-0.83	0.00
ENSG00000153707	PTPRD	0.68	-0.55	0.00
ENSG00000145730	PAM	0.76	-0.39	0.00
ENSG00000065883	CDK13	0.75	-0.41	0.00
ENSG00000146463	ZMYM4	0.76	-0.40	0.00

ENSG00000183579	ZNRF3	0.59	-0.75	0.00
ENSG00000093167	LRRFIP2	0.67	-0.57	0.00
ENSG00000143614	GATAD2B	0.77	-0.38	0.00
ENSG00000120071	KANSL1	0.71	-0.49	0.00
ENSG00000005339	CREBBP	0.69	-0.53	0.00
ENSG00000034053	APBA2	0.70	-0.52	0.00
ENSG00000172954	LCLAT1	0.61	-0.71	0.00
ENSG00000023697	DERA	0.72	-0.48	0.00
ENSG00000197111	PCBP2	0.70	-0.52	0.00
ENSG00000129667	RHBDF2	0.77	-0.38	0.00
ENSG00000214826	DDX12P	0.54	-0.90	0.00
ENSG00000099331	MYO9B	0.78	-0.35	0.00
ENSG00000046889	PREX2	0.66	-0.59	0.00
ENSG00000118482	PHF3	0.68	-0.55	0.00
ENSG00000158417	EIF5B	0.74	-0.44	0.00
ENSG00000136205	TNS3	0.79	-0.34	0.00
ENSG00000129534	MIS18BP1	0.59	-0.76	0.00
ENSG00000160447	PKN3	0.58	-0.80	0.00
ENSG00000196517	SLC6A9	0.32	-1.65	0.00
ENSG00000111788	AC009533	0.52	-0.93	0.00
ENSG00000247809	NR2F2-AS1	0.38	-1.38	0.00
ENSG00000257103	LSM14A	0.70	-0.51	0.00
ENSG00000179750	APOBEC3B	0.69	-0.53	0.00
ENSG00000126804	ZBTB1	0.58	-0.79	0.00
ENSG00000115290	GRB14	0.64	-0.64	0.00
ENSG00000171723	GPHN	0.61	-0.71	0.00
ENSG00000141497	ZMYND15	0.00	-10.06	0.00
ENSG00000136379	ABHD17C	0.54	-0.88	0.00
ENSG00000091129	NRCAM	0.37	-1.44	0.00
ENSG00000159259	CHAF1B	0.69	-0.54	0.00
ENSG00000106070	GRB10	0.74	-0.43	0.00
ENSG00000135744	AGT	0.17	-2.59	0.00
ENSG00000134318	ROCK2	0.71	-0.49	0.00
ENSG00000089902	RCOR1	0.70	-0.51	0.00
ENSG00000198938	MT-CO3	0.82	-0.29	0.00
ENSG00000121621	KIF18A	0.52	-0.95	0.00
ENSG00000155016	CYP2U1	0.57	-0.80	0.00
ENSG00000112742	TTK	0.50	-0.99	0.00
ENSG00000115904	SOS1	0.73	-0.45	0.00
ENSG00000091583	APOH	0.00	-11.72	0.00
ENSG00000146038	DCDC2	0.38	-1.39	0.00
ENSG00000140451	PIF1	0.51	-0.97	0.00
ENSG00000172292	CERS6	0.73	-0.45	0.00
ENSG00000012232	EXTL3	0.76	-0.40	0.00
ENSG00000181666	ZNF875	0.61	-0.72	0.00
ENSG00000167900	TK1	0.80	-0.32	0.00
ENSG00000169242	EFNA1	0.29	-1.76	0.00
ENSG00000127995	CASD1	0.60	-0.74	0.00
ENSG00000139921	TMX1	0.72	-0.47	0.00
ENSG00000235531	MSC-AS1	0.63	-0.66	0.00
ENSG00000139354	GAS2L3	0.57	-0.82	0.00
ENSG00000109674	NEIL3	0.64	-0.65	0.00
ENSG00000123066	MED13L	0.67	-0.58	0.00
ENSG00000137404	NRM	0.57	-0.80	0.00
ENSG00000118058	KMT2A	0.74	-0.43	0.00
ENSG00000129636	ITFG1	0.75	-0.42	0.00
ENSG00000113971	NPHP3	0.67	-0.58	0.00
ENSG00000198049	AVPR1B	0.14	-2.85	0.00
ENSG00000198887	SMC5	0.68	-0.56	0.00
ENSG00000105011	ASF1B	0.67	-0.57	0.00
ENSG00000166851	PLK1	0.78	-0.36	0.00

ENSG00000231925	TAPBP	0.75	-0.42	0.00
ENSG00000263535	AK4P1	0.39	-1.37	0.00
ENSG00000168488	ATXN2L	0.79	-0.34	0.00
ENSG00000229656	ITGB1-DT	0.72	-0.47	0.00
ENSG00000179820	MYADM	0.66	-0.59	0.00
ENSG00000144036	EXOC6B	0.68	-0.55	0.00
ENSG00000101126	ADNP	0.30	-1.73	0.00
ENSG00000167074	TEF	0.70	-0.52	0.00
ENSG00000197557	TTC30A	0.60	-0.73	0.00
ENSG00000112541	PDE10A	0.64	-0.65	0.00
ENSG00000140575	IQGAP1	0.79	-0.33	0.00
ENSG00000051341	POLQ	0.62	-0.70	0.00
ENSG00000160226	CFAP410	0.63	-0.67	0.00
ENSG00000245694	CRNDE	0.54	-0.90	0.00
ENSG00000164111	ANXA5	0.80	-0.32	0.00
ENSG00000115504	EHBP1	0.72	-0.48	0.00
ENSG00000169814	BTD	0.58	-0.78	0.00
ENSG00000126705	AHDC1	0.70	-0.51	0.00
ENSG00000278621	AC037198	0.06	-4.04	0.00
ENSG00000107779	BMPR1A	0.71	-0.49	0.00
ENSG00000137269	LRRC1	0.57	-0.80	0.00
ENSG00000176720	BOK	0.60	-0.73	0.00
ENSG00000184154	LRTOMT	0.58	-0.78	0.00
ENSG00000151690	MFSD6	0.58	-0.78	0.00
ENSG00000004777	ARHGAP33	0.45	-1.14	0.00
ENSG00000188985	DHFRP1	0.69	-0.54	0.00
ENSG00000111801	BTN3A3	0.60	-0.73	0.00
ENSG00000035687	ADSS2	0.77	-0.38	0.00
ENSG00000111859	NEDD9	0.14	-2.83	0.00
ENSG00000152117	SMPD4BP	0.72	-0.48	0.00
ENSG00000096093	EFHC1	0.49	-1.04	0.00
ENSG00000100490	CDKL1	0.42	-1.25	0.00
ENSG00000136816	TOR1B	0.67	-0.57	0.00
ENSG00000186153	WWOX	0.55	-0.87	0.00
ENSG00000137177	KIF13A	0.75	-0.42	0.00
ENSG00000196814	MVB12B	0.59	-0.76	0.00
ENSG00000121207	LRAT	0.25	-1.99	0.00
ENSG00000141556	TBCD	0.79	-0.35	0.00
ENSG00000049541	RFC2	0.71	-0.49	0.00
ENSG00000152076	CCDC74B	0.55	-0.87	0.00
ENSG00000110422	HIPK3	0.69	-0.54	0.00
ENSG00000256514	AP003419	0.00	-12.24	0.00
ENSG00000164366	CCDC127	0.75	-0.41	0.00
ENSG00000005810	MYCBP2	0.72	-0.48	0.00
ENSG00000136237	RAPGEF5	0.33	-1.60	0.00
ENSG00000249042	AC008771	0.44	-1.20	0.00
ENSG00000109381	ELF2	0.69	-0.53	0.00
ENSG00000213079	SCAF8	0.72	-0.47	0.00
ENSG00000117523	PRRC2C	0.81	-0.30	0.00
ENSG00000085840	ORC1	0.67	-0.58	0.00
ENSG00000102974	CTCF	0.74	-0.44	0.00
ENSG00000138764	CCNG2	0.72	-0.47	0.00
ENSG00000164880	INTS1	0.78	-0.35	0.00
ENSG00000145241	CENPC	0.56	-0.83	0.00
ENSG00000196924	FLNA	0.79	-0.34	0.00
ENSG00000268120	AC010336	0.00	-12.33	0.00
ENSG00000113594	LIFR	0.65	-0.63	0.00
ENSG00000166780	BMERB1	0.54	-0.88	0.00
ENSG00000204120	GIGYF2	0.78	-0.36	0.00
ENSG00000166734	GOLM2	0.70	-0.51	0.00
ENSG00000248527	MTATP6P1	0.79	-0.34	0.00

ENSG00000013374	NUB1	0.75	-0.41	0.00
ENSG00000196739	COL27A1	0.71	-0.50	0.00
ENSG00000093000	NUP50	0.75	-0.42	0.00
ENSG00000198586	TLK1	0.72	-0.48	0.00
ENSG00000197816	CCDC180	0.26	-1.95	0.00
ENSG00000128487	SPECC1	0.70	-0.51	0.00
ENSG00000047578	KIAA0556	0.75	-0.42	0.00
ENSG00000169684	CHRNA5	0.56	-0.83	0.00
ENSG00000171681	ATF7IP	0.74	-0.43	0.00
ENSG00000082196	C1QTNF3	0.08	-3.71	0.00
ENSG00000158402	CDC25C	0.60	-0.73	0.00
ENSG00000080200	CRYBG3	0.65	-0.62	0.00
ENSG00000012963	UBR7	0.68	-0.56	0.00
ENSG00000108465	CDK5RAP3	0.65	-0.61	0.00
ENSG00000171914	TLN2	0.69	-0.53	0.00
ENSG00000184634	MED12	0.76	-0.40	0.00
ENSG00000260279	AC137932	0.40	-1.33	0.00
ENSG00000219481	NBPF1	0.76	-0.39	0.00
ENSG00000102595	UGGT2	0.62	-0.69	0.00
ENSG00000166228	PCBD1	0.79	-0.34	0.00
ENSG00000152217	SETBP1	0.52	-0.95	0.00
ENSG00000138468	SENP7	0.52	-0.95	0.00
ENSG00000107290	SETX	0.73	-0.45	0.00
ENSG00000187097	ENTPD5	0.59	-0.75	0.00
ENSG00000187951	AC091057	0.57	-0.81	0.00
ENSG00000112739	PRPF4B	0.73	-0.45	0.00
ENSG00000143153	ATP1B1	0.79	-0.33	0.00
ENSG00000135220	UGT2A3	0.04	-4.75	0.00
ENSG00000143195	ILDR2	0.33	-1.59	0.00
ENSG00000131504	DIAPH1	0.79	-0.34	0.00
ENSG00000114544	SLC41A3	0.69	-0.53	0.00
ENSG00000214367	HAUS3	0.64	-0.64	0.00
ENSG00000125637	PSD4	0.53	-0.91	0.00
ENSG00000133874	RNF122	0.41	-1.30	0.00
ENSG00000224411	HSP90AA2P	0.65	-0.62	0.00
ENSG00000167535	CACNB3	0.41	-1.28	0.00
ENSG00000008869	HEATR5B	0.70	-0.52	0.00
ENSG00000160293	VAV2	0.78	-0.36	0.00
ENSG00000033867	SLC4A7	0.66	-0.59	0.00
ENSG00000136104	RNASEH2B	0.72	-0.48	0.00
ENSG00000147475	ERLIN2	0.78	-0.35	0.00
ENSG00000239264	TXNDC5	0.81	-0.30	0.00
ENSG00000105137	SYDE1	0.75	-0.41	0.00
ENSG00000211592	IGKC	0.00	-12.60	0.00
ENSG00000064999	ANKS1A	0.76	-0.40	0.00
ENSG00000282458	WASH5P	0.26	-1.94	0.00
ENSG00000287263	AC008875	0.59	-0.75	0.00
ENSG00000134480	CCNH	0.67	-0.59	0.00
ENSG00000102287	GABRE	0.67	-0.57	0.00
ENSG00000165282	PIGO	0.66	-0.60	0.00
ENSG00000124193	SRSF6	0.71	-0.49	0.00
ENSG00000160007	ARHGAP35	0.80	-0.32	0.00
ENSG00000105443	CYTH2	0.77	-0.38	0.00
ENSG00000136504	KAT7	0.74	-0.43	0.00
ENSG00000259291	ZNF710-AS1	0.48	-1.06	0.00
ENSG00000115392	FANCL	0.64	-0.65	0.00
ENSG00000123908	AGO2	0.79	-0.35	0.00
ENSG00000162688	AGL	0.62	-0.69	0.00
ENSG00000072310	SREBF1	0.71	-0.50	0.00
ENSG00000185920	PTCH1	0.65	-0.62	0.00
ENSG00000157657	ZNF618	0.45	-1.15	0.00

ENSG00000205189	ZBTB10	0.61	-0.71	0.00
ENSG00000047644	WWC3	0.72	-0.47	0.00
ENSG00000273270	AC090114	0.52	-0.95	0.00
ENSG00000120925	RNF170	0.65	-0.63	0.00
ENSG00000142731	PLK4	0.58	-0.79	0.00
ENSG00000169213	RAB3B	0.78	-0.36	0.00
ENSG00000215421	ZNF407	0.61	-0.72	0.00
ENSG00000173575	CHD2	0.78	-0.37	0.00
ENSG00000157500	APPL1	0.70	-0.52	0.00
ENSG00000197724	PHF2	0.72	-0.46	0.00
ENSG00000171634	BPTF	0.77	-0.37	0.00
ENSG00000198720	ANKRD13B	0.65	-0.62	0.00
ENSG00000173674	EIF1AX	0.80	-0.32	0.00
ENSG00000158987	RAPGEF6	0.66	-0.61	0.00
ENSG00000275202	AL161421	0.29	-1.77	0.00
ENSG00000112159	MDN1	0.77	-0.38	0.00
ENSG00000187098	MITF	0.46	-1.11	0.00
ENSG00000147144	CCDC120	0.31	-1.67	0.00
ENSG00000105983	LMBR1	0.73	-0.46	0.00
ENSG00000164576	SAP30L	0.73	-0.45	0.00
ENSG00000145777	TSLP	0.19	-2.38	0.00
ENSG00000151208	DLG5	0.76	-0.39	0.00
ENSG00000196155	PLEKHG4	0.77	-0.37	0.00
ENSG00000136141	LRCH1	0.70	-0.52	0.00
ENSG00000153822	KCNJ16	0.73	-0.46	0.00
ENSG00000073711	PPP2R3A	0.62	-0.69	0.00
ENSG00000005889	ZFX	0.68	-0.56	0.00
ENSG00000012048	BRCA1	0.68	-0.56	0.00
ENSG00000164062	APEH	0.79	-0.35	0.00
ENSG00000168734	PKIG	0.66	-0.60	0.00
ENSG00000137710	RDX	0.77	-0.39	0.00
ENSG00000126870	WDR60	0.71	-0.50	0.00
ENSG00000153914	SREK1	0.72	-0.48	0.00
ENSG00000114737	CISH	0.31	-1.71	0.00
ENSG00000167522	ANKRD11	0.80	-0.33	0.00
ENSG00000240038	AMY2B	0.39	-1.35	0.00
ENSG00000213983	AP1G2	0.72	-0.47	0.00
ENSG00000128604	IRF5	0.52	-0.94	0.00
ENSG00000143630	HCN3	0.60	-0.74	0.00
ENSG00000155097	ATP6V1C1	0.78	-0.36	0.00
ENSG00000087460	GNAS	0.72	-0.47	0.00
ENSG00000179981	TSHZ1	0.60	-0.73	0.00
ENSG00000230445	LRRC37A6P	0.27	-1.86	0.00
ENSG00000185565	LSAMP	0.34	-1.57	0.00
ENSG00000197363	ZNF517	0.50	-0.99	0.00
ENSG00000241684	ADAMTS9-AS2	0.17	-2.52	0.00
ENSG00000186350	RXRA	0.77	-0.37	0.00
ENSG00000174628	IQCK	0.51	-0.97	0.00
ENSG00000135473	PAN2	0.68	-0.55	0.00
ENSG00000136982	DSCC1	0.63	-0.67	0.00
ENSG00000140905	GCSH	0.75	-0.42	0.00
ENSG00000122687	MRM2	0.74	-0.43	0.00
ENSG00000158158	CNNM4	0.72	-0.47	0.00
ENSG00000198911	SREBF2	0.78	-0.36	0.00
ENSG00000120693	SMAD9	0.47	-1.10	0.00
ENSG00000118705	RPN2	0.82	-0.28	0.00
ENSG00000154429	CCSAP	0.68	-0.56	0.00
ENSG00000170390	DCLK2	0.37	-1.44	0.00
ENSG00000187535	IFT140	0.69	-0.54	0.00
ENSG00000123096	SSPN	0.54	-0.89	0.00
ENSG00000035499	DEPDC1B	0.68	-0.55	0.00

ENSG00000004838	ZMYND10	0.12	-3.02	0.00
ENSG00000072954	TMEM38A	0.38	-1.41	0.00
ENSG00000117791	MTARC2	0.62	-0.69	0.00
ENSG00000244486	SCARF2	0.59	-0.77	0.00
ENSG00000054690	PLEKHH1	0.43	-1.21	0.00
ENSG00000140682	TGFB1I1	0.66	-0.60	0.00
ENSG00000135451	TROAP	0.68	-0.55	0.00
ENSG00000051180	RAD51	0.61	-0.71	0.00
ENSG00000269113	TRABD2B	0.00	-8.63	0.00
ENSG00000251661	AC136475	0.00	-10.61	0.00
ENSG00000215912	TTC34	0.00	-8.41	0.00
ENSG00000114166	KAT2B	0.61	-0.71	0.00
ENSG00000231160	KLF3-AS1	0.26	-1.92	0.00
ENSG00000126016	AMOT	0.69	-0.53	0.00
ENSG00000162520	SYNC	0.45	-1.15	0.00
ENSG00000127423	AUNIP	0.50	-0.99	0.00
ENSG00000110025	SNX15	0.38	-1.39	0.00
ENSG00000198087	CD2AP	0.75	-0.42	0.00
ENSG00000154237	LRRK1	0.78	-0.35	0.00
ENSG00000167182	SP2	0.70	-0.52	0.00
ENSG00000006047	YBX2	0.16	-2.68	0.00
ENSG00000104983	CCDC61	0.62	-0.68	0.00
ENSG00000142156	COL6A1	0.82	-0.29	0.00
ENSG00000176444	CLK2	0.75	-0.42	0.00
ENSG00000088340	FER1L4	0.53	-0.92	0.00
ENSG00000164190	NIPBL	0.70	-0.52	0.00
ENSG00000125450	NUP85	0.74	-0.43	0.00
ENSG00000130402	ACTN4	0.78	-0.35	0.00
ENSG00000168078	PBK	0.70	-0.51	0.00
ENSG00000204623	ZNRD1ASP	0.49	-1.03	0.00
ENSG00000100722	ZC3H14	0.70	-0.52	0.00
ENSG00000104626	ERI1	0.70	-0.51	0.00
ENSG00000213639	PPP1CB	0.79	-0.34	0.00
ENSG00000115255	REEP6	0.51	-0.96	0.00
ENSG00000123643	SLC36A1	0.65	-0.61	0.00
ENSG00000141627	DYM	0.70	-0.51	0.00
ENSG00000267801	AC087289	0.48	-1.06	0.00
ENSG00000243156	MICAL3	0.69	-0.54	0.00
ENSG00000123219	CENPK	0.60	-0.73	0.00
ENSG00000172469	MANEA	0.53	-0.92	0.00
ENSG00000223802	CERS1	0.43	-1.22	0.00
ENSG00000137478	FCHSD2	0.69	-0.53	0.00
ENSG00000221988	PPT2	0.69	-0.54	0.00
ENSG00000172534	HCFC1	0.79	-0.34	0.00
ENSG00000184307	ZDHHC23	0.66	-0.59	0.00
ENSG00000169679	BUB1	0.76	-0.39	0.00
ENSG00000189339	SLC35E2B	0.78	-0.36	0.00
ENSG00000166803	PCLAF	0.77	-0.37	0.00
ENSG00000277701	AC159540	0.48	-1.07	0.00
ENSG00000272462	U91328	0.39	-1.35	0.00
ENSG00000119285	HEATR1	0.80	-0.32	0.00
ENSG00000185236	RAB11B	0.79	-0.34	0.00
ENSG00000116678	LEPR	0.62	-0.70	0.00
ENSG00000139973	SYT16	0.47	-1.09	0.00
ENSG00000110583	NAA40	0.74	-0.43	0.00
ENSG00000196159	FAT4	0.39	-1.36	0.00
ENSG00000151789	ZNF385D	0.51	-0.99	0.00
ENSG00000164904	ALDH7A1	0.79	-0.35	0.00
ENSG00000065613	SLK	0.70	-0.51	0.00
ENSG00000204442	FAM155A	0.53	-0.91	0.00
ENSG00000137166	FOXP4	0.79	-0.33	0.00

ENSG00000140598	EFL1	0.70	-0.52	0.00
ENSG00000062716	VMP1	0.80	-0.32	0.00
ENSG00000148082	SHC3	0.69	-0.53	0.00
ENSG00000073111	MCM2	0.76	-0.39	0.00
ENSG00000169919	GUSB	0.66	-0.59	0.00
ENSG00000116005	PCYOX1	0.77	-0.37	0.00
ENSG00000286112	AL441992	0.33	-1.58	0.00
ENSG00000169682	SPNS1	0.72	-0.48	0.00
ENSG00000249631	AC005699	0.00	-9.23	0.00
ENSG00000234685	NUS1P2	0.51	-0.98	0.00
ENSG00000169258	GPRIN1	0.77	-0.37	0.00
ENSG00000171208	NETO2	0.78	-0.37	0.00
ENSG00000245112	SMARCA5-AS1	0.51	-0.97	0.00
ENSG00000137073	UBAP2	0.74	-0.43	0.00
ENSG00000116560	SFPQ	0.83	-0.28	0.00
ENSG00000107518	ATRNL1	0.36	-1.46	0.00
ENSG00000188878	FBF1	0.73	-0.46	0.00
ENSG00000008405	CRY1	0.69	-0.53	0.00
ENSG00000155561	NUP205	0.79	-0.34	0.00
ENSG00000177707	NECTIN3	0.63	-0.67	0.00
ENSG00000130559	CAMSAP1	0.75	-0.42	0.00
ENSG00000172345	STARD5	0.40	-1.34	0.00
ENSG00000079134	THOC1	0.72	-0.48	0.00
ENSG00000177302	TOP3A	0.76	-0.40	0.00
ENSG00000185339	TCN2	0.60	-0.75	0.00
ENSG00000198826	ARHGAP11A	0.68	-0.56	0.00
ENSG00000164754	RAD21	0.77	-0.37	0.00
ENSG00000112796	ENPP5	0.46	-1.12	0.00
ENSG00000107758	PPP3CB	0.78	-0.37	0.00
ENSG00000184611	KCNH7	0.04	-4.60	0.00
ENSG00000164879	CA3	0.77	-0.39	0.00
ENSG00000138617	PARP16	0.55	-0.86	0.00
ENSG00000095539	SEMA4G	0.64	-0.65	0.00
ENSG00000118162	KPTN	0.60	-0.73	0.00
ENSG00000250376	AC093720	0.00	-11.92	0.00
ENSG00000152240	HAUS1	0.64	-0.65	0.00
ENSG00000183889	AC138969	0.59	-0.76	0.00
ENSG00000164794	KCNV1	0.53	-0.93	0.00
ENSG00000160551	TAOK1	0.78	-0.36	0.00
ENSG00000132466	ANKRD17	0.81	-0.30	0.00
ENSG00000183495	EP400	0.80	-0.32	0.00
ENSG00000106144	CASP2	0.56	-0.85	0.00
ENSG00000130522	JUND	0.79	-0.34	0.00
ENSG00000107929	LARP4B	0.80	-0.33	0.00
ENSG00000095002	MSH2	0.71	-0.48	0.00
ENSG00000177694	NAALADL2	0.22	-2.16	0.00
ENSG00000094914	AAAS	0.78	-0.36	0.00
ENSG00000166272	WBP1L	0.78	-0.35	0.00
ENSG00000164053	ATRIP	0.60	-0.73	0.00
ENSG00000186522	SEPTIN10	0.74	-0.44	0.00
ENSG00000182957	SPATA13	0.46	-1.12	0.00
ENSG00000167702	KIFC2	0.65	-0.63	0.00
ENSG00000126243	LRFN3	0.62	-0.70	0.00
ENSG00000223891	OSER1-DT	0.61	-0.70	0.00
ENSG00000198089	SFI1	0.66	-0.60	0.00
ENSG00000166046	TCP11L2	0.58	-0.78	0.00
ENSG00000147202	DIAPH2	0.62	-0.70	0.00
ENSG00000121671	CRY2	0.63	-0.67	0.00
ENSG00000136720	HS6ST1	0.69	-0.54	0.00
ENSG00000044459	CNTLN	0.54	-0.88	0.00
ENSG00000132002	DNAJB1	0.80	-0.32	0.00

ENSG00000044090	CUL7	0.78	-0.36	0.00
ENSG00000164087	POC1A	0.70	-0.52	0.00
ENSG00000084234	APLP2	0.80	-0.32	0.00
ENSG00000158062	UBXN11	0.64	-0.64	0.00
ENSG00000136111	TBC1D4	0.70	-0.52	0.00
ENSG00000177189	RPS6KA3	0.73	-0.45	0.00
ENSG00000261061	AC092718	0.56	-0.83	0.00
ENSG00000185630	PBX1	0.73	-0.46	0.00
ENSG00000171357	LURAP1	0.13	-2.89	0.00
ENSG00000026036	RTEL1-TNFRSF6B	0.41	-1.29	0.00
ENSG00000181827	RFX7	0.70	-0.52	0.00
ENSG00000147854	UHRF2	0.69	-0.54	0.00
ENSG00000117009	KMO	0.37	-1.42	0.00
ENSG00000266445	NARF-AS1	0.00	-11.72	0.00
ENSG00000119707	RBM25	0.78	-0.35	0.00
ENSG00000141577	CEP131	0.74	-0.43	0.00
ENSG00000228716	DHFR	0.61	-0.72	0.00
ENSG00000075702	WDR62	0.74	-0.43	0.00
ENSG00000187736	NHEJ1	0.70	-0.52	0.00
ENSG00000006607	FARP2	0.76	-0.40	0.00
ENSG00000125458	NT5C	0.65	-0.63	0.00
ENSG00000120896	SORBS3	0.77	-0.37	0.00
ENSG00000149636	DSN1	0.69	-0.53	0.00
ENSG00000188092	GPR89B	0.51	-0.98	0.00
ENSG00000235852	AC005540	0.65	-0.62	0.00
ENSG00000163617	CCDC191	0.56	-0.84	0.00
ENSG00000101596	SMCHD1	0.70	-0.51	0.00
ENSG00000113448	PDE4D	0.51	-0.98	0.00
ENSG00000081026	MAGI3	0.59	-0.76	0.00
ENSG00000078699	CBFA2T2	0.70	-0.51	0.00
ENSG00000213186	TRIM59	0.50	-1.01	0.00
ENSG00000163041	H3-3A	0.74	-0.44	0.00
ENSG00000145495	MARCHF6	0.77	-0.37	0.00
ENSG00000132434	LANCL2	0.75	-0.42	0.00
ENSG00000175505	CLCF1	0.74	-0.44	0.00
ENSG00000129691	ASH2L	0.78	-0.37	0.00
ENSG00000127483	HP1BP3	0.80	-0.33	0.00
ENSG00000183496	MEX3B	0.26	-1.93	0.00
ENSG00000137251	TINAG	0.19	-2.43	0.00
ENSG00000176155	CCDC57	0.79	-0.34	0.00
ENSG00000167173	C15orf39	0.61	-0.72	0.00
ENSG00000128266	GNAZ	0.23	-2.14	0.00
ENSG00000128708	HAT1	0.77	-0.38	0.00
ENSG00000135541	AHI1	0.55	-0.85	0.00
ENSG00000119725	ZNF410	0.68	-0.56	0.00
ENSG00000140836	ZFHX3	0.76	-0.40	0.00
ENSG00000197275	RAD54B	0.66	-0.59	0.00
ENSG00000213886	UBD	0.20	-2.33	0.00
ENSG00000125827	TMX4	0.76	-0.40	0.00
ENSG00000175928	LRRN1	0.61	-0.70	0.00
ENSG00000146039	SLC17A4	0.00	-9.19	0.00
ENSG00000162337	LRP5	0.75	-0.41	0.00
ENSG00000100629	CEP128	0.43	-1.23	0.00
ENSG00000167306	MYO5B	0.62	-0.69	0.00
ENSG00000054219	LY75	0.63	-0.68	0.00
ENSG00000130829	DUSP9	0.36	-1.46	0.00
ENSG00000141480	ARRB2	0.71	-0.50	0.00
ENSG00000165186	PTCHD1	0.53	-0.91	0.00
ENSG00000141519	CCDC40	0.60	-0.73	0.00
ENSG00000264538	SUZ12P1	0.57	-0.81	0.00
ENSG00000115993	TRAK2	0.77	-0.37	0.00

ENSG00000164323	CFAP97	0.72	-0.47	0.00
ENSG00000135048	CEMIP2	0.79	-0.34	0.00
ENSG00000287126	AC108488	0.37	-1.44	0.00
ENSG00000053371	AKR7A2	0.69	-0.54	0.00
ENSG00000140450	ARRDC4	0.65	-0.63	0.00
ENSG00000180228	PRKRA	0.74	-0.44	0.00
ENSG00000105677	TMEM147	0.76	-0.40	0.00
ENSG00000178440	TIMM23B-AGAP6	0.34	-1.56	0.00
ENSG00000054793	ATP9A	0.79	-0.35	0.00
ENSG00000104518	GSDMD	0.68	-0.56	0.00
ENSG00000103257	SLC7A5	0.82	-0.29	0.00
ENSG00000177459	ERICH5	0.30	-1.72	0.00
ENSG00000215105	TTC3P1	0.29	-1.79	0.00
ENSG00000186193	SAPCD2	0.72	-0.47	0.00
ENSG00000157106	SMG1	0.77	-0.37	0.00
ENSG00000160310	PRMT2	0.79	-0.34	0.00
ENSG00000162745	OLFML2B	0.25	-1.99	0.00
ENSG00000176244	ACBD7	0.56	-0.85	0.00
ENSG00000143149	ALDH9A1	0.80	-0.32	0.00
ENSG00000236609	ZNF853	0.13	-2.89	0.00
ENSG00000277027	RMRP	0.01	-6.33	0.00
ENSG00000169398	PTK2	0.73	-0.45	0.00
ENSG00000116830	TTF2	0.71	-0.49	0.00
ENSG00000100479	POLE2	0.52	-0.95	0.00
ENSG00000149256	TENM4	0.48	-1.06	0.00
ENSG00000168066	SF1	0.82	-0.28	0.00
ENSG00000132846	ZBED3	0.65	-0.62	0.00
ENSG00000162878	PKDCC	0.61	-0.71	0.00
ENSG00000112249	ASCC3	0.77	-0.37	0.00
ENSG00000149308	NPAT	0.56	-0.83	0.00
ENSG00000074416	MGLL	0.83	-0.27	0.00
ENSG00000261221	ZNF865	0.73	-0.45	0.00
ENSG00000138400	MDH1B	0.46	-1.12	0.00
ENSG00000026103	FAS	0.69	-0.54	0.00
ENSG00000106004	HOXA5	0.66	-0.59	0.00
ENSG00000013288	MAN2B2	0.78	-0.35	0.00
ENSG00000092439	TRPM7	0.70	-0.51	0.00
ENSG00000113360	DROSHA	0.78	-0.35	0.00
ENSG00000101049	SGK2	0.64	-0.63	0.00
ENSG00000138162	TACC2	0.73	-0.46	0.00
ENSG00000076826	CAMSAP3	0.36	-1.46	0.00
ENSG00000117155	SSX2IP	0.65	-0.63	0.00
ENSG00000197555	SIPA1L1	0.61	-0.70	0.00
ENSG00000181523	SGSH	0.74	-0.43	0.00
ENSG00000046651	OFD1	0.69	-0.53	0.00
ENSG00000114315	HES1	0.56	-0.85	0.00
ENSG00000181588	MEX3D	0.68	-0.56	0.00
ENSG00000121858	TNFSF10	0.69	-0.53	0.00
ENSG00000137571	SLCO5A1	0.37	-1.43	0.00
ENSG00000095787	WAC	0.80	-0.32	0.00
ENSG00000168818	STX18	0.77	-0.38	0.00
ENSG00000234420	ZNF37BP	0.55	-0.85	0.00
ENSG00000197417	SHPK	0.73	-0.45	0.00
ENSG00000185515	BRCC3	0.64	-0.64	0.00
ENSG00000225329	LHFPL3-AS2	0.00	-10.00	0.00
ENSG00000120913	PDLIM2	0.63	-0.66	0.00
ENSG00000109819	PPARGC1A	0.60	-0.75	0.00
ENSG00000188493	C19orf54	0.72	-0.47	0.00
ENSG00000151693	ASAP2	0.68	-0.55	0.00
ENSG00000125447	GGA3	0.68	-0.56	0.00
ENSG00000113391	FAM172A	0.61	-0.72	0.00

ENSG00000110315	RNF141	0.75	-0.41	0.00
ENSG00000185201	IFITM2	0.38	-1.41	0.00
ENSG00000106976	DNM1	0.70	-0.51	0.00
ENSG00000255468	AP001107	0.23	-2.13	0.00
ENSG00000049246	PER3	0.64	-0.65	0.00
ENSG00000233610	LINC00462	0.03	-5.07	0.00
ENSG00000170608	FOXA3	0.00	-10.30	0.00
ENSG00000185504	FAAP100	0.77	-0.38	0.00
ENSG00000063587	ZNF275	0.75	-0.41	0.00
ENSG00000071246	VASH1	0.23	-2.12	0.00
ENSG00000074657	ZNF532	0.60	-0.73	0.00
ENSG00000040531	CTNS	0.66	-0.59	0.00
ENSG00000120800	UTP20	0.80	-0.32	0.00
ENSG00000223770	CACNA2D1-AS1	0.04	-4.52	0.00
ENSG00000276975	HYDIN2	0.28	-1.85	0.00
ENSG00000125648	SLC25A23	0.79	-0.34	0.00
ENSG00000196323	ZBTB44	0.57	-0.81	0.00
ENSG00000114999	TTL	0.80	-0.32	0.00
ENSG00000167733	HSD11B1L	0.59	-0.76	0.00
ENSG00000198752	CDC42BPB	0.82	-0.29	0.00
ENSG00000104522	TSTA3	0.74	-0.43	0.00
ENSG00000140367	UBE2Q2	0.73	-0.46	0.00
ENSG00000242732	RTL5	0.40	-1.31	0.00
ENSG00000102221	JADE3	0.71	-0.49	0.00
ENSG00000279453	Z99129	0.44	-1.17	0.00
ENSG00000136630	HLX	0.67	-0.58	0.00
ENSG00000170430	MGMT	0.79	-0.35	0.00
ENSG00000225864	AL645939	0.19	-2.39	0.00
ENSG00000203668	CHML	0.75	-0.42	0.00
ENSG00000055917	PUM2	0.72	-0.48	0.00
ENSG00000134072	CAMK1	0.71	-0.50	0.00
ENSG00000105662	CRTC1	0.74	-0.44	0.00
ENSG00000162415	ZSWIM5	0.00	-8.68	0.00
ENSG00000196922	ZNF252P	0.64	-0.64	0.00
ENSG00000169733	RFNG	0.71	-0.50	0.00
ENSG00000119596	YLPM1	0.80	-0.32	0.00
ENSG00000144959	NCEH1	0.81	-0.30	0.00
ENSG00000132017	DCAF15	0.74	-0.43	0.00
ENSG00000155893	PXYLP1	0.39	-1.36	0.00
ENSG00000160584	SIK3	0.77	-0.38	0.00
ENSG00000215790	SLC35E2A	0.77	-0.38	0.00
ENSG00000160959	LRRC14	0.78	-0.35	0.00
ENSG00000205593	DENND6B	0.33	-1.60	0.00
ENSG00000110013	SIAE	0.73	-0.45	0.00
ENSG00000135525	MAP7	0.50	-1.00	0.00
ENSG00000007541	PIGQ	0.59	-0.76	0.00
ENSG00000148120	AOPEP	0.72	-0.48	0.00
ENSG00000127603	MACF1	0.80	-0.32	0.00
ENSG00000283563	AC098650	0.00	-9.56	0.00
ENSG00000067836	ROGDI	0.67	-0.57	0.00
ENSG00000166912	MTMR10	0.58	-0.79	0.00
ENSG00000142875	PRKACB	0.67	-0.58	0.00
ENSG00000117748	RPA2	0.76	-0.40	0.00
ENSG00000187231	SESTD1	0.62	-0.70	0.00
ENSG00000151882	CCL28	0.43	-1.23	0.00
ENSG00000125618	PAX8	0.84	-0.26	0.00
ENSG00000166326	TRIM44	0.83	-0.27	0.00
ENSG00000239521	CASTOR3	0.61	-0.71	0.00
ENSG00000117448	AKR1A1	0.80	-0.33	0.00
ENSG00000105323	HNRNPUL1	0.82	-0.29	0.00
ENSG00000174938	SEZ6L2	0.74	-0.44	0.00

ENSG00000144040	SFXN5	0.75	-0.42	0.00
ENSG00000174358	SLC6A19	0.03	-5.05	0.00
ENSG00000138119	MYOF	0.83	-0.26	0.00
ENSG00000140545	MFGE8	0.74	-0.43	0.00
ENSG00000133069	TMCC2	0.66	-0.59	0.00
ENSG00000232859	LYRM9	0.49	-1.02	0.00
ENSG00000132824	SERINC3	0.82	-0.28	0.00
ENSG00000163507	CIP2A	0.71	-0.50	0.00
ENSG00000185477	GPRIN3	0.35	-1.53	0.00
ENSG00000121741	ZMYM2	0.76	-0.40	0.00
ENSG00000148719	DNAJB12	0.79	-0.34	0.00
ENSG00000163624	CDS1	0.38	-1.40	0.00
ENSG00000079841	RIMS1	0.19	-2.39	0.00
ENSG00000179361	ARID3B	0.56	-0.85	0.00
ENSG00000178467	P4HTM	0.61	-0.72	0.00
ENSG00000152492	CCDC50	0.80	-0.32	0.00
ENSG00000235501	AC105942	0.41	-1.30	0.00
ENSG00000238228	OR7E7P	0.12	-3.01	0.00
ENSG00000140688	RUSF1	0.71	-0.50	0.00
ENSG00000004866	ST7	0.73	-0.45	0.00
ENSG00000198075	SULT1C4	0.00	-9.48	0.00
ENSG00000143164	DCAF6	0.81	-0.30	0.00
ENSG00000035928	RFC1	0.79	-0.34	0.00
ENSG00000117016	RIMS3	0.39	-1.34	0.00
ENSG00000144115	THNSL2	0.65	-0.61	0.00
ENSG00000277053	GTF2IP1	0.69	-0.53	0.00
ENSG00000213465	ARL2	0.77	-0.38	0.00
ENSG00000126215	XRCC3	0.71	-0.49	0.00
ENSG00000124164	VAPB	0.62	-0.69	0.00
ENSG00000120327	PCDHB14	0.28	-1.83	0.00
ENSG00000151623	NR3C2	0.49	-1.02	0.00
ENSG00000123416	TUBA1B	0.77	-0.37	0.00
ENSG00000188647	PTAR1	0.75	-0.42	0.00
ENSG00000007168	PAFAH1B1	0.67	-0.59	0.00
ENSG00000166398	GARRE1	0.73	-0.46	0.00
ENSG00000128567	PODXL	0.42	-1.25	0.00
ENSG00000046647	GEMIN8	0.65	-0.63	0.00
ENSG00000167711	SERPINF2	0.33	-1.60	0.00
ENSG00000068383	INPP5A	0.76	-0.39	0.00
ENSG00000167202	TBC1D2B	0.79	-0.33	0.00
ENSG00000227036	LINC00511	0.58	-0.78	0.00
ENSG00000188596	CFAP54	0.24	-2.05	0.00
ENSG00000060656	PTPRU	0.60	-0.75	0.00
ENSG00000171798	KNDC1	0.00	-8.34	0.00
ENSG00000223764	LINC02593	0.01	-6.29	0.00
ENSG00000123570	RAB9B	0.59	-0.75	0.00
ENSG00000288640	AC005192	0.00	-9.54	0.00
ENSG00000230535	BASP1P1	0.16	-2.61	0.00
ENSG00000103647	CORO2B	0.32	-1.63	0.00
ENSG00000175318	GRAMD2A	0.05	-4.36	0.00
ENSG00000261611	AC010547	0.17	-2.60	0.00
ENSG00000006756	ARSD	0.73	-0.44	0.00
ENSG00000159423	ALDH4A1	0.75	-0.42	0.00
ENSG00000230438	SERPINB9P1	0.57	-0.81	0.00
ENSG00000138286	FAM149B1	0.73	-0.46	0.00
ENSG00000148158	SNX30	0.72	-0.47	0.00
ENSG00000099284	MACROH2A2	0.67	-0.58	0.00
ENSG00000142102	PGGHG	0.63	-0.68	0.00
ENSG00000164104	HMGB2	0.65	-0.62	0.00
ENSG00000177791	MYOZ1	0.00	-10.77	0.00
ENSG00000231013	SCTR-AS1	0.00	-9.50	0.00

ENSG00000132405	TBC1D14	0.82	-0.28	0.00
ENSG00000137817	PARP6	0.73	-0.46	0.00
ENSG00000166889	PATL1	0.80	-0.32	0.00
ENSG00000123374	CDK2	0.69	-0.54	0.00
ENSG00000177425	PAWR	0.76	-0.40	0.00
ENSG00000122547	EEPD1	0.68	-0.55	0.00
ENSG00000135766	EGLN1	0.80	-0.33	0.00
ENSG00000142798	HSPG2	0.82	-0.29	0.00
ENSG00000145949	MYLK4	0.23	-2.15	0.00
ENSG00000048707	VPS13D	0.76	-0.39	0.00
ENSG00000198246	SLC29A3	0.42	-1.25	0.00
ENSG00000214113	LYRM4	0.63	-0.66	0.00
ENSG00000140025	EFCAB11	0.60	-0.75	0.00
ENSG00000172071	EIF2AK3	0.67	-0.57	0.00
ENSG00000130726	TRIM28	0.81	-0.31	0.00
ENSG00000162341	TPCN2	0.71	-0.50	0.00
ENSG00000075790	BCAP29	0.55	-0.85	0.00
ENSG00000165023	DIRAS2	0.10	-3.30	0.00
ENSG00000007944	MYLIP	0.59	-0.76	0.00
ENSG00000185344	ATP6V0A2	0.73	-0.45	0.00
ENSG00000136848	DAB2IP	0.44	-1.19	0.00
ENSG00000170037	CNTROB	0.77	-0.37	0.00
ENSG00000104133	SPG11	0.69	-0.54	0.00
ENSG00000131446	MGAT1	0.83	-0.26	0.00
ENSG00000221829	FANCG	0.77	-0.37	0.00
ENSG00000096968	JAK2	0.57	-0.81	0.00
ENSG00000175274	TP53I11	0.57	-0.81	0.00
ENSG00000153044	CENPH	0.69	-0.53	0.00
ENSG00000056097	ZFR	0.80	-0.33	0.00
ENSG00000141446	ESCO1	0.64	-0.65	0.00
ENSG00000139631	CSAD	0.63	-0.66	0.00
ENSG00000181856	SLC2A4	0.05	-4.32	0.00
ENSG00000104835	SARS2	0.74	-0.44	0.00
ENSG00000187624	C17orf97	0.45	-1.16	0.00
ENSG00000164976	MYORG	0.55	-0.87	0.00
ENSG00000128274	A4GALT	0.59	-0.77	0.00
ENSG00000139083	ETV6	0.80	-0.31	0.00
ENSG00000130787	HIP1R	0.78	-0.37	0.00
ENSG00000178971	CTC1	0.70	-0.51	0.00
ENSG00000088280	ASAP3	0.71	-0.49	0.00
ENSG00000094975	SUCO	0.75	-0.42	0.00
ENSG00000064989	CALCRL	0.43	-1.20	0.00
ENSG00000104886	PLEKHJ1	0.64	-0.65	0.00
ENSG00000128908	INO80	0.75	-0.41	0.00
ENSG00000149476	TKFC	0.71	-0.49	0.00
ENSG00000135913	USP37	0.60	-0.73	0.00
ENSG00000133624	ZNF767P	0.67	-0.57	0.00
ENSG00000085788	DDHD2	0.66	-0.61	0.00
ENSG00000185420	SMYD3	0.72	-0.47	0.00
ENSG00000196498	NCOR2	0.84	-0.26	0.00
ENSG00000235269	LINC02331	0.00	-9.87	0.00
ENSG00000168268	NT5DC2	0.76	-0.39	0.00
ENSG00000005206	SPPL2B	0.73	-0.45	0.00
ENSG00000243302	AC018638	0.56	-0.83	0.00
ENSG00000078403	MLLT10	0.71	-0.49	0.00
ENSG00000104375	STK3	0.71	-0.49	0.00
ENSG00000125676	THOC2	0.75	-0.42	0.00
ENSG00000181744	DIPK2A	0.69	-0.54	0.00
ENSG00000151461	UPF2	0.72	-0.48	0.00
ENSG00000130695	CEP85	0.73	-0.46	0.00
ENSG00000160211	G6PD	0.84	-0.25	0.00

ENSG00000223658	C1GALT1C1L	0.45	-1.16	0.00
ENSG00000286847	AC007036	0.00	-10.07	0.00
ENSG00000170085	SIMC1	0.64	-0.65	0.00
ENSG00000131242	RAB11FIP4	0.22	-2.16	0.00
ENSG00000163104	SMARCAD1	0.75	-0.41	0.00
ENSG00000168172	HOOK3	0.76	-0.39	0.00
ENSG00000099849	RASSF7	0.62	-0.68	0.00
ENSG00000024422	EHD2	0.77	-0.38	0.00
ENSG00000052841	TTC17	0.81	-0.30	0.00
ENSG00000272768	AC004854	0.11	-3.22	0.00
ENSG00000109756	RAPGEF2	0.76	-0.40	0.00
ENSG00000110844	PRPF40B	0.72	-0.47	0.00
ENSG00000011295	TTC19	0.57	-0.80	0.00
ENSG00000174516	PELI3	0.62	-0.70	0.00
ENSG00000113580	NR3C1	0.80	-0.32	0.00
ENSG00000183092	BEGAIN	0.26	-1.93	0.00
ENSG00000183283	DAZAP2	0.69	-0.54	0.00
ENSG00000006704	GTF2IRD1	0.78	-0.37	0.00
ENSG00000137285	TUBB2B	0.68	-0.55	0.00
ENSG00000205959	AC105345	0.00	-8.88	0.00
ENSG00000085063	CD59	0.77	-0.38	0.00
ENSG00000140326	CDAN1	0.74	-0.43	0.00
ENSG00000197061	H4C3	0.04	-4.75	0.00
ENSG00000130545	CRB3	0.18	-2.49	0.00
ENSG00000213064	SFT2D2	0.83	-0.28	0.00
ENSG00000087586	AURKA	0.82	-0.29	0.00
ENSG00000103126	AXIN1	0.75	-0.42	0.00
ENSG00000172059	KLF11	0.70	-0.52	0.00
ENSG00000109323	MANBA	0.72	-0.47	0.00
ENSG00000124486	USP9X	0.82	-0.28	0.00
ENSG00000166333	ILK	0.82	-0.29	0.00
ENSG00000103540	CCP110	0.70	-0.52	0.00
ENSG00000101104	PABPC1L	0.74	-0.44	0.00
ENSG00000131788	PIAS3	0.79	-0.33	0.00
ENSG00000141367	CLTC	0.84	-0.26	0.00
ENSG00000149925	ALDOA	0.85	-0.23	0.00
ENSG00000156642	NPTN	0.82	-0.28	0.00
ENSG00000129757	CDKN1C	0.32	-1.62	0.00
ENSG00000141068	KSR1	0.71	-0.50	0.00
ENSG00000128944	KNSTRN	0.71	-0.49	0.00
ENSG00000248801	C8orf34-AS1	0.05	-4.47	0.00
ENSG00000103145	HCFC1R1	0.71	-0.49	0.00
ENSG00000161692	DBF4B	0.73	-0.46	0.00
ENSG00000111581	NUP107	0.78	-0.37	0.00
ENSG00000230606	AC092683	0.42	-1.26	0.00
ENSG00000270659	AC079610	0.00	-11.73	0.00
ENSG00000157933	SKI	0.83	-0.28	0.00
ENSG00000163655	GMPS	0.84	-0.26	0.00
ENSG00000101972	STAG2	0.78	-0.35	0.00
ENSG00000134253	TRIM45	0.66	-0.60	0.00
ENSG00000153237	CCDC148	0.27	-1.88	0.00
ENSG00000108187	PBLD	0.58	-0.78	0.00
ENSG00000186472	PCLO	0.47	-1.08	0.00
ENSG00000197879	MYO1C	0.82	-0.28	0.00
ENSG00000073712	FERMT2	0.73	-0.45	0.00
ENSG00000014914	MTMR11	0.64	-0.64	0.00
ENSG00000047365	ARAP2	0.60	-0.74	0.00
ENSG00000137726	FXYD6	0.16	-2.63	0.00
ENSG00000110195	FOLR1	0.36	-1.46	0.00
ENSG00000134030	CTIF	0.72	-0.47	0.00
ENSG00000269190	FBXO17	0.76	-0.40	0.00

ENSG00000123352	SPATS2	0.76	-0.40	0.00
ENSG00000025796	SEC63	0.76	-0.39	0.00
ENSG00000105738	SIPA1L3	0.80	-0.33	0.00
ENSG00000160216	AGPAT3	0.80	-0.32	0.00
ENSG00000158711	ELK4	0.77	-0.38	0.00
ENSG00000121057	AKAP1	0.82	-0.29	0.00
ENSG00000009954	BAZ1B	0.81	-0.31	0.00
ENSG00000149716	LTO1	0.57	-0.80	0.00
ENSG00000161682	FAM171A2	0.48	-1.05	0.00
ENSG00000184719	RNLS	0.69	-0.54	0.00
ENSG00000143919	CAMKMT	0.55	-0.86	0.00
ENSG00000248383	PCDHAC1	0.05	-4.19	0.00
ENSG00000270055	AC127502	0.54	-0.88	0.00
ENSG00000172638	EFEMP2	0.65	-0.61	0.00
ENSG00000007384	RHBDF1	0.63	-0.67	0.00
ENSG00000138439	FAM117B	0.64	-0.63	0.00
ENSG00000176208	ATAD5	0.62	-0.68	0.00
ENSG00000167397	VKORC1	0.74	-0.43	0.00
ENSG00000114805	PLCH1	0.65	-0.63	0.00
ENSG00000167740	CYB5D2	0.75	-0.42	0.00
ENSG00000119636	BBOF1	0.42	-1.25	0.00
ENSG00000189127	ANKRD34B	0.16	-2.67	0.00
ENSG00000165507	DEPP1	0.74	-0.44	0.00
ENSG00000144278	GALNT13	0.68	-0.55	0.00
ENSG00000133739	LRRCC1	0.52	-0.95	0.00
ENSG00000034533	ASTE1	0.65	-0.63	0.00
ENSG00000172201	ID4	0.00	-9.05	0.00
ENSG00000123975	CKS2	0.82	-0.29	0.00
ENSG00000073008	PVR	0.76	-0.40	0.00
ENSG00000131591	C1orf159	0.68	-0.55	0.00
ENSG00000112039	FANCE	0.67	-0.57	0.00
ENSG00000146576	C7orf26	0.71	-0.49	0.00
ENSG00000130803	ZNF317	0.76	-0.40	0.00
ENSG00000133195	SLC39A11	0.76	-0.39	0.00
ENSG00000101974	ATP11C	0.77	-0.38	0.00
ENSG00000168765	GSTM4	0.61	-0.72	0.00
ENSG00000248763	AC111000	0.26	-1.95	0.00
ENSG00000128512	DOCK4	0.77	-0.38	0.00
ENSG00000157343	ARMC12	0.00	-10.52	0.00
ENSG00000159674	SPON2	0.33	-1.62	0.00
ENSG00000135144	DTX1	0.17	-2.53	0.00
ENSG00000280206	AC026401	0.61	-0.72	0.00
ENSG00000009830	POMT2	0.77	-0.38	0.00
ENSG00000002586	CD99	0.79	-0.35	0.00
ENSG00000075886	TUBA3D	0.18	-2.46	0.00
ENSG00000254996	ANKHD1-EIF4EBP3	0.77	-0.38	0.00
ENSG00000124831	LRRFIP1	0.81	-0.30	0.00
ENSG00000165156	ZHX1	0.57	-0.81	0.00
ENSG00000151233	GXYLT1	0.73	-0.45	0.00
ENSG00000257621	PSMA3-AS1	0.67	-0.58	0.00
ENSG00000238103	RPL9P7	0.76	-0.39	0.00
ENSG00000156136	DCK	0.80	-0.32	0.00
ENSG00000100997	ABHD12	0.74	-0.43	0.00
ENSG00000135643	KCNMB4	0.45	-1.17	0.00
ENSG00000165795	NDRG2	0.37	-1.43	0.00
ENSG00000204851	PNMA8B	0.37	-1.44	0.00
ENSG00000198925	ATG9A	0.75	-0.42	0.00
ENSG00000244257	PKD1P1	0.77	-0.38	0.00
ENSG00000196562	SULF2	0.37	-1.43	0.00
ENSG00000185986	SDHAP3	0.42	-1.27	0.00
ENSG00000278771	RN7SL3	0.08	-3.66	0.00

ENSG00000174669	SLC29A2	0.26	-1.93	0.00
ENSG00000236064	AL109946	0.16	-2.69	0.00
ENSG00000132424	PNISR	0.78	-0.36	0.00
ENSG00000267480	AP001542	0.41	-1.28	0.00
ENSG00000133424	LARGE1	0.28	-1.86	0.00
ENSG00000205250	E2F4	0.79	-0.34	0.00
ENSG00000165406	MARCHF8	0.73	-0.46	0.00
ENSG00000138688	KIAA1109	0.70	-0.51	0.00
ENSG00000115486	GGCX	0.82	-0.29	0.00
ENSG00000152939	MARVELD2	0.59	-0.77	0.00
ENSG00000173898	SPTBN2	0.29	-1.80	0.00
ENSG00000159685	CHCHD6	0.78	-0.37	0.00
ENSG00000181929	PRKAG1	0.81	-0.30	0.00
ENSG00000078369	GNB1	0.84	-0.25	0.00
ENSG00000141753	IGFBP4	0.61	-0.71	0.00
ENSG00000101290	CDS2	0.81	-0.30	0.00
ENSG00000228791	THRB-AS1	0.00	-8.23	0.00
ENSG00000105647	PIK3R2	0.69	-0.53	0.00
ENSG00000163697	APBB2	0.82	-0.28	0.00
ENSG00000030419	IKZF2	0.41	-1.28	0.00
ENSG00000143797	MBOAT2	0.78	-0.36	0.00
ENSG00000206341	HLA-H	0.53	-0.92	0.00
ENSG00000204764	RANBP17	0.60	-0.74	0.00
ENSG00000074755	ZZEF1	0.82	-0.28	0.00
ENSG00000198353	HOXC4	0.23	-2.12	0.00
ENSG00000175265	GOLGA8A	0.62	-0.69	0.00
ENSG00000198331	HYLS1	0.67	-0.58	0.00
ENSG00000150687	PRSS23	0.58	-0.80	0.00
ENSG00000116199	FAM20B	0.82	-0.28	0.00
ENSG00000179456	ZBTB18	0.78	-0.36	0.00
ENSG00000184992	BRI3BP	0.76	-0.40	0.00
ENSG00000103319	EEF2K	0.81	-0.31	0.00
ENSG00000176387	HSD11B2	0.19	-2.37	0.00
ENSG00000198162	MAN1A2	0.81	-0.30	0.00
ENSG00000276772	AC025271	0.00	-10.24	0.00
ENSG00000284048	AC073111	0.21	-2.27	0.00
ENSG00000204624	DISP3	0.00	-8.27	0.00
ENSG00000284308	C2orf81	0.37	-1.45	0.00
ENSG00000169710	FASN	0.84	-0.25	0.00
ENSG00000125817	CENPB	0.83	-0.26	0.00
ENSG00000176714	CCDC121	0.35	-1.50	0.01
ENSG00000092096	SLC22A17	0.38	-1.39	0.01
ENSG00000139178	C1RL	0.77	-0.38	0.01
ENSG00000178921	PFAS	0.78	-0.36	0.01
ENSG00000075624	ACTB	0.78	-0.37	0.01
ENSG00000164929	BAALC	0.38	-1.41	0.01
ENSG00000178977	LINC00324	0.15	-2.72	0.01
ENSG00000155957	TMBIM4	0.74	-0.43	0.01
ENSG00000152404	CWF19L2	0.57	-0.81	0.01
ENSG00000182054	IDH2	0.82	-0.29	0.01
ENSG00000159214	CCDC24	0.60	-0.73	0.01
ENSG00000119938	PPP1R3C	0.66	-0.60	0.01
ENSG00000205078	SYCE1L	0.53	-0.91	0.01
ENSG00000123636	BAZ2B	0.72	-0.48	0.01
ENSG00000169760	NLGN1	0.38	-1.40	0.01
ENSG00000111679	PTPN6	0.76	-0.39	0.01
ENSG00000120756	PLS1	0.67	-0.59	0.01
ENSG00000106991	ENG	0.83	-0.27	0.01
ENSG00000132376	INPP5K	0.78	-0.36	0.01
ENSG00000030066	NUP160	0.82	-0.29	0.01
ENSG00000171056	SOX7	0.00	-9.16	0.01

ENSG00000173557	FAM166C	0.00	-9.52	0.01
ENSG00000164096	C4orf3	0.82	-0.29	0.01
ENSG00000088387	DOCK9	0.77	-0.39	0.01
ENSG00000183763	TRAIP	0.63	-0.67	0.01
ENSG00000114423	CBLB	0.69	-0.55	0.01
ENSG00000124788	ATXN1	0.70	-0.51	0.01
ENSG00000249437	NAIP	0.44	-1.20	0.01
ENSG00000171877	FRMD5	0.49	-1.02	0.01
ENSG00000162913	OBSCN-AS1	0.43	-1.22	0.01
ENSG00000187164	SHTN1	0.78	-0.36	0.01
ENSG00000117868	ESYT2	0.83	-0.27	0.01
ENSG00000033170	FUT8	0.71	-0.49	0.01
ENSG00000176915	ANKLE2	0.84	-0.26	0.01
ENSG00000135378	PRRG4	0.61	-0.72	0.01
ENSG00000065717	TLE2	0.17	-2.54	0.01
ENSG00000177463	NR2C2	0.81	-0.30	0.01
ENSG00000259250	AC018904	0.12	-3.09	0.01
ENSG00000164307	ERAP1	0.72	-0.47	0.01
ENSG00000201185	RNA5SP202	0.03	-4.99	0.01
ENSG00000176095	IP6K1	0.81	-0.30	0.01
ENSG00000256663	AC112777	0.62	-0.69	0.01
ENSG00000263956	NBPF11	0.71	-0.49	0.01
ENSG00000165821	SALL2	0.72	-0.48	0.01
ENSG00000066777	ARFGEF1	0.78	-0.35	0.01
ENSG00000140153	WDR20	0.56	-0.84	0.01
ENSG00000173638	SLC19A1	0.79	-0.33	0.01
ENSG00000130177	CDC16	0.82	-0.29	0.01
ENSG00000178814	OPLAH	0.58	-0.78	0.01
ENSG00000121289	CEP89	0.77	-0.37	0.01
ENSG00000145284	SCD5	0.75	-0.42	0.01
ENSG00000178761	FAM219B	0.59	-0.76	0.01
ENSG00000162946	DISC1	0.73	-0.45	0.01
ENSG00000078687	TNRC6C	0.34	-1.55	0.01
ENSG00000175029	CTBP2	0.76	-0.40	0.01
ENSG00000279094	LINC01670	0.00	-10.94	0.01
ENSG00000279348	AC012513	0.54	-0.90	0.01
ENSG00000128050	PAICS	0.67	-0.58	0.01
ENSG00000058673	ZC3H11A	0.75	-0.41	0.01
ENSG00000099622	CIRBP	0.80	-0.33	0.01
ENSG00000173272	MZT2A	0.79	-0.35	0.01
ENSG00000048649	RSF1	0.75	-0.41	0.01
ENSG00000080298	RFX3	0.63	-0.66	0.01
ENSG00000135837	CEP350	0.76	-0.40	0.01
ENSG00000099917	MED15	0.81	-0.31	0.01
ENSG00000176771	NCKAP5	0.41	-1.30	0.01
ENSG00000109738	GLRB	0.64	-0.65	0.01
ENSG00000163635	ATXN7	0.60	-0.73	0.01
ENSG00000157540	DYRK1A	0.66	-0.61	0.01
ENSG00000181826	RELL1	0.74	-0.43	0.01
ENSG00000136856	SLC2A8	0.68	-0.56	0.01
ENSG00000112699	GMDS	0.62	-0.69	0.01
ENSG00000095015	MAP3K1	0.71	-0.49	0.01
ENSG00000184216	IRAK1	0.85	-0.23	0.01
ENSG00000108479	GALK1	0.76	-0.40	0.01
ENSG00000284946	AC068831	0.60	-0.73	0.01
ENSG00000177200	CHD9	0.74	-0.43	0.01
ENSG00000132744	ACY3	0.00	-10.38	0.01
ENSG00000112499	SLC22A2	0.00	-8.27	0.01
ENSG00000261594	TPBGL	0.00	-9.39	0.01
ENSG00000167130	DOLPP1	0.74	-0.43	0.01
ENSG00000196136	SERPINA3	0.00	-9.13	0.01

ENSG00000058063	ATP11B	0.81	-0.31	0.01
ENSG00000279177	CTBP2P9	0.36	-1.45	0.01
ENSG00000185158	LRRC37B	0.60	-0.73	0.01
ENSG00000080503	SMARCA2	0.81	-0.30	0.01
ENSG00000171446	KRT27	0.18	-2.47	0.01
ENSG00000099953	MMP11	0.16	-2.63	0.01
ENSG00000115548	KDM3A	0.79	-0.33	0.01
ENSG00000150867	PIP4K2A	0.82	-0.29	0.01
ENSG00000083444	PLOD1	0.86	-0.22	0.01
ENSG00000144199	FAHD2B	0.55	-0.86	0.01
ENSG00000168591	TMUB2	0.76	-0.40	0.01
ENSG00000124422	USP22	0.85	-0.24	0.01
ENSG00000267561	AC093155	0.00	-9.80	0.01
ENSG00000120942	UBIAD1	0.80	-0.33	0.01
ENSG00000164535	DAGLB	0.74	-0.43	0.01
ENSG00000117528	ABCD3	0.75	-0.41	0.01
ENSG00000269026	AC003006	0.00	-11.16	0.01
ENSG00000287146	AC000085	0.00	-11.14	0.01
ENSG00000196588	MRTFA	0.81	-0.31	0.01
ENSG00000077044	DGKD	0.82	-0.28	0.01
ENSG00000146063	TRIM41	0.81	-0.31	0.01
ENSG00000160446	ZDHHC12	0.78	-0.36	0.01
ENSG00000104219	ZDHHC2	0.80	-0.33	0.01
ENSG00000079739	PGM1	0.85	-0.23	0.01
ENSG00000179195	ZNF664	0.22	-2.20	0.01
ENSG00000095713	CRTAC1	0.00	-9.01	0.01
ENSG00000130943	PKDREJ	0.00	-7.87	0.01
ENSG00000141026	MED9	0.70	-0.51	0.01
ENSG00000149179	C11orf49	0.75	-0.41	0.01
ENSG00000196911	KPNA5	0.54	-0.89	0.01
ENSG00000160199	PKNOX1	0.71	-0.50	0.01
ENSG00000230426	LINC01036	0.52	-0.94	0.01
ENSG00000082996	RNF13	0.59	-0.76	0.01
ENSG00000109680	TBC1D19	0.66	-0.60	0.01
ENSG00000251003	ZFPM2-AS1	0.65	-0.63	0.01
ENSG00000259209	AC004943	0.01	-6.76	0.01
ENSG00000156414	TDRD9	0.72	-0.47	0.01
ENSG00000225230	AC008937	0.00	-11.02	0.01
ENSG00000124789	NUP153	0.80	-0.31	0.01
ENSG00000133116	KL	0.14	-2.83	0.01
ENSG00000174799	CEP135	0.60	-0.74	0.01
ENSG00000148341	SH3GLB2	0.75	-0.41	0.01
ENSG00000147471	PLPBP	0.78	-0.35	0.01
ENSG00000144647	POMGNT2	0.75	-0.41	0.01
ENSG00000284879	AC133644	0.56	-0.84	0.01
ENSG00000143178	TBX19	0.57	-0.80	0.01
ENSG00000180035	ZNF48	0.75	-0.42	0.01
ENSG00000143970	ASXL2	0.77	-0.37	0.01
ENSG00000136213	CHST12	0.78	-0.35	0.01
ENSG00000269968	AC006064	0.00	-8.47	0.01
ENSG00000188807	TMEM201	0.72	-0.47	0.01
ENSG00000275322	AC103746	0.38	-1.39	0.01
ENSG00000100167	SEPTIN3	0.05	-4.19	0.01
ENSG00000112530	PACRG	0.10	-3.32	0.01
ENSG00000271880	AGAP11	0.09	-3.51	0.01
ENSG00000213859	KCTD11	0.76	-0.40	0.01
ENSG00000187840	EIF4EBP1	0.84	-0.24	0.01
ENSG00000198055	GRK6	0.81	-0.31	0.01
ENSG00000128602	SMO	0.84	-0.26	0.01
ENSG00000181754	AMIGO1	0.37	-1.42	0.01
ENSG00000214114	MYCBP	0.78	-0.37	0.01

ENSG00000164828	SUN1	0.85	-0.24	0.01
ENSG00000130844	ZNF331	0.34	-1.56	0.01
ENSG00000114268	PFKFB4	0.75	-0.41	0.01
ENSG00000129158	SERGEF	0.70	-0.52	0.01
ENSG00000011021	CLCN6	0.64	-0.65	0.01
ENSG00000187726	DNAJB13	0.45	-1.14	0.01
ENSG00000249859	PVT1	0.72	-0.48	0.01
ENSG00000159363	ATP13A2	0.77	-0.39	0.01
ENSG00000164125	GASK1B	0.80	-0.32	0.01
ENSG00000236088	COX10-AS1	0.40	-1.34	0.01
ENSG00000082781	ITGB5	0.84	-0.25	0.01
ENSG00000182858	ALG12	0.78	-0.36	0.01
ENSG00000107890	ANKRD26	0.63	-0.67	0.01
ENSG00000005108	THSD7A	0.35	-1.53	0.01
ENSG00000170759	KIF5B	0.81	-0.31	0.01
ENSG00000214940	NPIPA8	0.65	-0.63	0.01
ENSG00000149292	TTC12	0.63	-0.66	0.01
ENSG00000182272	B4GALNT4	0.52	-0.95	0.01
ENSG00000171155	C1GALT1C1	0.80	-0.33	0.01
ENSG00000024526	DEPDC1	0.66	-0.59	0.01
ENSG00000147535	PLPP5	0.70	-0.51	0.01
ENSG00000110318	CEP126	0.68	-0.55	0.01
ENSG00000164402	SEPTIN8	0.80	-0.32	0.01
ENSG00000143457	GOLPH3L	0.80	-0.32	0.01
ENSG00000100697	DICER1	0.73	-0.44	0.01
ENSG00000184005	ST6GALNAC3	0.45	-1.15	0.01
ENSG00000185046	ANKS1B	0.39	-1.36	0.01
ENSG00000273038	AL365203	0.56	-0.82	0.01
ENSG00000100505	TRIM9	0.61	-0.70	0.01
ENSG00000213390	ARHGAP19	0.72	-0.47	0.01
ENSG00000164989	CCDC171	0.41	-1.29	0.01
ENSG00000186352	ANKRD37	0.50	-0.99	0.01
ENSG00000205309	NT5M	0.26	-1.92	0.01
ENSG00000106771	TMEM245	0.84	-0.26	0.01
ENSG00000107281	NPDC1	0.67	-0.58	0.01
ENSG00000165516	KLHDC2	0.70	-0.52	0.01
ENSG00000219545	UMAD1	0.68	-0.57	0.01
ENSG00000107902	LHPP	0.67	-0.58	0.01
ENSG00000125968	ID1	0.80	-0.33	0.01
ENSG00000249363	AC011411	0.00	-11.03	0.01
ENSG00000090776	EFNB1	0.64	-0.64	0.01
ENSG00000245662	LINC02211	0.20	-2.32	0.01
ENSG00000130856	ZNF236	0.71	-0.49	0.01
ENSG00000129007	CALML4	0.54	-0.90	0.01
ENSG00000196275	GTF2IRD2	0.63	-0.66	0.01
ENSG00000141668	CBLN2	0.09	-3.54	0.01
ENSG00000111450	STX2	0.79	-0.35	0.01
ENSG00000122420	PTGFR	0.63	-0.67	0.01
ENSG00000012983	MAP4K5	0.79	-0.34	0.01
ENSG00000143924	EML4	0.82	-0.28	0.01
ENSG00000152380	FAM151B	0.33	-1.60	0.01
ENSG00000176619	LMNB2	0.85	-0.24	0.01
ENSG00000128731	HERC2	0.83	-0.28	0.01
ENSG00000173064	HECTD4	0.82	-0.29	0.01
ENSG00000121310	ECHDC2	0.79	-0.33	0.01
ENSG00000161920	MED11	0.66	-0.59	0.01
ENSG00000125629	INSIG2	0.66	-0.61	0.01
ENSG00000137161	CNPY3	0.82	-0.29	0.01
ENSG00000197332	AC008543	0.02	-5.41	0.01
ENSG00000267980	AC007292	0.14	-2.85	0.01
ENSG00000147536	GINS4	0.82	-0.29	0.01

ENSG00000102144	PGK1	0.84	-0.25	0.01
ENSG00000170004	CHD3	0.84	-0.24	0.01
ENSG00000168758	SEMA4C	0.69	-0.53	0.01
ENSG00000125124	BBS2	0.79	-0.34	0.01
ENSG00000109066	TMEM104	0.84	-0.26	0.01
ENSG00000177853	ZNF518A	0.64	-0.63	0.01
ENSG00000166507	NDST2	0.67	-0.59	0.01
ENSG00000138434	ITPRID2	0.78	-0.35	0.01
ENSG00000118689	FOXO3	0.80	-0.32	0.01
ENSG00000157322	CLEC18A	0.21	-2.27	0.01
ENSG00000236008	LINC01814	0.30	-1.74	0.01
ENSG00000109606	DHX15	0.84	-0.25	0.01
ENSG00000153107	ANAPC1	0.84	-0.24	0.01
ENSG00000119574	ZBTB45	0.74	-0.44	0.01
ENSG00000184838	PRR16	0.37	-1.43	0.01
ENSG00000103197	TSC2	0.83	-0.27	0.01
ENSG00000274419	TBC1D3D	0.67	-0.58	0.01
ENSG00000050438	SLC4A8	0.29	-1.78	0.01
ENSG00000137496	IL18BP	0.64	-0.65	0.01
ENSG00000171659	GPR34	0.00	-9.90	0.01
ENSG00000157456	CCNB2	0.80	-0.33	0.01
ENSG00000168038	ULK4	0.66	-0.61	0.01
ENSG00000180530	NRIP1	0.82	-0.29	0.01
ENSG00000085224	ATRX	0.65	-0.61	0.01
ENSG00000261578	AP003119	0.40	-1.31	0.01
ENSG00000116273	PHF13	0.82	-0.29	0.01
ENSG00000213599	SLX1A-SULT1A3	0.67	-0.58	0.01
ENSG00000135324	MRAP2	0.42	-1.24	0.01
ENSG00000238083	LRRC37A2	0.70	-0.52	0.01
ENSG00000270087	AC010997	0.00	-11.42	0.01
ENSG00000158966	CACHD1	0.66	-0.59	0.01
ENSG00000083720	OXCT1	0.77	-0.37	0.01
ENSG00000136828	RALGPS1	0.28	-1.84	0.01
ENSG00000152520	PAN3	0.77	-0.37	0.01
ENSG00000136051	WASHC4	0.73	-0.45	0.01
ENSG00000260804	LINC01963	0.62	-0.68	0.01
ENSG00000164105	SAP30	0.69	-0.54	0.01
ENSG00000100583	SAMD15	0.44	-1.19	0.01
ENSG00000090975	PITPNM2	0.77	-0.37	0.01
ENSG00000148200	NR6A1	0.48	-1.05	0.01
ENSG00000139324	TMTC3	0.76	-0.40	0.01
ENSG00000157833	GAREM2	0.65	-0.63	0.01
ENSG00000172057	ORMDL3	0.83	-0.27	0.01
ENSG00000138376	BARD1	0.64	-0.64	0.01
ENSG00000113569	NUP155	0.82	-0.29	0.01
ENSG00000120616	EPC1	0.70	-0.52	0.01
ENSG00000155229	MMS19	0.84	-0.25	0.01
ENSG00000137507	LRRC32	0.07	-3.89	0.01
ENSG00000090238	YPEL3	0.72	-0.48	0.01
ENSG00000197818	SLC9A8	0.78	-0.35	0.01
ENSG00000225420	AC104134	0.00	-10.07	0.01
ENSG00000286056	AC008055	0.00	-9.46	0.01
ENSG00000163736	PPBP	0.00	-10.27	0.01
ENSG00000127329	PTPRB	0.76	-0.39	0.01
ENSG00000168743	NPNT	0.22	-2.16	0.01
ENSG00000122783	CYREN	0.81	-0.30	0.01
ENSG00000152785	BMP3	0.00	-8.05	0.01
ENSG00000231674	LINC00410	0.00	-9.64	0.01
ENSG00000179841	AKAP5	0.20	-2.33	0.01
ENSG00000106927	AMBP	0.54	-0.89	0.01
ENSG00000197705	KLHL14	0.00	-8.40	0.01

ENSG00000238105	GOLGA2P5	0.38	-1.38	0.01
ENSG00000180573	H2AC6	0.54	-0.90	0.01
ENSG00000099326	MZF1	0.78	-0.36	0.01
ENSG00000167775	CD320	0.81	-0.30	0.01
ENSG00000105325	FZR1	0.73	-0.46	0.01
ENSG00000148730	EIF4EBP2	0.84	-0.26	0.01
ENSG00000164118	CEP44	0.64	-0.65	0.01
ENSG00000148824	MTG1	0.78	-0.35	0.01
ENSG00000151287	TEX30	0.71	-0.50	0.01
ENSG00000111305	GSG1	0.00	-9.14	0.01
ENSG00000164953	TMEM67	0.68	-0.55	0.01
ENSG00000255389	Z97989	0.22	-2.19	0.01
ENSG00000139160	ETFBKMT	0.33	-1.62	0.01
ENSG00000101222	SPEF1	0.06	-4.08	0.01
ENSG00000277531	PNMA8C	0.10	-3.31	0.01
ENSG00000072135	PTPN18	0.71	-0.49	0.01
ENSG00000103510	KAT8	0.80	-0.32	0.01
ENSG00000008277	ADAM22	0.60	-0.74	0.01
ENSG00000164151	ICE1	0.80	-0.32	0.01
ENSG00000182557	SPNS3	0.42	-1.26	0.01
ENSG00000118997	DNAH7	0.40	-1.33	0.01
ENSG00000164961	WASHC5	0.78	-0.36	0.01
ENSG00000163848	ZNF148	0.78	-0.36	0.01
ENSG00000165591	FAAH2	0.06	-4.07	0.01
ENSG00000005882	PDK2	0.76	-0.39	0.01
ENSG00000132004	FBXW9	0.68	-0.56	0.01
ENSG00000095777	MYO3A	0.43	-1.22	0.01
ENSG00000265763	ZNF488	0.15	-2.71	0.01
ENSG00000181788	SIAH2	0.79	-0.35	0.01
ENSG00000147533	GOLGA7	0.75	-0.41	0.01
ENSG00000272333	KMT2B	0.83	-0.27	0.01
ENSG00000161847	RAVER1	0.83	-0.26	0.01
ENSG00000168615	ADAM9	0.84	-0.25	0.01
ENSG00000229743	LINC01159	0.34	-1.55	0.01
ENSG00000115652	UXS1	0.73	-0.46	0.01
ENSG00000110514	MADD	0.83	-0.27	0.01
ENSG00000183793	NPIPA5	0.52	-0.95	0.01
ENSG00000166225	FRS2	0.50	-1.00	0.01
ENSG00000104880	ARHGEF18	0.82	-0.29	0.01
ENSG00000180104	EXOC3	0.84	-0.25	0.01
ENSG00000147485	PXDNL	0.18	-2.48	0.01
ENSG00000132967	HMGB1P5	0.78	-0.35	0.01
ENSG00000135916	ITM2C	0.83	-0.27	0.01
ENSG00000197329	PELI1	0.73	-0.45	0.01
ENSG00000064651	SLC12A2	0.79	-0.35	0.01
ENSG00000254844	AP000757	0.05	-4.30	0.01
ENSG00000177508	IRX3	0.22	-2.17	0.01
ENSG00000276718	AC005840	0.08	-3.69	0.01
ENSG00000169855	ROBO1	0.78	-0.35	0.01
ENSG00000141499	WRAP53	0.76	-0.39	0.01
ENSG00000287896	AC073548	0.00	-9.49	0.01
ENSG00000198513	ATL1	0.63	-0.66	0.01
ENSG00000128159	TUBGCP6	0.76	-0.39	0.01
ENSG00000102554	KLF5	0.62	-0.70	0.01
ENSG00000128581	IFT22	0.76	-0.39	0.01
ENSG00000176697	BDNF	0.72	-0.47	0.01
ENSG00000174586	ZNF497	0.00	-11.45	0.01
ENSG00000196396	PTPN1	0.86	-0.22	0.01
ENSG00000169660	HEXD	0.74	-0.44	0.01
ENSG00000176894	PXMP2	0.74	-0.43	0.01
ENSG00000204842	ATXN2	0.78	-0.36	0.01

ENSG00000125089	SH3TC1	0.70	-0.52	0.01
ENSG00000107625	DDX50	0.74	-0.44	0.01
ENSG00000078967	UBE2D4	0.64	-0.65	0.01
ENSG00000288597	AC234782	0.02	-5.38	0.01
ENSG00000181722	ZBTB20	0.46	-1.13	0.01
ENSG00000153936	HS2ST1	0.83	-0.27	0.01
ENSG00000145675	PIK3R1	0.80	-0.31	0.01
ENSG00000138398	PPIG	0.78	-0.37	0.01
ENSG00000120075	HOXB5	0.68	-0.55	0.01
ENSG00000108840	HDAC5	0.77	-0.37	0.01
ENSG00000150712	MTMR12	0.80	-0.32	0.01
ENSG00000113763	UNC5A	0.08	-3.56	0.01
ENSG00000160051	IQCC	0.62	-0.68	0.01
ENSG00000068489	PRR11	0.86	-0.22	0.01
ENSG00000083642	PDS5B	0.70	-0.52	0.01
ENSG00000146267	FAXC	0.39	-1.35	0.01
ENSG00000205041	AC118344	0.37	-1.45	0.01
ENSG00000224597	SVIL-AS1	0.61	-0.71	0.01
ENSG00000182240	BACE2	0.76	-0.40	0.01
ENSG00000104517	UBR5	0.85	-0.24	0.01
ENSG00000183426	NPIPA1	0.69	-0.55	0.01
ENSG00000132016	C19orf57	0.48	-1.07	0.01
ENSG00000132522	GPS2	0.81	-0.30	0.01
ENSG00000138658	ZGRF1	0.55	-0.85	0.01
ENSG00000168487	BMP1	0.81	-0.30	0.01
ENSG00000254739	AC137894	0.00	-11.48	0.01
ENSG00000187837	H1-2	0.69	-0.53	0.01
ENSG00000154874	CCDC144B	0.02	-5.79	0.01
ENSG00000282936	AC004706	0.62	-0.70	0.01
ENSG00000273046	AC012531	0.00	-11.28	0.01
ENSG00000138161	CUZD1	0.04	-4.75	0.01
ENSG00000170515	PA2G4	0.85	-0.23	0.01
ENSG00000102471	NDFIP2	0.75	-0.42	0.01
ENSG00000109787	KLF3	0.83	-0.27	0.01
ENSG00000173890	GPR160	0.54	-0.90	0.01
ENSG00000173531	MST1	0.46	-1.11	0.01
ENSG00000105227	PRX	0.61	-0.72	0.01
ENSG00000145982	FARS2	0.76	-0.39	0.01
ENSG00000213625	LEPROT	0.83	-0.26	0.01
ENSG00000100129	EIF3L	0.84	-0.25	0.01
ENSG00000205129	C4orf47	0.37	-1.42	0.01
ENSG00000028528	SNX1	0.83	-0.26	0.01
ENSG00000135829	DHX9	0.86	-0.22	0.01
ENSG00000204745	AC083899	0.64	-0.64	0.01
ENSG00000161328	LRRC56	0.31	-1.69	0.01
ENSG00000213977	TAX1BP3	0.85	-0.23	0.01
ENSG00000186409	CCDC30	0.48	-1.06	0.01
ENSG00000013441	CLK1	0.78	-0.36	0.01
ENSG00000157212	PAXIP1	0.80	-0.31	0.01
ENSG00000139687	RB1	0.74	-0.43	0.01
ENSG00000134873	CLDN10	0.00	-9.95	0.01
ENSG00000106346	USP42	0.76	-0.39	0.01
ENSG00000286075	AC009412	0.68	-0.55	0.01
ENSG00000106003	LFNG	0.84	-0.25	0.01
ENSG00000198707	CEP290	0.70	-0.51	0.01
ENSG00000188428	BLOC1S5	0.65	-0.61	0.01
ENSG00000102893	PHKB	0.78	-0.36	0.01
ENSG00000119969	HELLS	0.62	-0.69	0.01
ENSG00000180155	LYNX1	0.54	-0.88	0.01
ENSG00000014824	SLC30A9	0.84	-0.26	0.01
ENSG00000162735	PEX19	0.69	-0.54	0.01

ENSG00000138767	CNOT6L	0.77	-0.38	0.01
ENSG00000038002	AGA	0.81	-0.31	0.01
ENSG00000170242	USP47	0.80	-0.32	0.01
ENSG00000164093	PITX2	0.65	-0.62	0.01
ENSG00000173757	STAT5B	0.84	-0.26	0.01
ENSG00000241790	ENO1P4	0.63	-0.67	0.01
ENSG00000144746	ARL6IP5	0.83	-0.26	0.01
ENSG00000280543	ASAP1-IT2	0.00	-9.63	0.01
ENSG00000262823	AC127521	0.00	-10.47	0.01
ENSG00000286724	AL732509	0.33	-1.60	0.01
ENSG00000272273	IER3-AS1	0.00	-11.49	0.01
ENSG00000179094	PER1	0.74	-0.43	0.01
ENSG00000176014	TUBB6	0.75	-0.42	0.01
ENSG00000156466	GDF6	0.25	-1.97	0.01
ENSG00000167774	AC010323	0.60	-0.73	0.01
ENSG00000206530	CFAP44	0.62	-0.69	0.01
ENSG00000254469	AP002495	0.37	-1.43	0.01
ENSG00000134900	TPP2	0.82	-0.28	0.01
ENSG00000168010	ATG16L2	0.61	-0.71	0.01
ENSG00000272870	SAP30-DT	0.32	-1.66	0.01
ENSG00000140538	NTRK3	0.27	-1.91	0.01
ENSG00000108852	MPP2	0.68	-0.55	0.01
ENSG00000185432	METTL7A	0.47	-1.09	0.01
ENSG00000251685	UGT2B27P	0.00	-9.84	0.01
ENSG00000197785	ATAD3A	0.85	-0.24	0.01
ENSG00000156050	FAM161B	0.62	-0.68	0.01
ENSG00000095564	BTAF1	0.80	-0.32	0.01
ENSG00000101191	DIDO1	0.85	-0.23	0.01
ENSG00000258749	AL110504	0.44	-1.19	0.01
ENSG00000134138	MEIS2	0.63	-0.67	0.01
ENSG00000197168	NEK5	0.32	-1.67	0.01
ENSG00000106479	ZNF862	0.75	-0.42	0.01
ENSG00000147548	NSD3	0.73	-0.45	0.01
ENSG00000141294	LRRC46	0.49	-1.04	0.01
ENSG00000111271	ACAD10	0.78	-0.35	0.01
ENSG00000198160	MIER1	0.75	-0.41	0.01
ENSG00000164684	ZNF704	0.68	-0.56	0.01
ENSG00000039319	ZFYVE16	0.78	-0.36	0.01
ENSG00000161671	EMC10	0.83	-0.27	0.01
ENSG00000187790	FANCM	0.54	-0.88	0.01
ENSG00000233381	AK4P3	0.38	-1.41	0.01
ENSG00000103494	RPGRIP1L	0.77	-0.37	0.01
ENSG00000113318	MSH3	0.78	-0.36	0.01
ENSG00000071205	ARHGAP10	0.81	-0.31	0.01
ENSG00000187676	B3GLCT	0.73	-0.46	0.01
ENSG00000128789	PSMG2	0.84	-0.26	0.01
ENSG00000084676	NCOA1	0.73	-0.46	0.01
ENSG00000254852	NPIPA2	0.22	-2.20	0.01
ENSG00000145916	RMND5B	0.78	-0.35	0.01
ENSG00000156876	SASS6	0.53	-0.92	0.01
ENSG00000172878	METAP1D	0.74	-0.43	0.01
ENSG00000100307	CBX7	0.64	-0.65	0.01
ENSG00000039139	DNAH5	0.68	-0.56	0.01
ENSG00000228839	PIK3IP1-DT	0.00	-11.32	0.01
ENSG00000141699	RETREG3	0.84	-0.25	0.01
ENSG00000171345	KRT19	0.62	-0.70	0.01
ENSG00000259345	AC013652	0.33	-1.60	0.01
ENSG00000163527	STT3B	0.86	-0.22	0.01
ENSG00000031691	CENPQ	0.66	-0.61	0.01
ENSG00000150995	ITPR1	0.69	-0.53	0.01
ENSG00000170264	FAM161A	0.60	-0.74	0.01

ENSG00000134824	FADS2	0.70	-0.52	0.01
ENSG00000130714	POMT1	0.80	-0.33	0.01
ENSG00000078142	PIK3C3	0.78	-0.35	0.01
ENSG00000196912	ANKRD36B	0.49	-1.01	0.01
ENSG00000196632	WNK3	0.56	-0.84	0.01
ENSG00000134852	CLOCK	0.79	-0.35	0.01
ENSG00000123810	B9D2	0.66	-0.59	0.01
ENSG00000072840	EVC	0.86	-0.22	0.01
ENSG00000134222	PSRC1	0.72	-0.47	0.01
ENSG00000108588	CCDC47	0.85	-0.24	0.01
ENSG00000213606	AKR1B10P1	0.05	-4.39	0.01
ENSG00000232040	ZBED9	0.42	-1.26	0.01
ENSG00000272944	AC079834	0.28	-1.81	0.01
ENSG00000136146	MED4	0.79	-0.34	0.01
ENSG00000138074	SLC5A6	0.83	-0.27	0.01
ENSG00000188051	TMEM221	0.00	-9.31	0.01
ENSG00000188452	CERKL	0.39	-1.36	0.01
ENSG00000226887	ERVMER34-1	0.65	-0.63	0.01
ENSG00000213104	NPM1P46	0.00	-10.85	0.01
ENSG00000187240	DYNC2H1	0.61	-0.71	0.01
ENSG00000276805	AL133216	0.53	-0.93	0.01
ENSG00000109610	SOD3	0.54	-0.88	0.01
ENSG00000154146	NRGN	0.66	-0.59	0.01
ENSG00000125246	CLYBL	0.63	-0.67	0.01
ENSG00000106443	PHF14	0.72	-0.48	0.01
ENSG00000162736	NCSTN	0.85	-0.23	0.01
ENSG00000100982	PCIF1	0.83	-0.28	0.02
ENSG00000214753	HNRNPUL2	0.86	-0.21	0.02
ENSG00000122970	IFT81	0.71	-0.49	0.02
ENSG00000077097	TOP2B	0.81	-0.30	0.02
ENSG00000198792	TMEM184B	0.83	-0.27	0.02
ENSG00000109881	CCDC34	0.75	-0.42	0.02
ENSG00000114021	NIT2	0.85	-0.23	0.02
ENSG00000110429	FBXO3	0.75	-0.41	0.02
ENSG00000040341	STAU2	0.81	-0.31	0.02
ENSG00000167646	DNAAF3	0.29	-1.81	0.02
ENSG00000135956	TMEM127	0.85	-0.24	0.02
ENSG00000250251	PKD1P6	0.70	-0.51	0.02
ENSG00000073910	FRY	0.48	-1.06	0.02
ENSG00000274565	AC080038	0.58	-0.78	0.02
ENSG00000138081	FBXO11	0.80	-0.32	0.02
ENSG00000178966	RMI1	0.70	-0.52	0.02
ENSG00000152061	RABGAP1L	0.66	-0.61	0.02
ENSG00000108947	EFNB3	0.33	-1.60	0.02
ENSG00000100478	AP4S1	0.65	-0.62	0.02
ENSG00000160294	MCM3AP	0.83	-0.26	0.02
ENSG00000241973	PI4KA	0.85	-0.23	0.02
ENSG00000181544	FANCB	0.62	-0.70	0.02
ENSG00000125872	LRRN4	0.70	-0.51	0.02
ENSG00000271380	AL451085	0.28	-1.84	0.02
ENSG00000144043	TEX261	0.85	-0.24	0.02
ENSG00000211445	GPX3	0.66	-0.60	0.02
ENSG00000241563	CORT	0.02	-5.69	0.02
ENSG00000175220	ARHGAP1	0.85	-0.23	0.02
ENSG00000136153	LMO7	0.72	-0.48	0.02
ENSG00000203706	SERTAD4-AS1	0.63	-0.66	0.02
ENSG00000100320	RBFOX2	0.86	-0.22	0.02
ENSG00000260233	ZNRD2-AS1	0.45	-1.15	0.02
ENSG00000105879	CBLL1	0.57	-0.81	0.02
ENSG00000171241	SHCBP1	0.83	-0.26	0.02
ENSG00000225205	AC078883	0.38	-1.41	0.02

ENSG00000144567	RETREG2	0.76	-0.40	0.02
ENSG00000188747	NOXA1	0.00	-9.73	0.02
ENSG00000114023	FAM162A	0.82	-0.28	0.02
ENSG00000111731	C2CD5	0.76	-0.39	0.02
ENSG00000066027	PPP2R5A	0.80	-0.32	0.02
ENSG00000060749	OSER1	0.68	-0.56	0.02
ENSG00000148737	TCF7L2	0.65	-0.62	0.02
ENSG00000177311	ZBTB38	0.22	-2.16	0.02
ENSG00000163040	CCDC74A	0.74	-0.44	0.02
ENSG00000179029	TMEM107	0.66	-0.60	0.02
ENSG00000048991	R3HDM1	0.84	-0.26	0.02
ENSG00000158545	ZC3H18	0.82	-0.29	0.02
ENSG00000145850	TIMD4	0.53	-0.92	0.02
ENSG00000154678	PDE1C	0.02	-5.44	0.02
ENSG00000118690	ARMC2	0.46	-1.13	0.02
ENSG00000124155	PIGT	0.76	-0.39	0.02
ENSG00000164362	TERT	0.56	-0.83	0.02
ENSG00000135365	PHF21A	0.82	-0.28	0.02
ENSG00000183258	DDX41	0.85	-0.24	0.02
ENSG00000138792	ENPEP	0.59	-0.75	0.02
ENSG00000254389	RHPN1-AS1	0.47	-1.09	0.02
ENSG00000124641	MED20	0.68	-0.56	0.02
ENSG00000269148	AC092301	0.00	-10.90	0.02
ENSG00000287402	AL158167	0.00	-9.91	0.02
ENSG00000198270	TMEM116	0.67	-0.58	0.02
ENSG00000107829	FBXW4	0.82	-0.29	0.02
ENSG00000117298	ECE1	0.87	-0.20	0.02
ENSG00000169562	GJB1	0.00	-8.45	0.02
ENSG00000171757	LRRC34	0.00	-8.27	0.02
ENSG00000215883	CYB5RL	0.64	-0.63	0.02
ENSG00000255874	PRECSIT	0.77	-0.37	0.02
ENSG00000204576	PRR3	0.77	-0.37	0.02
ENSG00000273311	DGCR11	0.64	-0.63	0.02
ENSG00000102904	TSNAXIP1	0.38	-1.39	0.02
ENSG00000076248	UNG	0.84	-0.26	0.02
ENSG00000169302	STK32A	0.46	-1.13	0.02
ENSG00000261794	GOLGA8H	0.14	-2.84	0.02
ENSG00000177640	CASC2	0.45	-1.14	0.02
ENSG00000137337	MDC1	0.81	-0.31	0.02
ENSG00000169446	MMGT1	0.83	-0.28	0.02
ENSG00000132436	FIGNL1	0.64	-0.65	0.02
ENSG00000196693	ZNF33B	0.49	-1.02	0.02
ENSG00000117477	CCDC181	0.60	-0.75	0.02
ENSG00000283378	CNTNAP3C	0.33	-1.62	0.02
ENSG00000146950	SHROOM2	0.45	-1.16	0.02
ENSG00000188176	SMTNL2	0.00	-9.12	0.02
ENSG00000089335	ZNF302	0.62	-0.68	0.02
ENSG00000071537	SEL1L	0.86	-0.22	0.02
ENSG00000144485	HES6	0.43	-1.21	0.02
ENSG00000086200	IPO11	0.81	-0.30	0.02
ENSG00000155629	PIK3AP1	0.74	-0.43	0.02
ENSG00000141905	NFIC	0.77	-0.37	0.02
ENSG00000231426	FILNC1	0.00	-9.55	0.02
ENSG00000102038	SMARCA1	0.84	-0.25	0.02
ENSG00000138769	CDKL2	0.46	-1.12	0.02
ENSG00000139874	SSTR1	0.50	-1.01	0.02
ENSG00000065135	GNAI3	0.83	-0.26	0.02
ENSG00000231584	FAHD2CP	0.32	-1.63	0.02
ENSG00000137822	TUBGCP4	0.79	-0.34	0.02
ENSG00000102977	ACD	0.78	-0.36	0.02
ENSG00000250767	AC139783	0.06	-4.08	0.02

ENSG00000250091	DNAH10OS	0.32	-1.64	0.02
ENSG00000173013	CCDC96	0.72	-0.48	0.02
ENSG00000183346	CABCOCO1	0.31	-1.67	0.02
ENSG00000271167	LINC01109	0.09	-3.43	0.02
ENSG00000070366	SMG6	0.83	-0.27	0.02
ENSG00000184500	PROS1	0.71	-0.49	0.02
ENSG00000230074	AL162231	0.65	-0.62	0.02
ENSG00000144228	SPOPL	0.77	-0.38	0.02
ENSG00000141867	BRD4	0.85	-0.23	0.02
ENSG00000285644	AC108448	0.43	-1.22	0.02
ENSG00000089820	ARHGAP4	0.11	-3.14	0.02
ENSG00000176225	RTTN	0.76	-0.39	0.02
ENSG00000288063	AC099050	0.00	-9.77	0.02
ENSG00000168589	DYNLRB2	0.00	-9.86	0.02
ENSG00000013573	DDX11	0.83	-0.26	0.02
ENSG00000177169	ULK1	0.80	-0.32	0.02
ENSG00000162694	EXTL2	0.70	-0.52	0.02
ENSG00000166183	ASPG	0.00	-8.00	0.02
ENSG00000005302	MSL3	0.81	-0.31	0.02
ENSG00000131507	NDFIP1	0.85	-0.23	0.02
ENSG00000144395	CCDC150	0.56	-0.84	0.02
ENSG00000040608	RTN4R	0.07	-3.81	0.02
ENSG00000196290	NIF3L1	0.79	-0.34	0.02
ENSG00000109458	GAB1	0.72	-0.48	0.02
ENSG00000149657	LSM14B	0.82	-0.28	0.02
ENSG00000267811	AP001160	0.12	-3.12	0.02
ENSG00000127663	KDM4B	0.83	-0.26	0.02
ENSG00000274180	NATD1	0.77	-0.39	0.02
ENSG00000261371	PECAM1	0.32	-1.66	0.02
ENSG00000100442	FKBP3	0.81	-0.31	0.02
ENSG00000269973	AC010969	0.40	-1.32	0.02
ENSG00000230006	ANKRD36BP2	0.32	-1.66	0.02
ENSG00000198176	TFDP1	0.82	-0.29	0.02
ENSG00000177106	EPS8L2	0.85	-0.24	0.02
ENSG00000186501	TMEM222	0.70	-0.52	0.02
ENSG00000183814	LIN9	0.73	-0.45	0.02
ENSG00000213347	MXD3	0.79	-0.33	0.02
ENSG00000099377	HSD3B7	0.75	-0.42	0.02
ENSG00000184903	IMMP2L	0.63	-0.66	0.02
ENSG00000032742	IFT88	0.59	-0.77	0.02
ENSG00000235605	AL355472	0.09	-3.41	0.02
ENSG00000273247	AC097376	0.42	-1.25	0.02
ENSG00000136715	SAP130	0.82	-0.28	0.02
ENSG00000152291	TGOLN2	0.87	-0.20	0.02
ENSG00000164136	IL15	0.65	-0.63	0.02
ENSG00000101138	CSTF1	0.78	-0.35	0.02
ENSG00000064961	HMG20B	0.82	-0.28	0.02
ENSG00000257354	AC048341	0.74	-0.43	0.02
ENSG00000196284	SUPT3H	0.70	-0.50	0.02
ENSG00000125534	PPDPF	0.85	-0.24	0.02
ENSG00000172239	PAIP1	0.82	-0.28	0.02
ENSG00000129219	PLD2	0.83	-0.27	0.02
ENSG00000180739	S1PR5	0.56	-0.84	0.02
ENSG00000064763	FAR2	0.80	-0.32	0.02
ENSG00000279673	AC092919	0.40	-1.32	0.02
ENSG00000107984	DKK1	0.58	-0.79	0.02
ENSG00000100425	BRD1	0.80	-0.32	0.02
ENSG00000229559	AL353597	0.00	-10.47	0.02
ENSG00000275120	AC048382	0.00	-8.71	0.02
ENSG00000197535	MYO5A	0.71	-0.49	0.02
ENSG00000130396	AFDN	0.84	-0.26	0.02

ENSG00000173894	CBX2	0.54	-0.89	0.02
ENSG00000142166	IFNAR1	0.72	-0.48	0.02
ENSG00000162512	SDC3	0.83	-0.27	0.02
ENSG00000124743	KLHL31	0.26	-1.92	0.02
ENSG00000146828	SLC12A9	0.83	-0.26	0.02
ENSG00000243716	NPIPB5	0.78	-0.36	0.02
ENSG00000183010	PYCR1	0.71	-0.49	0.02
ENSG00000274092	AC106739	0.37	-1.45	0.02
ENSG00000110696	C11orf58	0.87	-0.20	0.02
ENSG00000115183	TANC1	0.81	-0.30	0.02
ENSG00000130939	UBE4B	0.84	-0.26	0.02
ENSG00000094804	CDC6	0.83	-0.27	0.02
ENSG00000118432	CNR1	0.28	-1.83	0.02
ENSG00000127616	SMARCA4	0.86	-0.21	0.02
ENSG00000172014	ANKRD20A4P	0.03	-5.03	0.02
ENSG00000163002	NUP35	0.74	-0.43	0.02
ENSG00000132563	REEP2	0.57	-0.81	0.02
ENSG00000064419	TNPO3	0.87	-0.20	0.02
ENSG00000164896	FASTK	0.82	-0.28	0.02
ENSG00000111907	TPD52L1	0.80	-0.33	0.02
ENSG00000026297	RNASET2	0.74	-0.44	0.02
ENSG00000167595	PROSER3	0.70	-0.52	0.02
ENSG00000285676	AL158212	0.00	-10.11	0.02
ENSG00000179941	BBS10	0.74	-0.43	0.02
ENSG00000264204	AGAP7P	0.26	-1.96	0.02
ENSG00000105248	YJU2	0.76	-0.39	0.02
ENSG00000102796	DHRS12	0.64	-0.65	0.02
ENSG00000183833	CFAP91	0.55	-0.86	0.02
ENSG00000276168	RN7SL1	0.10	-3.29	0.02
ENSG00000138303	ASCC1	0.81	-0.30	0.02
ENSG00000129048	ACKR4	0.50	-0.99	0.02
ENSG00000115641	FHL2	0.88	-0.19	0.02
ENSG00000162032	SPSB3	0.73	-0.44	0.02
ENSG00000105996	HOXA2	0.51	-0.97	0.02
ENSG00000153234	NR4A2	0.44	-1.19	0.02
ENSG00000141646	SMAD4	0.75	-0.42	0.02
ENSG00000178177	LCORL	0.63	-0.66	0.02
ENSG00000248333	CDK11B	0.86	-0.22	0.02
ENSG00000122877	EGR2	0.26	-1.97	0.02
ENSG00000112167	SAYSD1	0.73	-0.45	0.02
ENSG00000105948	TTC26	0.62	-0.68	0.02
ENSG00000076944	STXBP2	0.84	-0.25	0.02
ENSG00000135315	CEP162	0.61	-0.72	0.02
ENSG00000267414	SETBP1-DT	0.14	-2.83	0.02
ENSG00000186298	PPP1CC	0.80	-0.33	0.02
ENSG00000288398	AL109627	0.65	-0.62	0.02
ENSG00000104824	HNRNPL	0.88	-0.19	0.02
ENSG00000170689	HOXB9	0.83	-0.27	0.02
ENSG00000100784	RPS6KA5	0.68	-0.55	0.02
ENSG00000004534	RBM6	0.79	-0.33	0.02
ENSG00000144857	BOC	0.51	-0.97	0.02
ENSG00000276966	H4C5	0.16	-2.63	0.02
ENSG00000178860	MSC	0.88	-0.19	0.02
ENSG00000140284	SLC27A2	0.30	-1.75	0.02
ENSG00000204682	MIR1915HG	0.79	-0.34	0.02
ENSG00000166813	KIF7	0.71	-0.49	0.02
ENSG00000106686	SPATA6L	0.52	-0.93	0.02
ENSG00000286553	AL357075	0.08	-3.68	0.02
ENSG00000129946	SHC2	0.12	-3.05	0.02
ENSG00000100503	NIN	0.83	-0.27	0.02
ENSG00000105519	CAPS	0.62	-0.68	0.02

ENSG00000175130	MARCKSL1	0.87	-0.21	0.02
ENSG00000144589	STK11IP	0.82	-0.29	0.02
ENSG00000280055	TMEM75	0.04	-4.74	0.02
ENSG00000204138	PHACTR4	0.71	-0.49	0.02
ENSG00000147601	TERF1	0.83	-0.27	0.02
ENSG00000174353	STAG3L3	0.82	-0.29	0.02
ENSG00000170379	TCAF2	0.51	-0.98	0.02
ENSG00000112293	GPLD1	0.41	-1.28	0.02
ENSG00000008952	SEC62	0.83	-0.27	0.02
ENSG00000158292	GPR153	0.64	-0.64	0.02
ENSG00000011114	BTBD7	0.78	-0.37	0.02
ENSG00000018699	TTC27	0.79	-0.34	0.02
ENSG00000151849	CENPJ	0.71	-0.50	0.02
ENSG00000021762	OSBPL5	0.73	-0.45	0.02
ENSG00000159409	CELF3	0.00	-7.74	0.02
ENSG00000120539	MASTL	0.80	-0.32	0.02
ENSG00000198824	CHAMP1	0.84	-0.25	0.02
ENSG00000205746	AC126755	0.68	-0.55	0.02
ENSG00000089876	DHX32	0.84	-0.24	0.02
ENSG00000160111	CPAMD8	0.25	-1.98	0.02
ENSG00000111879	FAM184A	0.39	-1.35	0.02
ENSG00000204427	ABHD16A	0.79	-0.33	0.02
ENSG00000069018	TRPC7	0.00	-8.43	0.02
ENSG00000254471	AP001284	0.00	-9.23	0.02
ENSG00000180818	HOXC10	0.77	-0.39	0.02
ENSG00000075292	ZNF638	0.78	-0.36	0.02
ENSG00000138336	TET1	0.38	-1.40	0.02
ENSG00000085511	MAP3K4	0.80	-0.33	0.02
ENSG00000274964	AC026356	0.36	-1.49	0.02
ENSG00000077454	LRCH4	0.74	-0.43	0.02
ENSG00000130733	YIPF2	0.82	-0.29	0.02
ENSG00000286805	AL138716	0.31	-1.67	0.02
ENSG00000250021	ARPIN-AP3S2	0.11	-3.18	0.02
ENSG00000036257	CUL3	0.85	-0.24	0.02
ENSG00000172461	FUT9	0.45	-1.16	0.02
ENSG00000237515	SHISA9	0.80	-0.32	0.02
ENSG00000134250	NOTCH2	0.88	-0.19	0.02
ENSG00000166171	DPCD	0.80	-0.32	0.02
ENSG00000077942	FBLN1	0.80	-0.32	0.02
ENSG00000198727	MT-CYB	0.88	-0.19	0.02
ENSG00000108406	DHX40	0.81	-0.31	0.02
ENSG00000048828	FAM120A	0.87	-0.19	0.02
ENSG00000274012	RN7SL2	0.08	-3.69	0.02
ENSG00000154359	LONRF1	0.68	-0.56	0.02
ENSG00000157551	KCNJ15	0.01	-6.94	0.02
ENSG00000163050	COQ8A	0.84	-0.26	0.02
ENSG00000198756	COLGALT2	0.47	-1.09	0.02
ENSG00000168795	ZBTB5	0.80	-0.32	0.02
ENSG00000081386	ZNF510	0.68	-0.56	0.02
ENSG00000151090	THRB	0.53	-0.92	0.02
ENSG00000116984	MTR	0.78	-0.37	0.02
ENSG00000285967	NIPBL-DT	0.69	-0.54	0.02
ENSG00000118816	CCNI	0.87	-0.21	0.02
ENSG00000128513	POT1	0.77	-0.37	0.02
ENSG00000111445	RFC5	0.71	-0.50	0.02
ENSG00000100207	TCF20	0.75	-0.42	0.03
ENSG00000247095	MIR210HG	0.56	-0.84	0.03
ENSG00000226435	ANKRD18DP	0.00	-8.62	0.03
ENSG00000104427	ZC2HC1A	0.76	-0.40	0.03
ENSG00000285601	AC084262	0.00	-8.63	0.03
ENSG00000113212	PCDHB7	0.00	-8.40	0.03

ENSG00000255750	AC022509	0.12	-3.04	0.03
ENSG00000180354	MTURN	0.38	-1.40	0.03
ENSG00000176170	SPHK1	0.85	-0.23	0.03
ENSG00000092201	SUPT16H	0.85	-0.23	0.03
ENSG00000204816	FGF7P5	0.00	-12.09	0.03
ENSG00000099860	GADD45B	0.65	-0.63	0.03
ENSG00000143537	ADAM15	0.86	-0.22	0.03
ENSG00000122971	ACADS	0.68	-0.56	0.03
ENSG00000011523	CEP68	0.80	-0.33	0.03
ENSG00000163320	CGGBP1	0.78	-0.35	0.03
ENSG00000177225	GATD1	0.71	-0.48	0.03
ENSG00000113119	TMCO6	0.71	-0.49	0.03
ENSG00000079387	SENP1	0.74	-0.44	0.03
ENSG00000092010	PSME1	0.84	-0.25	0.03
ENSG00000107566	ERLIN1	0.85	-0.23	0.03
ENSG00000122694	GLIPR2	0.63	-0.68	0.03
ENSG00000186470	BTN3A2	0.67	-0.58	0.03
ENSG00000175183	CSRP2	0.73	-0.45	0.03
ENSG00000100568	VTI1B	0.85	-0.24	0.03
ENSG00000267426	AC087289	0.62	-0.68	0.03
ENSG00000055211	GINM1	0.80	-0.32	0.03
ENSG00000122778	KIAA1549	0.83	-0.27	0.03
ENSG00000163864	NMNAT3	0.41	-1.27	0.03
ENSG00000138002	IFT172	0.80	-0.32	0.03
ENSG00000204956	PCDHGA1	0.48	-1.05	0.03
ENSG00000148362	PAXX	0.72	-0.47	0.03
ENSG00000160271	RALGDS	0.80	-0.33	0.03
ENSG00000267023	LRRC37A16P	0.74	-0.44	0.03
ENSG00000234545	FAM133B	0.75	-0.41	0.03
ENSG00000241635	UGT1A1	0.38	-1.40	0.03
ENSG00000116754	SRSF11	0.83	-0.27	0.03
ENSG00000145014	TMEM44	0.78	-0.36	0.03
ENSG00000137414	FAM8A1	0.76	-0.40	0.03
ENSG00000158552	ZFAND2B	0.77	-0.38	0.03
ENSG00000250305	TRMT9B	0.31	-1.70	0.03
ENSG00000179240	GVQW3	0.48	-1.06	0.03
ENSG00000260625	AC026471	0.00	-10.37	0.03
ENSG00000070756	PABPC1	0.88	-0.18	0.03
ENSG00000172977	KAT5	0.82	-0.28	0.03
ENSG00000287731	AL035603	0.00	-9.52	0.03
ENSG00000111653	ING4	0.79	-0.34	0.03
ENSG00000100030	MAPK1	0.87	-0.20	0.03
ENSG00000112941	TENT4A	0.81	-0.30	0.03
ENSG00000166548	TK2	0.74	-0.43	0.03
ENSG00000167461	RAB8A	0.83	-0.27	0.03
ENSG00000179715	PCED1B	0.69	-0.54	0.03
ENSG00000130023	ERMARD	0.72	-0.48	0.03
ENSG00000165309	ARMC3	0.46	-1.13	0.03
ENSG00000150893	FREM2	0.33	-1.62	0.03
ENSG00000278189	RNA5-8SN1	0.07	-3.82	0.03
ENSG00000107669	ATE1	0.79	-0.34	0.03
ENSG00000136937	NCBP1	0.85	-0.23	0.03
ENSG00000101346	POFUT1	0.87	-0.20	0.03
ENSG00000274791	F8A2	0.69	-0.54	0.03
ENSG00000133265	HSPBP1	0.84	-0.25	0.03
ENSG00000171488	LRRC8C	0.70	-0.51	0.03
ENSG00000156162	DPY19L4	0.80	-0.32	0.03
ENSG00000185619	PCGF3	0.77	-0.38	0.03
ENSG00000168887	C2orf68	0.71	-0.50	0.03
ENSG00000163807	KIAA1143	0.77	-0.37	0.03
ENSG00000134882	UBAC2	0.46	-1.13	0.03

ENSG00000255843	AP000593	0.00	-11.19	0.03
ENSG00000009694	TENM1	0.14	-2.79	0.03
ENSG00000140262	TCF12	0.70	-0.51	0.03
ENSG00000277157	H4C4	0.00	-11.73	0.03
ENSG00000255182	AC084125	0.54	-0.89	0.03
ENSG00000157796	WDR19	0.79	-0.35	0.03
ENSG00000086015	MAST2	0.86	-0.22	0.03
ENSG00000189223	PAX8-AS1	0.81	-0.31	0.03
ENSG00000149231	CCDC82	0.76	-0.39	0.03
ENSG00000138101	DTNB	0.71	-0.50	0.03
ENSG00000227619	AL391056	0.00	-8.21	0.03
ENSG00000276150	AL031591	0.02	-5.80	0.03
ENSG00000134184	GSTM1	0.07	-3.86	0.03
ENSG00000150540	HNMT	0.73	-0.45	0.03
ENSG00000102805	CLN5	0.78	-0.35	0.03
ENSG00000187867	PALM3	0.08	-3.61	0.03
ENSG00000038219	BOD1L1	0.83	-0.26	0.03
ENSG00000005893	LAMP2	0.88	-0.19	0.03
ENSG00000170927	PKHD1	0.02	-5.38	0.03
ENSG00000106290	TAF6	0.83	-0.28	0.03
ENSG00000213918	DNASE1	0.75	-0.41	0.03
ENSG00000103550	KNOP1	0.85	-0.24	0.03
ENSG00000101004	NINL	0.70	-0.51	0.03
ENSG00000196943	NOP9	0.80	-0.32	0.03
ENSG00000124406	ATP8A1	0.36	-1.46	0.03
ENSG00000124134	KCNS1	0.57	-0.82	0.03
ENSG00000113319	RASGRF2	0.28	-1.85	0.03
ENSG00000163374	YY1AP1	0.86	-0.22	0.03
ENSG00000164841	TMEM74	0.40	-1.32	0.03
ENSG00000248008	NRAV	0.83	-0.27	0.03
ENSG00000176472	ZNF575	0.40	-1.32	0.03
ENSG00000115365	LANCL1	0.85	-0.24	0.03
ENSG00000100852	ARHGAP5	0.83	-0.27	0.03
ENSG00000134313	KIDINS220	0.80	-0.32	0.03
ENSG00000214110	LDHAP4	0.75	-0.41	0.03
ENSG00000137821	LRRC49	0.65	-0.62	0.03
ENSG00000187243	MAGED4B	0.75	-0.42	0.03
ENSG00000254477	AP000640	0.33	-1.60	0.03
ENSG00000085644	ZNF213	0.63	-0.66	0.03
ENSG00000287733	AC083862	0.53	-0.90	0.03
ENSG00000171792	RHNO1	0.73	-0.45	0.03
ENSG00000136247	ZDHHC4	0.79	-0.34	0.03
ENSG00000159110	IFNAR2	0.63	-0.66	0.03
ENSG00000143486	EIF2D	0.83	-0.26	0.03
ENSG00000160796	NBEAL2	0.79	-0.33	0.03
ENSG00000171988	JMJD1C	0.76	-0.40	0.03
ENSG00000157765	SLC34A2	0.13	-2.98	0.03
ENSG00000108424	KPNB1	0.83	-0.26	0.03
ENSG00000185485	SDHAP1	0.78	-0.37	0.03
ENSG00000112305	SMAP1	0.80	-0.33	0.03
ENSG00000235314	LINC00957	0.43	-1.20	0.03
ENSG00000004399	PLXND1	0.82	-0.29	0.03
ENSG00000182013	PNMA8A	0.80	-0.33	0.03
ENSG00000180233	ZNRF2	0.76	-0.40	0.03
ENSG00000164743	C8orf48	0.58	-0.78	0.03
ENSG00000204619	PPP1R11	0.86	-0.21	0.03
ENSG00000260244	AC104083	0.10	-3.34	0.03
ENSG00000102878	HSF4	0.67	-0.59	0.03
ENSG00000163714	U2SURP	0.84	-0.24	0.03
ENSG00000132024	CC2D1A	0.84	-0.24	0.03
ENSG00000170542	SERPINB9	0.55	-0.87	0.03

ENSG00000260597	AC012531	0.52	-0.95	0.03
ENSG00000179387	ELMOD2	0.76	-0.40	0.03
ENSG00000184226	PCDH9	0.35	-1.51	0.03
ENSG00000081019	RSBN1	0.77	-0.38	0.03
ENSG00000249773	AC092647	0.14	-2.84	0.03
ENSG00000110237	ARHGEF17	0.83	-0.26	0.03
ENSG00000158747	NBL1	0.86	-0.21	0.03
ENSG00000146197	SCUBE3	0.13	-2.99	0.03
ENSG00000099282	TSPAN15	0.77	-0.38	0.03
ENSG00000079150	FKBP7	0.72	-0.48	0.03
ENSG00000116649	SRM	0.87	-0.21	0.03
ENSG00000131470	PSMC3IP	0.75	-0.41	0.03
ENSG00000278921	EPB41L4A-DT	0.11	-3.21	0.03
ENSG00000182253	SYNM	0.83	-0.27	0.03
ENSG00000117600	PLPPR4	0.11	-3.24	0.03
ENSG00000121775	TMEM39B	0.78	-0.35	0.03
ENSG00000159069	FBXW5	0.84	-0.25	0.03
ENSG00000165124	SVEP1	0.34	-1.57	0.03
ENSG00000165714	BORCS5	0.43	-1.22	0.03
ENSG00000158528	PPP1R9A	0.54	-0.88	0.03
ENSG00000086232	EIF2AK1	0.85	-0.23	0.03
ENSG00000137642	SORL1	0.66	-0.59	0.03
ENSG00000197965	MPZL1	0.27	-1.86	0.03
ENSG00000226549	SCDP1	0.06	-4.12	0.03
ENSG00000047230	CTPS2	0.80	-0.33	0.03
ENSG00000136280	CCM2	0.78	-0.37	0.03
ENSG00000133243	BTBD2	0.85	-0.23	0.03
ENSG00000144791	LIMD1	0.82	-0.28	0.03
ENSG00000138741	TRPC3	0.08	-3.64	0.03
ENSG00000202252	SNORD14C	0.00	-8.68	0.03
ENSG00000155755	TMEM237	0.81	-0.30	0.03
ENSG00000229809	ZNF688	0.70	-0.52	0.03
ENSG00000143036	SLC44A3	0.43	-1.20	0.03
ENSG00000163545	NUAK2	0.79	-0.35	0.03
ENSG00000185522	LMNTD2	0.51	-0.97	0.03
ENSG00000140057	AK7	0.44	-1.17	0.03
ENSG00000198740	ZNF652	0.80	-0.32	0.03
ENSG00000151458	ANKRD50	0.84	-0.25	0.03
ENSG00000236698	EIF1AXP1	0.74	-0.44	0.03
ENSG00000178585	CTNNBIP1	0.71	-0.49	0.03
ENSG00000283795	MIR4426	0.05	-4.26	0.03
ENSG00000240032	LNCSRLR	0.24	-2.06	0.03
ENSG00000108349	CASC3	0.87	-0.20	0.03
ENSG00000170962	PDGFD	0.60	-0.73	0.03
ENSG00000165030	NFIL3	0.83	-0.27	0.03
ENSG00000186063	AIDA	0.80	-0.33	0.03
ENSG00000171928	TVP23B	0.80	-0.33	0.03
ENSG00000226200	SGMS1-AS1	0.59	-0.75	0.03
ENSG00000135480	KRT7	0.62	-0.69	0.03
ENSG00000205220	PSMB10	0.82	-0.29	0.03
ENSG00000227598	Z94721	0.00	-11.53	0.03
ENSG00000269425	AC104521	0.00	-12.02	0.03
ENSG00000167393	PPP2R3B	0.66	-0.59	0.03
ENSG00000077279	DCX	0.01	-6.82	0.03
ENSG00000163273	NPPC	0.00	-10.06	0.03
ENSG00000133985	TTC9	0.00	-7.82	0.03
ENSG00000230409	TCEA1P2	0.51	-0.97	0.03
ENSG00000105792	CFAP69	0.40	-1.32	0.03
ENSG00000100888	CHD8	0.81	-0.31	0.03
ENSG00000265413	AP001094	0.37	-1.45	0.03
ENSG00000119402	FBXW2	0.86	-0.22	0.03

ENSG00000162430	SELENON	0.86	-0.22	0.03
ENSG00000134258	VTCN1	0.00	-8.57	0.03
ENSG00000243135	UGT1A3	0.00	-8.91	0.03
ENSG00000224083	MTCO1P11	0.00	-9.54	0.03
ENSG00000141542	RAB40B	0.72	-0.47	0.03
ENSG00000115884	SDC1	0.85	-0.24	0.03
ENSG00000173599	PC	0.84	-0.26	0.03
ENSG00000117616	RSRP1	0.63	-0.67	0.03
ENSG00000130382	MLLT1	0.88	-0.18	0.03
ENSG00000269534	AC011466	0.00	-10.22	0.03
ENSG00000082641	NFE2L1	0.78	-0.35	0.03
ENSG00000196504	PRPF40A	0.85	-0.24	0.03
ENSG00000100564	PIGH	0.60	-0.73	0.03
ENSG00000196436	NPIPB15	0.54	-0.90	0.03
ENSG00000234602	MCIDAS	0.34	-1.54	0.03
ENSG00000138036	DYNC2LI1	0.70	-0.52	0.03
ENSG00000262160	AC020978	0.00	-9.47	0.03
ENSG00000204131	NHSL2	0.11	-3.21	0.03
ENSG00000103723	AP3B2	0.01	-6.71	0.03
ENSG00000118017	A4GNT	0.20	-2.36	0.03
ENSG00000263961	RHEX	0.84	-0.26	0.03
ENSG00000100889	PCK2	0.80	-0.33	0.03
ENSG00000134548	SPX	0.43	-1.22	0.03
ENSG00000237973	MTCO1P12	0.87	-0.20	0.03
ENSG00000071073	MGAT4A	0.72	-0.47	0.03
ENSG00000147421	HMBOX1	0.61	-0.71	0.03
ENSG00000263513	FAM72C	0.80	-0.33	0.03
ENSG00000163378	EOGT	0.69	-0.53	0.03
ENSG00000166900	STX3	0.83	-0.26	0.03
ENSG00000179627	ZBTB42	0.60	-0.74	0.03
ENSG00000167861	HID1	0.28	-1.83	0.03
ENSG00000117984	CTSD	0.85	-0.23	0.03
ENSG00000180902	D2HGDH	0.71	-0.50	0.03
ENSG00000205084	TMEM231	0.81	-0.31	0.03
ENSG00000255856	AC069503	0.00	-9.93	0.03
ENSG00000088256	GNA11	0.84	-0.26	0.03
ENSG00000151502	VPS26B	0.86	-0.22	0.03
ENSG00000156650	KAT6B	0.76	-0.40	0.03
ENSG00000163931	TKT	0.89	-0.17	0.03
ENSG00000140983	RHOT2	0.84	-0.25	0.03
ENSG00000090924	PLEKHG2	0.84	-0.25	0.04
ENSG00000168806	LCMT2	0.75	-0.41	0.04
ENSG00000070814	TCOF1	0.88	-0.18	0.04
ENSG00000129810	SGO1	0.72	-0.48	0.04
ENSG00000272990	AC084036	0.21	-2.23	0.04
ENSG00000168517	HEXIM2	0.70	-0.51	0.04
ENSG00000178149	DALRD3	0.78	-0.36	0.04
ENSG00000135976	ANKRD36	0.63	-0.68	0.04
ENSG00000138801	PAPSS1	0.84	-0.24	0.04
ENSG00000070047	PHRF1	0.86	-0.21	0.04
ENSG00000091986	CCDC80	0.53	-0.91	0.04
ENSG00000137814	HAUS2	0.71	-0.48	0.04
ENSG00000149182	ARFGAP2	0.87	-0.20	0.04
ENSG00000146416	AIG1	0.79	-0.33	0.04
ENSG00000169946	ZFPM2	0.64	-0.65	0.04
ENSG00000259577	CERNA1	0.00	-8.56	0.04
ENSG00000261141	AC092718	0.00	-11.05	0.04
ENSG00000144134	RABL2A	0.68	-0.55	0.04
ENSG00000166130	IKBIP	0.86	-0.22	0.04
ENSG00000285437	POLR2J3	0.54	-0.90	0.04
ENSG00000166845	C18orf54	0.58	-0.79	0.04

ENSG00000188312	CENPP	0.76	-0.40	0.04
ENSG00000272150	NBPF25P	0.65	-0.61	0.04
ENSG00000196810	CTBP1-DT	0.72	-0.48	0.04
ENSG00000010361	FUZ	0.65	-0.63	0.04
ENSG00000288637	AC013477	0.26	-1.94	0.04
ENSG00000115808	STRN	0.79	-0.33	0.04
ENSG00000162929	KIAA1841	0.66	-0.59	0.04
ENSG00000244474	UGT1A4	0.04	-4.76	0.04
ENSG00000019144	PHLDB1	0.83	-0.27	0.04
ENSG00000069849	ATP1B3	0.75	-0.42	0.04
ENSG00000165512	ZNF22	0.79	-0.34	0.04
ENSG00000135999	EPC2	0.80	-0.31	0.04
ENSG00000135164	DMTF1	0.80	-0.33	0.04
ENSG00000172244	C5orf34	0.71	-0.49	0.04
ENSG00000160209	PDXK	0.87	-0.20	0.04
ENSG00000184678	H2BC21	0.68	-0.57	0.04
ENSG00000115355	CCDC88A	0.81	-0.30	0.04
ENSG00000274600	RIMBP3B	0.00	-8.49	0.04
ENSG00000120256	LRP11	0.78	-0.36	0.04
ENSG00000006747	SCIN	0.80	-0.33	0.04
ENSG00000082458	DLG3	0.66	-0.60	0.04
ENSG00000166037	CEP57	0.76	-0.40	0.04
ENSG00000162437	RAVER2	0.78	-0.37	0.04
ENSG00000281383	FP671120	0.23	-2.15	0.04
ENSG00000226193	AL049548	0.00	-11.61	0.04
ENSG00000163029	SMC6	0.79	-0.33	0.04
ENSG00000172493	AFF1	0.54	-0.88	0.04
ENSG00000112319	EYA4	0.74	-0.44	0.04
ENSG00000233006	MIR3936HG	0.53	-0.92	0.04
ENSG00000174720	LARP7	0.78	-0.36	0.04
ENSG00000253366	GUSBP16	0.40	-1.33	0.04
ENSG00000185127	C6orf120	0.80	-0.32	0.04
ENSG00000099904	ZDHHC8	0.81	-0.30	0.04
ENSG00000006282	SPATA20	0.86	-0.22	0.04
ENSG00000130227	XPO7	0.88	-0.18	0.04
ENSG00000153250	RBMS1	0.86	-0.23	0.04
ENSG00000122644	ARL4A	0.64	-0.63	0.04
ENSG00000171119	NRTN	0.18	-2.48	0.04
ENSG00000176209	SMIM19	0.66	-0.61	0.04
ENSG00000144645	OSBPL10	0.78	-0.36	0.04
ENSG00000119685	TTLL5	0.72	-0.48	0.04
ENSG00000259498	TPM1-AS	0.49	-1.02	0.04
ENSG00000198106	AC025279	0.00	-7.91	0.04
ENSG00000166348	USP54	0.81	-0.30	0.04
ENSG00000185829	ARL17A	0.78	-0.35	0.04
ENSG00000123200	ZC3H13	0.86	-0.21	0.04
ENSG00000146085	MMUT	0.82	-0.29	0.04
ENSG00000108055	SMC3	0.82	-0.29	0.04
ENSG00000198952	SMG5	0.89	-0.17	0.04
ENSG00000279789	AC120114	0.65	-0.63	0.04
ENSG00000227684	CROCCP4	0.00	-10.43	0.04
ENSG00000270977	AC015849	0.24	-2.05	0.04
ENSG00000100485	SOS2	0.71	-0.49	0.04
ENSG00000229117	RPL41	0.89	-0.16	0.04
ENSG00000198931	APRT	0.84	-0.26	0.04
ENSG00000102699	PARP4	0.87	-0.19	0.04
ENSG00000279044	AC007787	0.09	-3.51	0.04
ENSG00000275016	AC015574	0.01	-7.14	0.04
ENSG00000039600	SOX30	0.00	-8.38	0.04
ENSG00000134698	AGO4	0.78	-0.36	0.04
ENSG00000283549	AC007161	0.07	-3.86	0.04

ENSG00000147654	EBAG9	0.73	-0.46	0.04
ENSG00000151176	PLBD2	0.87	-0.20	0.04
ENSG00000185942	NKAIN3	0.03	-5.23	0.04
ENSG00000116544	DLGAP3	0.00	-8.10	0.04
ENSG00000246596	AC139795	0.50	-1.00	0.04
ENSG00000142784	WDTC1	0.86	-0.22	0.04
ENSG00000179051	RCC2	0.87	-0.20	0.04
ENSG00000163964	PIGX	0.81	-0.30	0.04
ENSG00000173207	CKS1B	0.87	-0.20	0.04
ENSG00000245248	USP2-AS1	0.40	-1.33	0.04
ENSG00000187398	LUZP2	0.01	-7.12	0.04
ENSG00000179262	RAD23A	0.83	-0.26	0.04
ENSG00000064666	CNN2	0.86	-0.21	0.04
ENSG00000116641	DOCK7	0.85	-0.24	0.04
ENSG00000143324	XPR1	0.87	-0.21	0.04
ENSG00000101306	MYLK2	0.11	-3.15	0.04
ENSG00000163935	SFMBT1	0.67	-0.57	0.04
ENSG00000010244	ZNF207	0.88	-0.18	0.04
ENSG00000168061	SAC3D1	0.79	-0.35	0.04
ENSG00000198964	SGMS1	0.79	-0.34	0.04
ENSG00000246523	FZD4-DT	0.09	-3.50	0.04
ENSG00000163607	GTPBP8	0.73	-0.46	0.04
ENSG00000204174	NPY4R	0.00	-9.09	0.04
ENSG00000165494	PCF11	0.85	-0.24	0.04
ENSG00000106392	C1GALT1	0.80	-0.33	0.04
ENSG00000196482	ESRRG	0.48	-1.06	0.04
ENSG00000267892	AC022144	0.14	-2.81	0.04
ENSG00000276550	HERC2P2	0.84	-0.25	0.04
ENSG00000155111	CDK19	0.80	-0.33	0.04
ENSG00000078177	N4BP2	0.72	-0.48	0.04
ENSG00000281181	FP236383	0.24	-2.09	0.04
ENSG00000159714	ZDHHC1	0.76	-0.39	0.04
ENSG00000230432	AC114803	0.00	-11.83	0.04
ENSG00000129195	PIMREG	0.82	-0.29	0.04
ENSG00000101040	ZMYND8	0.87	-0.20	0.04
ENSG00000272221	AL645933	0.23	-2.11	0.04
ENSG00000165121	AL353743	0.42	-1.27	0.04
ENSG00000160392	C19orf47	0.64	-0.65	0.04
ENSG00000143499	SMYD2	0.87	-0.20	0.04
ENSG00000189423	USP32P3	0.58	-0.79	0.04
ENSG00000196782	MAML3	0.60	-0.73	0.04
ENSG00000136811	ODF2	0.84	-0.25	0.04
ENSG00000014164	ZC3H3	0.85	-0.24	0.04
ENSG00000119782	FKBP1B	0.51	-0.98	0.04
ENSG00000182208	MOB2	0.83	-0.27	0.04
ENSG00000241769	LINC00893	0.40	-1.32	0.04
ENSG00000236890	AC016027	0.00	-12.31	0.04
ENSG00000221890	NPTXR	0.87	-0.20	0.04
ENSG00000134115	CNTN6	0.33	-1.62	0.04
ENSG00000115524	SF3B1	0.87	-0.21	0.04
ENSG00000163344	PMVK	0.84	-0.25	0.04
ENSG00000106526	ACTR3C	0.54	-0.90	0.04
ENSG00000143801	PSEN2	0.83	-0.27	0.04
ENSG00000074276	CDHR2	0.33	-1.61	0.04
ENSG00000198924	DCLRE1A	0.78	-0.36	0.04
ENSG00000272374	Z97832	0.47	-1.07	0.04
ENSG00000254479	SLC25A1P1	0.00	-10.17	0.04
ENSG00000132475	H3-3B	0.86	-0.21	0.04
ENSG00000117862	TXNDC12	0.86	-0.22	0.04
ENSG00000137193	PIM1	0.82	-0.28	0.04
ENSG00000213096	ZNF254	0.49	-1.04	0.04

ENSG00000278730	AC005332	0.83	-0.26	0.04
ENSG00000242348	RN7SL561P	0.00	-11.97	0.04
ENSG00000135387	CAPRIN1	0.88	-0.19	0.04
ENSG00000176749	CDK5R1	0.62	-0.70	0.04
ENSG00000198860	TSEN15	0.80	-0.32	0.04
ENSG00000152422	XRCC4	0.75	-0.42	0.04
ENSG00000096717	SIRT1	0.80	-0.33	0.04
ENSG00000167528	ZNF641	0.75	-0.42	0.04
ENSG00000204843	DCTN1	0.88	-0.18	0.04
ENSG00000177663	IL17RA	0.81	-0.30	0.04
ENSG00000238258	AL121748	0.13	-3.00	0.04
ENSG00000266934	AC005746	0.06	-3.97	0.04
ENSG00000104915	STX10	0.80	-0.31	0.04
ENSG00000177239	MAN1B1	0.87	-0.21	0.04
ENSG00000175581	MRPL48	0.80	-0.32	0.04
ENSG00000182551	ADI1	0.83	-0.27	0.04
ENSG00000140564	FURIN	0.88	-0.19	0.04
ENSG00000267104	TBC1D3P1-DHX40P	0.08	-3.71	0.04
ENSG00000229334	AC046143	0.53	-0.91	0.04
ENSG00000280800	FP671120	0.23	-2.10	0.04
ENSG00000136238	RAC1	0.89	-0.16	0.04
ENSG00000163950	SLBP	0.87	-0.20	0.04
ENSG00000287560	AL731533	0.46	-1.11	0.04
ENSG00000126464	PRR12	0.87	-0.20	0.04
ENSG00000100162	CENPM	0.77	-0.38	0.04
ENSG00000146776	ATXN7L1	0.70	-0.51	0.04
ENSG00000185787	MORF4L1	0.83	-0.27	0.04
ENSG00000113558	SKP1	0.88	-0.18	0.04
ENSG00000228253	MT-ATP8	0.88	-0.18	0.04
ENSG00000100941	PNN	0.83	-0.27	0.04
ENSG00000089597	GANAB	0.88	-0.19	0.04
ENSG00000099250	NRP1	0.77	-0.37	0.04
ENSG00000140104	CLBA1	0.68	-0.55	0.04
ENSG00000070018	LRP6	0.82	-0.29	0.04
ENSG00000160703	NLRX1	0.82	-0.28	0.04
ENSG00000279289	AL136164	0.48	-1.06	0.04
ENSG00000166479	TMX3	0.82	-0.28	0.04
ENSG00000174125	TLR1	0.67	-0.58	0.04
ENSG00000272391	POM121C	0.88	-0.18	0.04
ENSG00000156508	EEF1A1	0.90	-0.15	0.04
ENSG00000184986	TMEM121	0.43	-1.21	0.04
ENSG00000143315	PIGM	0.83	-0.27	0.04
ENSG00000110455	ACCS	0.74	-0.44	0.04
ENSG00000228559	AL033519	0.00	-9.34	0.04
ENSG00000279329	AC020910	0.36	-1.46	0.04
ENSG00000120690	ELF1	0.78	-0.36	0.04
ENSG00000115998	C2orf42	0.73	-0.46	0.04
ENSG00000185946	RNPC3	0.79	-0.34	0.04
ENSG00000216775	AL109918	0.62	-0.69	0.04
ENSG00000090971	NAT14	0.81	-0.30	0.04
ENSG00000279833	AL031846	0.35	-1.53	0.04
ENSG00000133226	SRRM1	0.88	-0.19	0.04
ENSG00000273259	AL049839	0.00	-9.36	0.04
ENSG00000101773	RBBP8	0.83	-0.26	0.04
ENSG00000124702	KLHDC3	0.88	-0.19	0.04
ENSG00000154529	CNTNAP3B	0.40	-1.32	0.04
ENSG00000107249	GLIS3	0.82	-0.29	0.04
ENSG00000267436	AC005786	0.68	-0.56	0.04
ENSG00000006062	MAP3K14	0.84	-0.25	0.04
ENSG00000203952	CCDC160	0.38	-1.38	0.04
ENSG00000238755	LINC02006	0.00	-8.53	0.05

ENSG00000288061	AP000926	0.00	-10.51	0.05
ENSG00000249532	MIR302CHG	0.00	-10.46	0.05
ENSG00000121879	PIK3CA	0.80	-0.33	0.05
ENSG00000287762	AC108050	0.26	-1.92	0.05
ENSG00000182712	CMC4	0.71	-0.49	0.05
ENSG00000173821	RNF213	0.89	-0.16	0.05
ENSG00000196405	EVL	0.73	-0.45	0.05
ENSG00000157368	IL34	0.13	-2.95	0.05
ENSG00000089199	CHGB	0.14	-2.86	0.05
ENSG00000140044	JDP2	0.72	-0.47	0.05
ENSG00000288258	BX470111	0.00	-11.08	0.05
ENSG00000275191	AC007497	0.00	-10.88	0.05
ENSG00000135409	AMHR2	0.00	-8.09	0.05
ENSG00000199293	SNORA21	0.04	-4.51	0.05
ENSG00000109339	MAPK10	0.55	-0.86	0.05
ENSG00000238109	RNF14P3	0.00	-9.53	0.05
ENSG00000157021	CIBAR1P1	0.62	-0.68	0.05
ENSG00000111602	TIMELESS	0.87	-0.20	0.05
ENSG00000149506	ZP1	0.45	-1.14	0.05
ENSG00000163162	RNF149	0.87	-0.20	0.05
ENSG00000107560	RAB11FIP2	0.77	-0.38	0.05
ENSG00000177646	ACAD9	0.86	-0.21	0.05
ENSG00000271474	AC106881	0.15	-2.75	0.05
ENSG00000114026	OGG1	0.77	-0.37	0.05
ENSG00000244754	N4BP2L2	0.81	-0.30	0.05
ENSG00000173535	TNFRSF10C	0.20	-2.34	0.05
ENSG00000166321	NUDT13	0.66	-0.61	0.05
ENSG00000198744	MTCO3P12	0.63	-0.66	0.05
ENSG00000170667	RASA4B	0.63	-0.68	0.05
ENSG00000161265	U2AF1L4	0.63	-0.68	0.05
ENSG00000241852	C8orf58	0.79	-0.33	0.05
ENSG00000196312	MFSD14C	0.83	-0.26	0.05
ENSG00000234857	HNRNPUL2-BSCL2	0.20	-2.31	0.05
ENSG00000189184	PCDH18	0.58	-0.78	0.05
ENSG00000088881	EBF4	0.09	-3.41	0.05
ENSG00000140830	TXNL4B	0.79	-0.34	0.05
ENSG00000112701	SENP6	0.81	-0.30	0.05
ENSG00000259426	AC027237	0.38	-1.39	0.05
ENSG00000157423	HYDIN	0.33	-1.61	0.05
ENSG00000280614	FP236383	0.24	-2.05	0.05
ENSG00000152193	OBI1	0.80	-0.32	0.05
ENSG00000149212	SESN3	0.61	-0.72	0.05
ENSG00000131797	CLUHP3	0.50	-1.00	0.05
ENSG00000068784	SRBD1	0.73	-0.46	0.05
ENSG00000146410	MTFR2	0.69	-0.54	0.05
ENSG00000142856	ITGB3BP	0.80	-0.32	0.05
ENSG00000185418	TARS3	0.79	-0.35	0.05
ENSG00000239474	KLHL41	0.00	-8.62	0.05
ENSG00000138944	SHISAL1	0.81	-0.31	0.05
ENSG00000118961	LDAH	0.75	-0.42	0.05
ENSG00000147364	FBXO25	0.82	-0.29	0.05
ENSG00000125945	ZNF436	0.80	-0.32	0.05
ENSG00000132773	TOE1	0.82	-0.28	0.05
ENSG00000107159	CA9	0.12	-3.12	0.05
ENSG00000071794	HLTF	0.77	-0.38	0.05
ENSG00000164649	CDCA7L	0.80	-0.32	0.05
ENSG00000109736	MFSD10	0.86	-0.22	0.05
ENSG00000188343	CIBAR1	0.77	-0.38	0.05
ENSG00000112081	SRSF3	0.88	-0.18	0.05
ENSG00000178927	CYBC1	0.81	-0.30	0.05
ENSG00000173588	CEP83	0.69	-0.54	0.05

ENSG00000115568	ZNF142	0.81	-0.30	0.05
ENSG00000162896	PIGR	0.40	-1.32	0.05
ENSG00000267303	AC011511	0.00	-13.03	0.05
ENSG00000156026	MCU	0.87	-0.21	0.05
ENSG00000029534	ANK1	0.32	-1.65	0.05
ENSG00000263006	ROCK1P1	0.51	-0.97	0.05
ENSG00000230699	AL645608	0.17	-2.57	0.05
ENSG00000113456	RAD1	0.81	-0.30	0.05
ENSG00000176826	FKBP9P1	0.38	-1.40	0.05
ENSG00000282826	FRG1CP	0.76	-0.40	0.05
ENSG00000136274	NACAD	0.21	-2.26	0.05
ENSG00000185885	IFITM1	0.53	-0.93	0.05
ENSG00000101193	GID8	0.87	-0.21	0.05
ENSG00000275215	RNA5-8SN3	0.12	-3.01	0.05
ENSG00000215063	RPL21P2	0.00	-11.05	0.05
ENSG00000251562	MALAT1	0.49	-1.04	0.05
ENSG00000099800	TIMM13	0.87	-0.20	0.05
ENSG00000152475	ZNF837	0.57	-0.82	0.05
ENSG00000140526	ABHD2	0.87	-0.20	0.05
